# Supplementary figures and images for: Mung bean seed classification based on multimodal features and Kepler-optimized stacking ensemble learning model
Source: PLoS One. 2026 Jan 5;21(1):e0338928. doi: 10.1371/journal.pone.0338928 (PMC12768288; doi:10.1371/journal.pone.0338928)

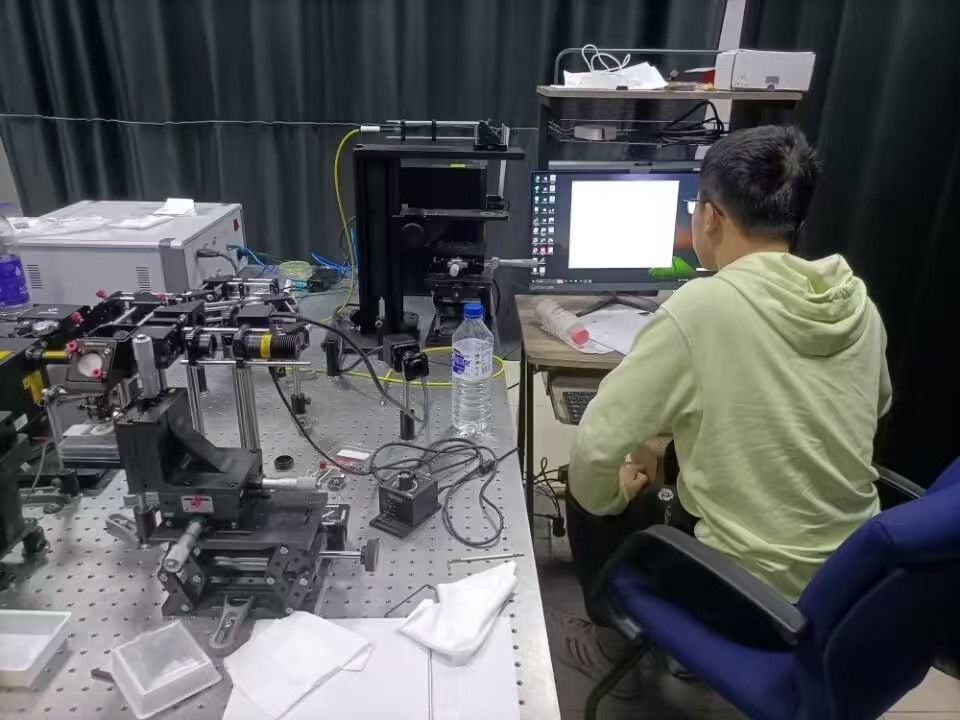

Supplement: S1 Appendix — (JPG) [file pone.0338928.s001.jpg]

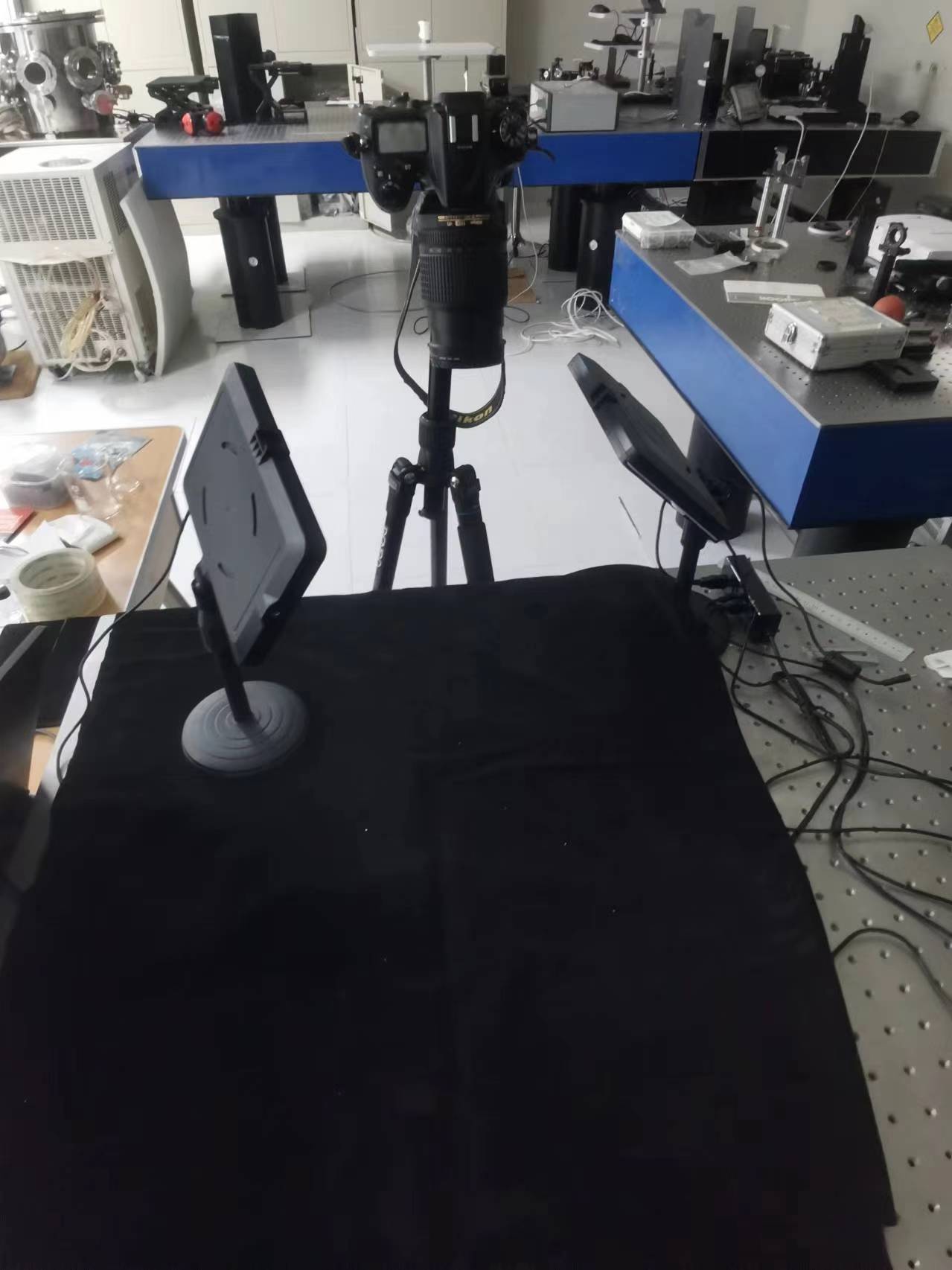

Supplement: S2 Appendix — (JPG) [file pone.0338928.s002.jpg]
