# Supplementary material for: Mung bean seed classification based on multimodal features and Kepler-optimized stacking ensemble learning model
Source: PLoS One. 2026 Jan 5;21(1):e0338928. doi: 10.1371/journal.pone.0338928 (PMC12768288; doi:10.1371/journal.pone.0338928)
Supplement: Attachment — Submitted filename: PONE-D-24-59080_R1_AG.pdf [file pone.0338928.s004.pdf]

PLOS ONE

Mung bean seed classification based on multimodal features and Kepler-optimized stacking ensemble learning model

--Manuscript Draft--

**Manuscript Number:**	PONE-D-24-59080R1
**Article Type:**	Research Article
**Full Title:**	Mung bean seed classification based on multimodal features and Kepler-optimized stacking ensemble learning model
**Short Title:**	Mung bean seed classification based on stacking ensemble model
**Corresponding Author:**	Xiaofeng An<br>Jilin Engineering Normal University<br>Changchun, Jilin CHINA
**Keywords:**	stacking ensemble learning model, Kepler optimization algorithm, feature selection, multimodal features, Raman spectroscopy, mung bean seed classification.
**Abstract:**	Abstract<br>Accurate classification of mung bean seeds is essential for enhancing both their nutritional value and crop yields. However, current methods are limited, primarily due to the time-consuming and inaccurate classification process resulting from a lack of diverse dataset features. To overcome these challenges, this study develops a multimodal dataset that integrates Raman spectral features and image-based features through early fusion. Furthermore, the classification of mung bean seed varieties is achieved in a rapid, accurate, and non-destructive manner by optimizing a stacking ensemble learning model using the Kepler Optimization Algorithm (KOA). The multimodal dataset comprises 59 features, selected using the Competitive Adaptive Reweighted Sampling (CARS) method. Specifically, 44 key features are extracted from 700 Raman spectral data points, while 15 key features are derived from 43 image-based features. The study also used the Kepler Optimization Algorithm to optimize the parameters of various machine learning models, including Decision Tree (DT), Support Vector Machine (SVM), k-Nearest Neighbor (KNN), Backpropagation Neural Network (BPNN), Random Forest (RF), and Gradient Boosting Decision Tree (GBDT). By constructing a stacking ensemble learning model, the research effectively leverages the strengths of multiple classifiers, thereby enhancing the overall classification performance. Experimental results demonstrate that the proposed method significantly improves mung bean seed classification accuracy, with the Kepler-optimized stacking ensemble model achieving an accuracy of 90.71%. This represents a 3.24% improvement over KOA-RF and a 1.59% improvement over KOA-GBDT. In comparison to baseline models, the proposed method proves to be more efficient.This study underscores the potential of combining multimodal features with a Kepler-optimized stacking ensemble learning model for mung bean seed classification. It highlights the role of advanced artificial intelligence techniques in agricultural production and provides valuable technical support for the precise classification of mung bean seeds.
**Order of Authors:**	Xiaofeng An
	Shaozhong Song
	Fengwei Leng
	Ming Fang
	Yaixin Cai
**Response to Reviewers:**	Point 1:When submitting your revision, we need you to address these additional requirements.Please ensure that your manuscript meets PLOS ONE's style requirements, including those for file naming.<br>Response 1:Thanks for your valuable comments.I formatted the article according to the journal's guidelines.<br>For example:Added line numbers to the title<br><br>Point 2:Please note that PLOS ONE has specific guidelines on code sharing for

Powered by Editorial Manager® and ProduXion Manager® from Aries Systems Corporation

	submissions in which author-generated code underpins the findings in the manuscript. In these cases, we expect all author-generated code to be made available without restrictions upon publication of the work.<br>Response 2:Thank you for your valuable comments. In response, I have uploaded the complete code implementation with comprehensive annotations to GitHub. Both the source code and corresponding datasets are included in the same repository to ensure full reproducibility for the research community.<br>GitHub:https://github.com/781118954/multi-data<br><br>Point 3:Please include captions for your Supporting Information files at the end of your manuscript, and update any in-text citations to match accordingly.<br>Response 3:Thanks for your valuable comments.I have added the captions for the Supporting Information at the end of the manuscript and made the necessary in-text citations accordingly.<br>1.Captions for Supporting information<br>2.Corresponding in-text citations<br><br>Point 4(Additional Editor Comments):The images are completely blurred, making it difficult to interpret the visual data accurately. Additionally, the modeling of the proposed method requires further clarification. It should be explained in a way that enhances understanding and benefits readers from various backgrounds. Please address these issues carefully to ensure that the manuscript meets the quality standards of PLOS ONE.<br>Response 4:Thank you for your valuable feedback. Regarding the image resolution issue, I have updated the figure files using the Preflight Analysis and Conversion Engine (PACE) digital diagnostic tool provided by the journal, and they have been successfully re-uploaded. Additionally, the original composite image has been split into individual images and uploaded separately.<br>To address the need for further clarification of the modeling process, a detailed explanation has been incorporated between the theoretical framework and experimental sections to ensure comprehensibility for readers across diverse disciplinary backgrounds.<br>After verification：<br>3.Results and discussion<br>The stacking ensemble learning model adopted in this paper consists of two layers: base learners and a meta-learner. It fuses the prediction results of multiple base learners of different types and then conducts final integration through the meta-learner. By leveraging the complementarity among models, it captures the characteristics and patterns of data from multiple perspectives, effectively reducing errors and significantly improving the accuracy and generalization ability of prediction and classification. In view of this, selecting high - performing base learner models and meta-learner models is of great importance. In this paper, the KOA is first applied to optimize the parameters of six classification models,including SVM, DT, KNN, RF, MLP, and AdaBoost. The optimized models are then used as the base learners of the stacking ensemble learning model. Subsequently, the meta-learner model is selected through a combinatorial optimization approach, and finally, the stacking ensemble learning model used in this paper is constructed.<br>The hardware specifications employed in this experiment are as follows: the processor is an Intel Core i5-12400F with a clock speed of 2.50 GHz, the system is equipped with 16 GB of RAM, and the graphics card utilized is an NVIDIA RTX 4060 Ti.
**Additional Information:**	
**Question**	**Response**
**Financial Disclosure**<br><br>Enter a financial disclosure statement that describes the sources of funding for the work included in this submission. Review the submission guidelines for detailed requirements. View published research articles from *PLOS ONE* for specific	Yes

examples.<br><br>This statement is required for submission and **will appear in the published article** if the submission is accepted. Please make sure it is accurate.<br><br>**Funded studies**<br>Enter a statement with the following details:<br>• Initials of the authors who received each award<br>• Grant numbers awarded to each author<br>• The full name of each funder<br>• URL of each funder website<br>• Did the sponsors or funders play any role in the study design, data collection and analysis, decision to publish, or preparation of the manuscript?<br><br>Did you receive funding for this work?	
Please add funding details.<br>as follow-up to "**Financial Disclosure**<br><br>Enter a financial disclosure statement that describes the sources of funding for the work included in this submission. Review the submission guidelines for detailed requirements. View published research articles from *PLOS ONE* for specific examples.<br><br>This statement is required for submission and **will appear in the published article** if the submission is accepted. Please make sure it is accurate.<br><br>**Funded studies**<br>Enter a statement with the following details:<br>• Initials of the authors who received each award<br>• Grant numbers awarded to each author<br>• The full name of each funder<br>• URL of each funder website<br>• Did the sponsors or funders play any role in the study design, data collection and analysis, decision to publish, or preparation of the manuscript?	SS<br>1. The Natural Science Foundation of Jilin Province (No.2020122348JC)<br>Shaozhong Song<br>It played a role in the study design, data collection and analysis.

Powered by Editorial Manager® and ProduXion Manager® from Aries Systems Corporation

Did you receive funding for this work?"	
Please select the country of your main research funder (please select carefully as in some cases this is used in fee calculation).<br>as follow-up to "**Financial Disclosure**<br><br>Enter a financial disclosure statement that describes the sources of funding for the work included in this submission. Review the submission guidelines for detailed requirements. View published research articles from *PLOS ONE* for specific examples.<br><br>This statement is required for submission and **will appear in the published article** if the submission is accepted. Please make sure it is accurate.<br><br>**Funded studies**<br>Enter a statement with the following details:<br>• Initials of the authors who received each award<br>• Grant numbers awarded to each author<br>• The full name of each funder<br>• URL of each funder website<br>• Did the sponsors or funders play any role in the study design, data collection and analysis, decision to publish, or preparation of the manuscript?<br><br>Did you receive funding for this work?"	CHINA - CN
**Competing Interests**<br><br>Use the instructions below to enter a competing interest statement for this submission. On behalf of all authors, disclose any competing interests that could be perceived to bias this work—acknowledging all financial support and any other relevant financial or non-financial competing interests.	The authors have declared that no competing interests exist.

This statement is required for submission and **will appear in the published article** if the submission is accepted. Please make sure it is accurate and that any funding sources listed in your Funding Information later in the submission form are also declared in your Financial Disclosure statement.<br><br>View published research articles from *PLOS ONE* for specific examples.<br><br>**NO authors have competing interests**<br><br>Enter: *The authors have declared that no competing interests exist.*<br><br>**Authors with competing interests**<br><br>Enter competing interest details beginning with this statement:<br><br>*I have read the journal's policy and the authors of this manuscript have the following competing interests: [insert competing interests here]*<br><br>* typeset	
**Ethics Statement**<br><br>Enter an ethics statement for this submission. This statement is required if the study involved:<br><br>• Human participants<br>• Human specimens or tissue<br>• Vertebrate animals or cephalopods<br>• Vertebrate embryos or tissues<br>• Field research<br><br>Write "N/A" if the submission does not require an ethics statement.<br><br>General guidance is provided below. Consult the submission guidelines for detailed instructions. **Make sure that all information entered here is included in the Methods section of the manuscript.**	N/A

**Format for specific study types**<br><br>**Human Subject Research (involving human participants and/or tissue)**<br>• Give the name of the institutional review board or ethics committee that approved the study<br>• Include the approval number and/or a statement indicating approval of this research<br>• Indicate the form of consent obtained (written/oral) or the reason that consent was not obtained (e.g. the data were analyzed anonymously)<br><br>**Animal Research (involving vertebrate animals, embryos or tissues)**<br>• Provide the name of the Institutional Animal Care and Use Committee (IACUC) or other relevant ethics board that reviewed the study protocol, and indicate whether they approved this research or granted a formal waiver of ethical approval<br>• Include an approval number if one was obtained<br>• If the study involved *non-human primates*, add *additional details* about animal welfare and steps taken to ameliorate suffering<br>• If anesthesia, euthanasia, or any kind of animal sacrifice is part of the study, include briefly which substances and/or methods were applied<br><br>**Field Research**<br><br>Include the following details if this study involves the collection of plant, animal, or other materials from a natural setting:<br>• Field permit number<br>• Name of the institution or relevant body that granted permission	
**Data Availability**<br><br>Authors are required to make all data underlying the findings described fully available, without restriction, and from the time of publication. PLOS allows rare exceptions to address legal and ethical concerns. See the PLOS Data Policy and FAQ for detailed information.	Yes - all data are fully available without restriction

A Data Availability Statement describing where the data can be found is required at submission. Your answers to this question constitute the Data Availability Statement and **will be published in the article**, if accepted.<br><br>**Important:** Stating 'data available on request from the author' is not sufficient. If your data are only available upon request, select 'No' for the first question and explain your exceptional situation in the text box.<br><br>Do the authors confirm that all data underlying the findings described in their manuscript are fully available without restriction?	
**Describe where the data may be found in full sentences. If you are copying our sample text, replace any instances of XXX with the appropriate details.**<br><br>• If the data are **held or will be held in a public repository**, include URLs, accession numbers or DOIs. If this information will only be available after acceptance, indicate this by ticking the box below. For example: *All XXX files are available from the XXX database (accession number(s) XXX, XXX.).*<br>• If the data are all contained **within the manuscript and/or Supporting Information files**, enter the following: *All relevant data are within the manuscript and its Supporting Information files.*<br>• If neither of these applies but you are able to provide **details of access elsewhere**, with or without limitations, please do so. For example:<br><br>*Data cannot be shared publicly because of [XXX]. Data are available from the XXX Institutional Data Access / Ethics Committee (contact via XXX) for researchers who meet the criteria for access to confidential data.*<br><br>*The data underlying the results presented in the study are available*	The data used in this study are publicly available on the GitHub platform. The dataset can be accessed via the following link: https://github.com/781118954/multi-data. All relevant data are openly available to ensure the reproducibility of this study.

Powered by Editorial Manager® and ProduXion Manager® from Aries Systems Corporation

from (include the name of the third party and contact information or URL).<br>• This text is appropriate if the data are owned by a third party and authors do not have permission to share the data.<br><br>* typeset	
Additional data availability information:	

Manuscript
Click here to access/download;Manuscript;Manuscript.docx

Mung bean seed classification based on multimodal features and Kepler-optimized stacking ensemble learning model

Shaozhong Song[1,2], Fengwei Leng[1], Ming Fang[1], Xiaofeng An[2*], Yaxin Cai[3]

[1] School of Artificial Intelligence, Changchun University of Science and Technology, Changchun, China

[2] School of Data Science and Artificial Intelligence, Jilin Engineering Normal University, Changchun, China

[3] School of Computer Science and Technology, Changchun University of Science and Technology, Changchun, China

*** Correspondence author:**
E-mail: an.xf@jlenu.edu.cn(XA)

Abstract

Accurate classification of mung bean seeds is essential for enhancing both their nutritional value and crop yields. However, current methods are limited, primarily due to the time-consuming and inaccurate classification process resulting from a lack of diverse dataset features. To overcome these challenges, this study develops a multimodal dataset that integrates Raman spectral features and image-based features through early fusion. Furthermore, the classification of mung bean seed varieties is achieved in a rapid, accurate, and non-destructive manner by optimizing a stacking ensemble learning model using the Kepler Optimization Algorithm (KOA). The multimodal dataset comprises 59 features, selected using the Competitive Adaptive Reweighted Sampling (CARS) method. Specifically, 44 key features are extracted from 700 Raman spectral data points, while 15 key features are derived from 43 image-based features. The study also used the Kepler Optimization Algorithm to optimize the parameters of various machine learning models, including Decision Tree (DT), Support Vector Machine (SVM), k-Nearest Neighbor (KNN), Backpropagation Neural Network (BPNN), Random Forest (RF), and Gradient Boosting Decision Tree (GBDT). By constructing a stacking ensemble learning model, the research effectively leverages the strengths of multiple classifiers, thereby enhancing the overall classification performance. Experimental results demonstrate that the proposed method significantly improves mung bean seed classification accuracy, with the Kepler-optimized stacking ensemble model achieving an accuracy of 90.71%. This represents a 3.24% improvement over KOA-RF and a 1.59% improvement over KOA-GBDT. In comparison to baseline models, the proposed method proves to be more efficient.This study underscores the potential of combining multimodal features with a Kepler-optimized stacking ensemble learning model for mung bean seed classification. It highlights the role of advanced artificial intelligence techniques in agricultural production and provides valuable technical support for the precise classification of mung bean seeds.

1. Introduction

Mung beans have been used as an important food in many countries and regions around the world, dating back more than 3500 years (1). Mung beans are consumed in a variety of ways, and they have a long history and widespread use in China, where they have been cultivated for more than 2000 years (2). High-quality seeds are essential for the continuation of good varieties, and they ensure crop yield and quality (3). Seed yield is a key indicator of economic productivity of mungbean, and this indicator is significantly influenced by the variety of mungbean seed (4). In addition, the variety of mungbean seed directly affects its nutrient composition, including key nutrients such as oil, protein, and sugar (5). Therefore, the classification of seed varieties of mungbean as an important food crop is of great value. Various varieties of mung bean seeds exhibit a high degree of morphological similarity. Early researchers primarily relied on the morphological traits of mung bean seeds for classification (6), a method that, while enhancing objectivity, was also labor-intensive and time-consuming. Subsequently, in order to improve the classification efficiency of mung bean seeds, researchers developed DNA fingerprint profiles through chemical methods (7). However, these methods require specialized laboratories and skilled technicians and may potentially damage the seeds. To address the limitations of traditional detection methods, modern techniques such as machine vision and Raman spectroscopy have been adopted for seed classification.

Machine vision primarily emulates the human visual system to process and analyze acquired image data (8). Key components of image information include color, texture, and geometric features (9). The advancement of machine vision technology offers precise, efficient, and non-destructive methods for sensing and data processing in plant phenotyping and precision agriculture (10). For instance, a UNet-based network model has been employed to distinguish between corn and wheat seedlings and weeds in the field (11); a lightweight MssiapNet model was developed to identify different wheat seedling varieties (12); and an improved deep residual convolutional network was applied to identify various corn seed varieties (13). However, machine vision techniques necessitate a

high degree of integrity in the appearance of the target, with even minor defects potentially compromising classification accuracy.

Raman spectroscopy, a form of scattering spectroscopy, relies on inelastic light scattering to obtain chemical information about the internal structure of a substance (14). This technique has proven to be a rapid, non-invasive, and effective tool for quality analysis (15). In recent years, Raman spectroscopy has been applied to identify plant species and their varieties, as well as to assess the nutritional content and commercial value of seeds (16). For example, Raman spectroscopy has been used to classify viable and nonviable maize kernels (17), investigate the microstructure of quinoa fruit seeds and identify the specific location of nutrients within the seeds (18), and combine spectral peak decomposition algorithms with Raman spectroscopy to analyze the oil content in maize seeds (19). These studies highlight the potential of Raman spectroscopy for mung bean seed classification. Although Raman spectroscopy excels in intrinsic quality assessment, it is less effective in appearance evaluation, necessitating further research.

The construction of multimodal datasets that integrate multiple features is gaining prominence in contemporary research. Fusing various types of features can enhance the expressiveness of the data, and by synthesizing information from different features, the limitations of individual feature types can be effectively mitigated. For example, the integration of spectral and spatial features through residual hypergraph convolutional networks has enabled efficient classification of hyperspectral images (20). Data fusion of spectral and image texture features analyzed using SVM has achieved 97.5% accuracy in identifying the year of corn seeds (3). Furthermore, combining morphological features of maize seeds with hyperspectral data has facilitated the classification of maize seed varieties using a random forest model optimized by the Improved Gray Wolf algorithm, achieving an accuracy of 95.9% (21). These findings underscore the effectiveness of constructing multimodal datasets that combine image and spectral features to enhance model classification performance.

Classification using one-dimensional data such as spectral data or image features usually relies on machine learning models (22). Traditional machine learning models include DT (23), SVM (24), KNN (25), and others. Individual machine learning models can be surpassed by integrated learning models that reduce bias and variance and better capture the underlying distribution of the data (26). By fusing multiple simple machine learning models, integrated learning models have been shown to be significantly effective in improving prediction accuracy (27). For example, an integrated learning model combining VGG11, ResNet18, and MobileNet achieved 95.79% accuracy in crop leaf disease identification (28), and RF and GBDT obtained R^2 values of 0.93 and 0.88 in peanut plot yield prediction, respectively (29) based on improved AlexNet, GoogLeNet, ResNet50, and MobileNetV3 as base learners achieved a recognition rate of 99.69% on the rice dataset (30). The performance of machine learning models is affected by model architecture, parameter tuning, and so on. Parameters have a direct effect on the performance of machine learning models, so parameter optimization is a key step in improving model performance. Meta-heuristic optimization algorithms are widely used by researchers to search and optimize the parameters of machine learning models. Meta-heuristic optimization algorithms follow the principle of exploring the search space and utilizing the available information in order to find the optimal solution (31), and the process of finding the optimal solution is based on the assessment of the quality of the solution rather than the specific form of the objective function or the constraints of the problem (32). For example, particle swarm algorithms are used to optimize BP neural networks and RF (33), improved cuckoo search algorithms are used to optimize extreme learning machines (34), and genetic algorithms and genetic dominance algorithms are used to optimize SVM (35). However, metaheuristic algorithms need to make progress in reducing the number of iterations and avoiding local optima to improve the overall performance of the algorithms.

Due to the similarity in appearance, genetic composition, and growth conditions among different varieties of mung bean seeds, accurately and efficiently identifying them presents significant

challenges. To overcome these limitations, this study introduces a novel stacking ensemble learning model for mung bean seed identification. This model leverages a multimodal dataset that combines Raman spectral data and image-based features, while optimizing the parameters of the base learners within a stacking ensemble learning framework using the KOA. The key contributions and innovations of this paper are as follows:

(1). A novel method for constructing datasets is proposed. Raman spectral features and image information features are extracted separately, and subsequently, a multimodal dataset is constructed through early fusion.

(2). A novel approach for model parameter optimization is presented. The key parameters of the base learner in the stacking ensemble learning model are optimized utilizing the KOA.

(3). A novel approach for constructing a stacked ensemble learning model has been employed. Optimized machine learning models serve as base learners, while the selection of the meta-learner model is determined using a combined optimization strategy.

(4). The experimental results confirm the efficiency and practicality of this model for mung bean seed classification, which provides technical support for combating infringement and the production and sale of fake and shoddy seeds, and helps to improve the yield of the mung bean crop and ensure its nutrient content, thus contributing significantly to agricultural production and food safety.

The remainder of the paper is organized as follows: the "Materials and methods" section provides a detailed description of the dataset construction process and the underlying methodology, the "Results and discussion" section presents a partial analysis and interpretation of the experimental results, and the "Conclusion" section offers a summary of the key findings and contributions of the paper.

2. Materials and methods

As illustrated in Fig 1, the classification process of mung bean seeds is organized into six distinct stages: 1. Screening the original mung bean seed samples; 2. Acquiring the Raman spectral features and image information features of the seeds, followed by appropriate preprocessing; 3. Extracting features from both the Raman spectra and image data; 4. Conducting early fusion of the extracted salient features to construct a multimodal dataset.; 5. Inputting the multimodal dataset into a established stacking ensemble learning model; and 6. Outputting the classification results and conducting a data analysis.

Fig 1. The flowchart for mung bean seed classification based on multimodal features and Kepler-optimized stacking ensemble learning model

2.1. Mung bean seed dataset.

The mung bean seed samples used in this experiment were provided by the Research Institute of the Jilin Academy of Agricultural Sciences. These seeds were meticulously selected and are widely distributed in the market. As shown in Fig 2, seven varieties of mung bean seeds were chosen for data collection: Jilv No. 7 (A), Jilv No. 11 (B), Jilv No. 13 (C), Jilv No. 9 (D), Baolv No. 2013 (E), Jilv No. 10 (F), and Jilv No. 5 (G). For each variety, 100 seeds were randomly selected, resulting in a total of 700 data samples.

Fig 2. Selected seven varieties of mung bean seeds

In the acquisition of Raman spectral data for mung bean seeds, this study employed a Hooke D100 near-infrared confocal Raman spectroscopy system, which was equipped with a 785 nm wavelength laser operating at 50 mW power. To capture subtle spectral variations, a precise sampling method was utilized. Raman intensities were recorded at 2cm^{-1} intervals across a Raman shift range from 400cm^{-1} to $1800\,\text{cm}^{-1}$, yielding a total of 700 distinct Raman spectral features.The Raman spectral data collection procedure is described in S1 Appendix.

In this study, mung bean seed images were captured using a Canon EOS 1500D camera, which has an effective resolution of 24.1 megapixels. To extract the necessary image features, the captured images were first converted to grayscale and then processed into binary images to facilitate effective separation of the seeds from the background. Using the binary image as a basis, the original images were accurately segmented, allowing for the extraction of individual mung bean seed images. From these segmented images, a total of 43 key features were derived, including 18 geometric features, 7 texture features, and 18 color features. The image features collection procedure is described in S2 Appendix.

2.2. Data preprocessing

We perform preprocessing operations on the acquired raw Raman spectral data and the image numerical data, respectively.

In this study, we initially apply the Savitzky-Golay (SG) filtering method to reduce noise in the Raman spectra, with the aim of minimizing its influence on the spectral analysis. The choice of window size in the SG filter directly determines the extent of smoothing, while the polynomial order plays a critical role in the filter's ability to adapt to the data's finer details. Given the complexity and richness of the Raman features, a window size of 15 is selected in this work. Moreover, to preserve peak characteristics during the smoothing process, a polynomial order of 3 is employed. Following this, the max-min normalization technique is applied to standardize the spectral data, ensuring that the data are normalized to a consistent scale for comparative analysis.

For the image information features, we apply the max-min normalization method to process the data. This approach is necessary because the collected image features encompass geometric characteristics, textural properties, and color attributes, which can exhibit significantly different value ranges. Max-min normalization ensures comparability across these diverse features.

Following the aforementioned preprocessing steps, we can ensure that all data is standardized and consistent, providing a reliable foundation for subsequent analysis and processing.

2.3. CARS feature extraction

CARS is grounded in Darwin's concept of natural selection, specifically the principle of "survival of the fittest," and enhances feature selection by mimicking the process of biological evolution (37). The principles of CARS feature extraction are illustrated in Fig 3. CARS is distinguished by a two-phase feature extraction process, comprising an initial fast extraction stage followed by a more refined extraction stage.

Fig 3. Principles of CARS feature extraction

The objective of the fast extraction phase is to efficiently eliminate a substantial number of redundant and irrelevant features. During this phase, the exponential degradation function (EDF) is primarily employed to calculate the ratio of retained features and to discard those with minimal weights. For a dataset containing p features with a sampling number N, the proportion of the number of features retained in the ith iteration is given in Eq. (1).

$$r_i = ae^{-ki} \tag{1}$$

Where a and k are constants, in order to ensure that the first sampling includes all features selected, and the Nth sampling includes only two features, the values of a and k must satisfy $r_1 = 1$ and $r_N = 2/p$. The values of a and k can be determined, see Eq. (2):

$$a = \left(\frac{m}{2}\right)^{\frac{1}{N-1}}, \quad k = \frac{ln\left(\frac{m}{2}\right)}{N-1} \tag{2}$$

Following the initial screening of the EDF, a more refined selection of features is conducted using Adaptive Reweighted Sampling (ARS). This technique involves weighted random sampling based on

the current feature weights, where variables with higher weights are more likely to be retained, while those with lower weights are progressively discarded.

2.4. Stacking ensemble learning model

The stacking ensemble learning model enhances predictive performance by utilizing the predictions of multiple base learners as input features for the meta-learner. The principles of stacking ensemble learning model is shown in Fig 4. For a stacking ensemble learning model with T base learners and one meta-learner, the input data is x, then the output Y can be expressed as Eq. (3):

$$Y = f(h_1(x), h_2(x) \ldots h_t(x)) \tag{3}$$

where $h_i(\cdot)$ represents the action of the base learner on the input and $f(\cdot)$ represents the action of the meta-learner on the input.

Fig 4. Principles of stacking ensemble learning model

To enhance the generalization performance of the model and mitigate the risk of overfitting, stacking ensemble learning typically employs cross-validation to partition the dataset, which is subsequently used for model training. Assuming a training-to-test set ratio of 4:1, with k-fold cross-validation, the model's training process is outlined as follows:

(1). Data Set Division: The dataset is partitioned into a training set and a test set, with 80% of the data allocated to the training set and 20% to the test set. Subsequently, the training set is randomly divided into k subsets of equal size.

(2). Training and prediction of base learner: Select one subset to serve as the validation set for the base learner, while the remaining $(k-1)$ subsets are used as the training set. For each base learner,

train it using the designated training set. Subsequently, apply the trained base learner to the corresponding validation set to generate prediction results.

(3). Cross-validation: In the second step, the procedure is repeated k times, with each iteration designating a different subset as the validation set. This method allows for the collection of prediction results, denoted as $P_1, P_2 \dots P_k$, from all base learners across the k iterations of cross-validation. The prediction result P_i for an individual base learner is derived from the aggregation of predictions obtained during the k cross-validation iterations.

(4). Constructing the secondary training set: Constructing the secondary training set involves aggregating the prediction results, denoted as $P_1, P_2 \dots P_k$, from the base learners along with the corresponding labels from the original training set to create the secondary training set.

(5). Training the meta-learner: The meta-learner is trained using a secondary training set. Its primary objective is to determine the most effective way to integrate the predictions from the first layer of the base learner, thereby enhancing the overall performance of the model.

Once the stacking ensemble learning model has been trained, its overall efficacy and generalization capability can be thoroughly assessed by calculating various performance metrics on an independent test set.

2.5. Kepler Optimization Algorithm

The KOA is a novel metaheuristic algorithm inspired by Kepler's three laws of planetary motion (38). The principles of Kepler Optimization Algorithm is shown in Fig 5. The algorithm employs two strategies: randomly updating planetary positions and adjusting the distances of planets from the Sun. These strategies are designed to balance the algorithm's global and local search capabilities.

Fig 5. Principles of Kepler Optimization Algorithm

In the planetary position update strategy, the KOA algorithm emulates the motion of a planet as it gradually approaches the Sun in one segment of its elliptical orbit and recedes from the Sun in another segment. The position of the planet at iteration $t+1$ is denoted by $\vec{X}_i(t+1)$, which is mathematically represented by Eq. (4).

$$\vec{X}_i(t+1) = \vec{X}_i(t) + \mathcal{F} \times \vec{V}_i(t) + \left(F_{g_i}(t) + |r|\right) \times \vec{U} \times \left(\vec{X}_S(t) - \vec{X}_i(t)\right) \tag{4}$$

$\vec{V}_i(t)$ is the velocity that moves the planet from position $\vec{X}_i(t)$ to $\vec{X}_i(t+1)$, and $F_{g_i}(t)$ is the gravitational force exerted by the Sun on the planet at position $\vec{X}_i(t)$.

In the strategy of updating the distance between the planet and the Sun, the KOA algorithm simulates the typical distance behavior that naturally changes over time between the Sun and the planet. At this point, the position $\vec{X}_i(t+1)$ can be obtained by Eq. (5).

$$\vec{X}_i(t+1) = \vec{X}_i(t) \times \vec{U}_1 + (1 - \vec{U}_1) \times \left(\frac{\vec{X}_i(t) + \vec{X}_S + \vec{X}_a(t)}{3}\right) + (1 - \vec{U}_1) \times h \times \left(\frac{\vec{X}_i(t) + \vec{X}_S + \vec{X}_a(t)}{3} - \vec{X}_b(t)\right) \tag{5}$$

In this context, the regulating parameter h plays a pivotal role. When its value is large, the exploration operation is employed to increase the distance between the planet's orbit and the Sun. Conversely, when the value is small, the Sun is positioned closer to the planets, and the exploitation operation is utilized to refine the region near the optimal solution.

3. Results and discussion

The stacking ensemble learning model adopted in this paper consists of two layers: base learners and a meta-learner. It fuses the prediction results of multiple base learners of different types and then conducts final integration through the meta-learner. By leveraging the complementarity among models, it captures the characteristics and patterns of data from multiple perspectives, effectively reducing errors and significantly improving the accuracy and generalization ability of prediction and classification. In view of this, selecting high - performing base learner models and meta-learner models is of great importance. In this paper, the KOA is first applied to optimize the parameters of six classification models,including SVM, DT, KNN, RF, MLP, and AdaBoost. The optimized models are then used as the base learners of the stacking ensemble learning model. Subsequently, the meta-learner model is selected through a combinatorial optimization approach, and finally, the stacking ensemble learning model used in this paper is constructed.

The hardware specifications employed in this experiment are as follows: the processor is an Intel Core i5-12400F with a clock speed of 2.50 GHz, the system is equipped with 16 GB of RAM, and the graphics card utilized is an NVIDIA RTX 4060 Ti.

3.1. Feature extraction and result analysis

In this study, CARS was utilized to extract features from both the Raman spectral dataset and the image feature dataset, as depicted in Fig 6. In the context of CARS-based feature extraction, the features are conceptualized as “organisms,” and the predictive performance of the model serves as an indicator of their “fitness.” The model’s prediction performance is treated as a measure of “adaptation.” During each iteration, a subset of the extracted features is utilized in the Partial Least Squares Regression model, followed by cross-validation. The performance of each feature subset is evaluated by calculating its Root Mean Square Error of Cross-Validation (RMSECV), with the subset yielding the lowest RMSECV being selected as the optimal feature subset.

Fig 6. CARS feature extraction iterative process

Fig 6.A illustrates the feature extraction process of the CARS method applied to the Raman spectral feature dataset. The process concludes after reaching the predefined maximum iteration count of 20. As depicted in Fig 6.A, the RMSECV reaches its minimum value of 1.58 at the 9th iteration. At this point, the number of selected features is 44, representing only 6.28% of the original feature set. The key features obtained during the extraction process of Raman spectral data are presented in Fig 7.A. Including 486cm^{-1}, 504cm^{-1}, 506cm^{-1}, 616cm^{-1}, 626cm^{-1}, 630cm^{-1}, 640cm^{-1}, 672cm^{-1}, 684cm^{-1}, 788cm^{-1}, 878cm^{-1}, 924cm^{-1}, 928cm^{-1}, 934cm^{-1}, 968cm^{-1}, 970cm^{-1}, 976cm^{-1}, 990cm^{-1}, 994cm^{-1}, 1012cm^{-1}, 1140cm^{-1}, 1150cm^{-1}, 1338cm^{-1}, 1342cm^{-1}, 1344cm^{-1}, 1352cm^{-1}, 1356cm^{-1}, 1444cm^{-1}, 1446cm^{-1}, 1484cm^{-1}, 1492cm^{-1}, 1500cm^{-1}, 1506cm^{-1}, 1518cm^{-1}, 1524cm^{-1}, 1590cm^{-1}, 1592cm^{-1}, 1714cm^{-1}, 1716cm^{-1}, 1722cm^{-1}, 1728cm^{-1}, 1732cm^{-1}, 1770cm^{-1}, 1772cm^{-1}.

Fig 7. Key features obtained during the CARS extraction process

Fig 6.B illustrates the feature extraction process of the CARS method applied to the image information feature dataset. Similarly, The RMSECV curve reaches its lowest point, with an RMSECV value of 1.48, at 6th iterations, where 15 features are selected. This represents 34.88% of the total number of initial features. The key features for image information extraction, as shown in Fig 7.B. Including 1. perimeter, 2. area, 3. long axis length, 4. short axis length, 22. grey scale variance product function, 25. vollath function, 26. r-mean in RGB space, 27. G-mean in RGB space, 29. R-variance in RGB space, 32. H-mean in HSV space, 34. V-mean in HSV space, 37. V-variance in HSV space, 38. L-mean in LAB space, 39. A-mean in LAB space, 42. A-variance in LAB space.

3.2. Performance evaluation of multimodal datasets

Through early fusion, we developed a multimodal feature dataset comprising a total of 59 features by combining 44 features derived from the Raman spectral dataset with 15 features extracted from the

image information dataset. This fusion process is designed to integrate information from diverse data sources to enhance model performance and improve prediction accuracy. To investigate the impact of the constructed multimodal feature dataset on classification tasks, this study employs the classical DT model in machine learning to assess the classification performance across five datasets: the Raman spectral feature dataset, the image information feature dataset, the Raman spectral subset selected using CARS, the image information subset selected using CARS, and the multimodal feature dataset.

In this study, the dataset was divided into training and test sets at a ratio of 4:1, and performance evaluation results were obtained for five distinct datasets in the task of mung bean seed classification, as illustrated in Figure 8. When comparing the classification performance of the decision tree model applied to the Raman spectral feature dataset with that of the subset of Raman spectral features extracted using CARS, we observed a 13.79% improvement in classification accuracy, while the runtime was substantially reduced to only 6.28% of the original. Similarly, when evaluating the classification performance of the decision tree model on the image information feature dataset and its subset extracted via CARS, the classification accuracy increased by 4.11%, while the runtime decreased to 41.74% of its original value. Furthermore, when the decision tree model was applied to the multimodal dataset, the highest classification accuracy of 76.43% was achieved, with minimal increase in runtime. These results suggest that the multimodal feature dataset significantly enhances classification accuracy while also improving decision-making efficiency.

Fig 8. Comparison of Classification Performance Across Various Datasets

3.3. KOA optimizes the parameters of the base learner models

To ensure that the stacking ensemble learning model effectively captures the intrinsic features of the data from multiple perspectives, thereby enhancing predictive performance, this study carefully selected a set of base learners, drawing on the choices made by previous researchers (39,40). The base learners chosen for the first layer of the ensemble model include: SVM, DT, KNN, RF, MLP and

AdaBoost. To further improve the performance of these base learners, the study applied the KOA to optimize the model's parameters. After balancing search efficiency with accuracy, the population size was set to 10, and the maximum number of iterations was limited to 50. Additionally, the model's error rate was used as the fitness function. The parameter optimization ranges and the corresponding optimal values are presented in Table 1.

Table 1. KOA optimize base learner parameters

Basic model	Parameter Optimization Range	Optimal Parameters
DT	max_depth{1,100}, max_features{0.1,1}	max_depth = 13, max_features = 0.45
SVM	c{1,1000}, gamma{0.1,1}	C = 828.92 , gamma = 0.56
KNN	n_neighbors{1,100}, p{1,5}	n_neighbors = 3, p = 2
MLP	hidden_layer_sizes(k1,k2): k1{10,100}, k2{10,100}	hidden_layer_sizes(k1,k2): k1 = 96, k2 = 74
RF	n_estimator{1,100}, max_depth{1,100}, max_features{0.1,1}	n_estimator = 92, max_depth = 78, max_features = 0.17
GBDT	n_estimator{1,100}, max_depth{1,100}, max_features{0.1,1}	n_estimator = 84, max_depth = 20， max_features = 0.46

The KOA is capable of updating the best solution in each iteration, with the elite strategy ensuring that a superior candidate solution replaces the current best solution. This iterative process continues until the termination criteria, such as reaching the maximum number of iterations, are met. At this point, the algorithm concludes, and the current best solution is considered the optimal solution, marking the completion of the optimization process. The KOA demonstrates high efficiency in parameter optimization, as illustrated in Fig 9. In the parameter optimization task, the DT model attains a fitness function value of 0.2 in the first iteration. In contrast, the SVM model requires considerably more iterations to reach a fitness function value of 0.1214 in the 25th iteration. The KNN model achieves a fitness function value of 0.1571 by the 9th iteration, MLP model reaches a value of 0.1143 by the 8th

iteration, RF model attains 0.1214 by the 9th iteration, and GBDT model reaches a value of 0.1071 by the 9th iteration. All of these models converge to the optimal parameter combinations relatively quickly, within 10 iterations. The SVM model, however, requires a higher number of iterations to identify the optimal parameter set, which may be attributed to the sensitivity of the SVM model's parameters to variations in model performance, necessitating more iterations to achieve optimal results.

Fig 9. The process of KOA optimizing base learning models

The performance metrics of the six models—KOA-DT, KOA-SVM, KOA-KNN, KOA-MLP, KOA-RF, and KOA-GBDT—optimized using the KOA algorithm are presented in Table 2. Among these, the KOA-GBDT model demonstrates the best performance, achieving an accuracy of 0.8929, which represents a 2.63% improvement over the average accuracy of 0.8700 across all six models. Additionally, its precision is 0.9008, which is approximately 3.60% higher than the average precision of 0.8695; its recall is 0.8927, showing a 2.75% increase over the average recall of 0.8688; and its F1 score is 0.8923, which is 2.35% higher than the average F1 score of 0.8718. These results underscore the effectiveness of the KOA algorithm in optimizing the parameters of machine learning models. By optimize these parameters, the model is able to more accurately capture data patterns during the learning process, thereby enhancing its predictive performance.

Table 2. Performance of models following KOA optimization

Optimized model	Accuracy(%)	Precision(%)	Recall(%)	F1-Score(%)
KOA-DT	0.8	0.7997	0.8176	0.8024
KOA-SVM	0.8786	0.8878	0.8818	0.8800
KOA-KNN	0.8429	0.8478	0.8510	0.8408
KOA-MLP	0.8857	0.8956	0.8894	0.8886
KOA-RF	0.8786	0.8860	0.8794	0.8766
KOA-GBDT	0.8929	0.9008	0.8927	0.8923

3.4. Stacking ensemble learning model construction and analysis

In constructing the stacking ensemble learning model, after optimizing the first layer of base learners, we employ combinatorial optimization to select the appropriate meta-learner. Experiments are conducted using DT, SVM, KNN, MLP, RF and GBDT as meta-learners. The resulting models are referred to as DT-stacking, SVM-stacking, KNN-stacking, MLP-stacking, RF-stacking, and GBDT-stacking, respectively. This approach aims to evaluate which meta-learner combination maximizes the classification performance of the model.

Table 3 demonstrates the classification performance of the six final stacking ensemble learning models constructed by this method. Among these models, the RF-stacking model has the best classification performance with 90.71% accuracy, 91.45% precision, 90.74% recall and 90.77% F1-Score. Compared to the KNN-stacking model, which has the second highest classification performance, RF-stacking is 3.24% higher in accuracy, 2.81% higher in precision, 2.60% higher in recall, and 3.02% higher in F1-Score.

Table 3. Performance of six stacking ensemble learning models

Stacking model	Accuracy(%)	Precision(%)	Recall	F1-Score
DT-stacking	0.8	0.7929	0.7921	0.7880
SVM-stacking	0.8714	0.8866	0.8772	0.8763
KNN-stacking	0.8786	0.8895	0.8844	0.8811
MLP-stacking	0.8714	0.8868	0.8732	0.8750
**RF-stacking**	**0.9071**	**0.9145**	**0.9074**	**0.9077**
GBDT-stacking	0.8714	0.8753	0.8707	0.8699

Fig 10 illustrates the confusion matrices of six stacking ensemble learning models, highlighting their performance in classifying mung bean seeds. Among these models, DT-stacking exhibits poor performance in classifying Baolv No. 2013 and Jilv No. 7. SVM-stacking struggles to differentiate between Jilv No. 9 and Jilv No. 5, while KNN-stacking shows weak identification accuracy for Jilv

No. 5. MLP-stacking faces challenges in classifying Jilv No. 9, and GBDT-stacking also fails to effectively classify Baolv No. 2013. The classification of Jilv No. 9, Baolv No. 2013, and Jilv No. 5 proves particularly difficult across the models, with RF-stacking demonstrating the best overall classification performance.

Fig 10. Confusion matrix for six stacking ensemble learning models

Fig 11 illustrates the Receiver Operating Characteristic (ROC) curves for six stacking ensemble learning models, which are employed to evaluate the performance of the classifiers. The ROC curves are quantified by the Area Under the Curve (AUC), with higher AUC values indicating superior performance. In the context of multi-class classification, ROC curves are generated for each individual class to assess the model's ability to differentiate between them. The figure reveals that the DT-stacking model struggles to distinguish between Baolv No. 2013 and Jilv No. 7, while the Random Forest RF-stacking model demonstrates robust performance across all categories, achieving an average AUC value of 0.9886. Based on these results, we have selected RF model as the meta-learner in the stacking ensemble to optimize classification performance.

Fig 11. ROC curves for six stacking ensemble learning models

As shown in Fig 12, the RF-stacking model demonstrates a substantial performance advantage over the two classical integrated learning model, KOA-RF and KOA-GBDT, across all four key performance metrics: accuracy, precision, recall, and F1-Score. Specifically, improvements of 3.24%, 3.22%, 3.18%, and 3.55% for accuracy, precision, recall, and F1-Score, respectively, are observed for the RF-stacking model compared to KOA-RF, and improvements of 1.59%, 1.52%, 1.65%, and 1.73% compared to KOA-GBDT. These results underscore the superior integrated classification performance of the RF-stacking model, making it the most effective model among those evaluated.

Fig 12. Comparison of classification performance of different ensemble learning models

4. Conclusion

This study introduces, for the first time, the stacking ensemble learning model based on multimodal features and the KOA, demonstrating strong performance in the classification of mung bean seeds. The experimental procedure and results are as follows: Initially, 44 key features are extracted from 700 Raman spectral features using the CARS feature extraction method, and 15 key features are extracted from 43 numerical image features. These 59 combined features form a multimodal dataset, which integrates information from multiple data sources while eliminating redundant features, thereby enhancing classification efficiency and accuracy. Subsequently, the KOA is employed to effectively search for optimal parameters for the base learner model, further improving classification performance. Finally, the optimized base learner model is used to construct a stacking ensemble learning model, and combinatorial optimization is applied to select the meta-learner model. This approach not only enhances accuracy but also mitigates the risk of overfitting and increases model flexibility.

The experimental results demonstrate that the RF-stacking ensemble learning model, constructed using KOA-DT, KOA-SVM, KOA-KNN, KOA-MLP, KOA-RF, and KOA-GBDT as base learners, with RF as the meta-learner, outperforms other baseline models in terms of accuracy, precision, recall, F1-score, and average AUC values, which are 0.9071, 0.9145, 0.9074, 0.9077, and 0.9886,

The experimental results confirmed the effectiveness and practicality of the proposed method, offering a novel technical approach for the rapid, accurate, and non-destructive classification of mung bean seeds. These findings are not only significant for mung bean seed classification but also serve as a reference for the rapid classification and quality assessment of other agricultural products through transfer learning. Moving forward, further investigation will focus on more efficient feature selection and model optimization techniques to enhance the performance of the classification model and broaden its applications in agriculture and other sectors.

Reference

1. Ganesan K, Xu B. A critical review on phytochemical profile and health promoting effects of mung bean (Vigna radiata). Food Science and Human Wellness. (2018) 7(1):11–33. doi: 10.1016/j.fshw.2017.11.002
2. Du M, Xie J, Gong B, Xu X, Tang W, Li X, et al. Extraction, physicochemical characteristics and functional properties of Mung bean protein. Food Hydrocolloids. (2018) 76:131 – 40. doi: 10.1016/j.foodhyd.2017.01.003
3. Wu N, Liu F, Meng F, Li M, Zhang C, He Y. Rapid and Accurate Varieties Classification of Different Crop Seeds Under Sample-Limited Condition Based on Hyperspectral Imaging and Deep Transfer Learning. Frontiers in Bioengineering and Biotechnology (2021) 9:696292. doi: 10.3389/fbioe.2021.696292
4. Pathak N, Singh MN, Mishra MK, Saroj SK. Nature of gene action for yield and its components in mungbean (Vigna radiata). The Indian Journal of Agricultural Sciences. (2015) 85(4):504–8. doi: 10.56093/ijas.v85i4.47915
5. Bhardwaj HL, Hamama AA. Cultivar, Planting Date, and Row Spacing Effects on Mungbean Seed Composition. Journal of Agricultural Science. (2016) 8(10):26. doi: 10.5539/jas.v8n10p26
6. Benlioglu B, Ozkan U. Multivariate Analysis and its Application for Screening Mungbean [Vigna radiata (L.) Wilczek] Landraces. Legume Research. (2022). doi: 10.18805/LRF-661
7. Zhao Q, Chen Z, Khalifa MAS, Yin F, Qu X, Zhang J, et al. DNA Fingerprinting Construction and Genetic Diversity Analysis for Azuki Bean (Vigna angularis) and Mung Bean (Vigna radiata L.) Legume Research. (2022). doi: 10.18805/LRF-652
8. Xiao Z, Wang J, Han L, Guo S, Cui Q. Application of Machine Vision System in Food Detection. Frontiers in Nutrition. (2022) 9:888245. doi: 10.3389/fnut.2022.888245
9. Chen J, Wang Y, Luo L, Yu JG, Ma J. Image retrieval based on image-to-class similarity. Pattern Recognition Letters. (2016) 83:379–87. doi: 10.1016/j.patrec.2016.01.017
10. Liu H, Xu Z. Editorial: Machine vision and machine learning for plant phenotyping and precision agriculture. Frontiers in Plant Science. (2023) 14:1331918. doi: 10.3389/fpls.2023.1331918
11. Guo X, Ge Y, Liu F, Yang J. Identification of maize and wheat seedlings and weeds based on deep learning. Frontiers in Earth Science. (2023) 11:1146558. doi: 10.3389/feart.2023.1146558
12. Feng Y, Liu C, Han J, Lu Q, Xing X. Identification of wheat seedling varieties based on MssiapNet. Frontiers in Plant Science. (2024) 14:1335194. doi: 10.3389/fpls.2023.1335194
13. Li J, Xu F, Song S, Qi J. A maize seed variety identification method based on improving deep residual convolutional network. Frontiers in Plant Science. (2024) 15:1382715. doi: 10.3389/fpls.2024.1382715
14. Ember KJI, Hoeve MA, McAughtrie SL, Bergholt MS, Dwyer BJ, Stevens MM, et al. Raman spectroscopy and regenerative medicine: a review. npj Regenerative Medicine. (2017) 2(1):12. doi: 10.1038/s41536-017-0014-3
15. Wu L, Tang X, Wu T, Zeng W, Zhu X, Hu B, et al. A review on current progress of Raman-based techniques in food safety: From normal Raman spectroscopy to SESORS. Food Research International. (2023) 169:112944. doi: 10.1016/j.foodres.2023.112944
16. Farber C, Kurouski D. Raman Spectroscopy and Machine Learning for Agricultural Applications: Chemometric Assessment of Spectroscopic Signatures of Plants as the Essential Step Toward Digital Farming. Frontiers in Plant Science. (2022) 13:887511. doi: 10.3389/fpls.2022.887511
17. Ambrose A, Lohumi S, Lee WH, Cho BK. Comparative nondestructive measurement of corn seed viability using Fourier transform near-infrared (FT-NIR) and Raman spectroscopy. Sensors and Actuators B: Chemical. (2016) 224:500–6. doi: 10.1016/j.snb.2015.10.082
18. Czekus B, Pećinar I, Petrović I, Paunović N, Savić S, Jovanović Z, et al. Raman and Fourier transform infrared spectroscopy application to the Puno and Titicaca cvs. of quinoa seed microstructure and perisperm characterization. Journal of Cereal Science. (2019) 87:25–30. doi: 10.1016/j.jcs.2019.02.011
19. Jin Y, Tian H, Gao Z, Yang G, Dong D. Oil content analysis of corn seeds using a hand-held Raman spectrometer and spectral peak decomposition algorithm. Frontiers in Plant Science. (2023) 14:1174747. doi: 10.3389/fpls.2023.1174747
20. Zhu T, Liu Q, Zhang L. Hyperspectral Image Classification Using Multi-feature Fusion Residual Hypergraph Convolution Network. In: 2023 8th International Conference on Signal and Image Processing (ICSIP). Wuxi: IEEE; (2023) p. 343–9. doi: 10.1109/ICSIP57908.2023.10271030
21. Bi C, Zhang S, Chen H, Bi X, Liu J, Xie H, et al. Non-Destructive Classification of Maize Seeds Based on RGB and Hyperspectral Data with Improved Grey Wolf Optimization Algorithms. Agronomy. (2024) 14(4):645. doi: 10.3390/agronomy14040645
22. Lussier F, Thibault V, Charron B, Wallace GQ, Masson JF. Deep learning and artificial intelligence methods for Raman and surface-enhanced Raman scattering. TrAC Trends in Analytical Chemistry. (2020) 124:115796. doi: 10.1016/j.trac.2019.115796
23. Quinlan JR. Induction of decision trees. Machine Learning. (1986) 1(1):81–106. doi: 10.1007/BF00116251
24. Cortes C, Vapnik V. Support-vector networks. Machine Learning. (1995) 20(3):273–97. doi: 10.1007/BF00994018

25. Cover T, Hart P. Nearest neighbor pattern classification. IEEE Transactions on Information Theory. (1967) 13(1):21–7. doi: 10.1109/TIT.1967.1053964
26. Shahhosseini M, Hu G, Archontoulis SV. Forecasting Corn Yield With Machine Learning Ensembles. Frontiers in Plant Science. (2020) 11:1120. doi: 10.3389/fpls.2020.01120
27. Webb GI, Zheng Z. Multistrategy ensemble learning: reducing error by combining ensemble learning techniques. IEEE Transactions on Knowledge and Data Engineering. (2004) 16(8):980–91. doi: 10.1109/TKDE.2004.29
28. He Y, Zhang G, Gao Q. A novel ensemble learning method for crop leaf disease recognition. Frontiers in Plant Science. (2024) 14:1280671. doi: 10.3389/fpls.2023.1280671
29. Pugh NA, Young A, Ojha M, Emendack Y, Sanchez J, Xin Z, et al. Yield prediction in a peanut breeding program using remote sensing data and machine learning algorithms. Frontiers in Plant Science. (2024) 15:1339864. doi: 10.3389/fpls.2024.1339864
30. Yang L, Yu X, Zhang S, Zhang H, Xu S, Long H, et al. Stacking-based and improved convolutional neural network: a new approach in rice leaf disease identification. Frontiers in Plant Science. (2023) 14:1165940. doi: 10.3389/fpls.2023.1165940
31. R N, Sheela K G. Metaheuristic Algorithm Based Maximum Power Point Tracking Technique Combined With One Cycle Control For Solar Photovoltaic Water Pumping Systems. Frontiers in Energy Research. (2022) 10:902443. doi: 10.3389/fenrg.2022.902443
32. Xia D, Quan W, Wu T. Optimizing functional near-infrared spectroscopy (fNIRS) channels for schizophrenic identification during a verbal fluency task using metaheuristic algorithms. Frontiers in Psychiatry. (2022) 13:939411. doi: 10.3389/fpsyt.2022.939411
33. Qiao J, Wang C, Su G, Liang X, Dong S, Jiang Y, et al. Application research on the prediction of tar yield of deep coal seam mining areas based on PSO-BPNN machine learning algorithm. Frontiers in Earth Science. (2023) 11:1227154. doi: 10.3389/feart.2023.1227154
34. Zhu Z, Guan S. Prediction of the Height of Fractured Water-Conducting Zone Based on the Improved Cuckoo Search Algorithm–Extreme Learning Machine Model. Frontiers in Earth Science. (2022) 10:860507. doi: 10.3389/feart.2022.860507
35. Xia D, Quan W, Wu T. Optimizing functional near-infrared spectroscopy (fNIRS) channels for schizophrenic identification during a verbal fluency task using metaheuristic algorithms. Frontiers in Psychiatry. (2022) 13:939411. doi: 10.3389/fpsyt.2022.939411
36. Savitzky Abraham, Golay MJE. Smoothing and Differentiation of Data by Simplified Least Squares Procedures. Analytical Chemistry. (1964) 36(8):1627–39. doi: 10.1021/ac60214a047
37. Li H, Liang Y, Xu Q, Cao D. Key wavelengths screening using competitive adaptive reweighted sampling method for multivariate calibration. Analytica Chimica Acta. (2009) 648(1): 77–84. doi: 10.1016/j.aca.2009.06.046
38. Abdel-Basset M, Mohamed R, Azeem SAA, Jameel M, Abouhawwash M. Kepler optimization algorithm: A new metaheuristic algorithm inspired by Kepler's laws of planetary motion. Knowledge-Based Systems. (2023) 268:110454. doi: 10.1016/j.knosys.2023.110454
39. Xu C, Ding J, Qiao Y, Zhang L. Tomato disease and pest diagnosis method based on the Stacking of prescription data. Computers and Electronics in Agriculture. (2022) 197:106997. doi: 10.1016/j.compag.2022.106997
40. Jiang H, Zhang S, Yang Z, Zhao L, Zhou Y, Zhou D. Quality classification of stored wheat based on evidence reasoning rule and stacking ensemble learning. Computers and Electronics in Agriculture. (2023) 214:108339. doi: 10.1016/j.compag.2023.108339

Supporting information

S1 Appendix. Raman spectral data collection procedure

S2 Appendix. Image features collection procedure

Click here to access/download;Figure;Fig 1.tif

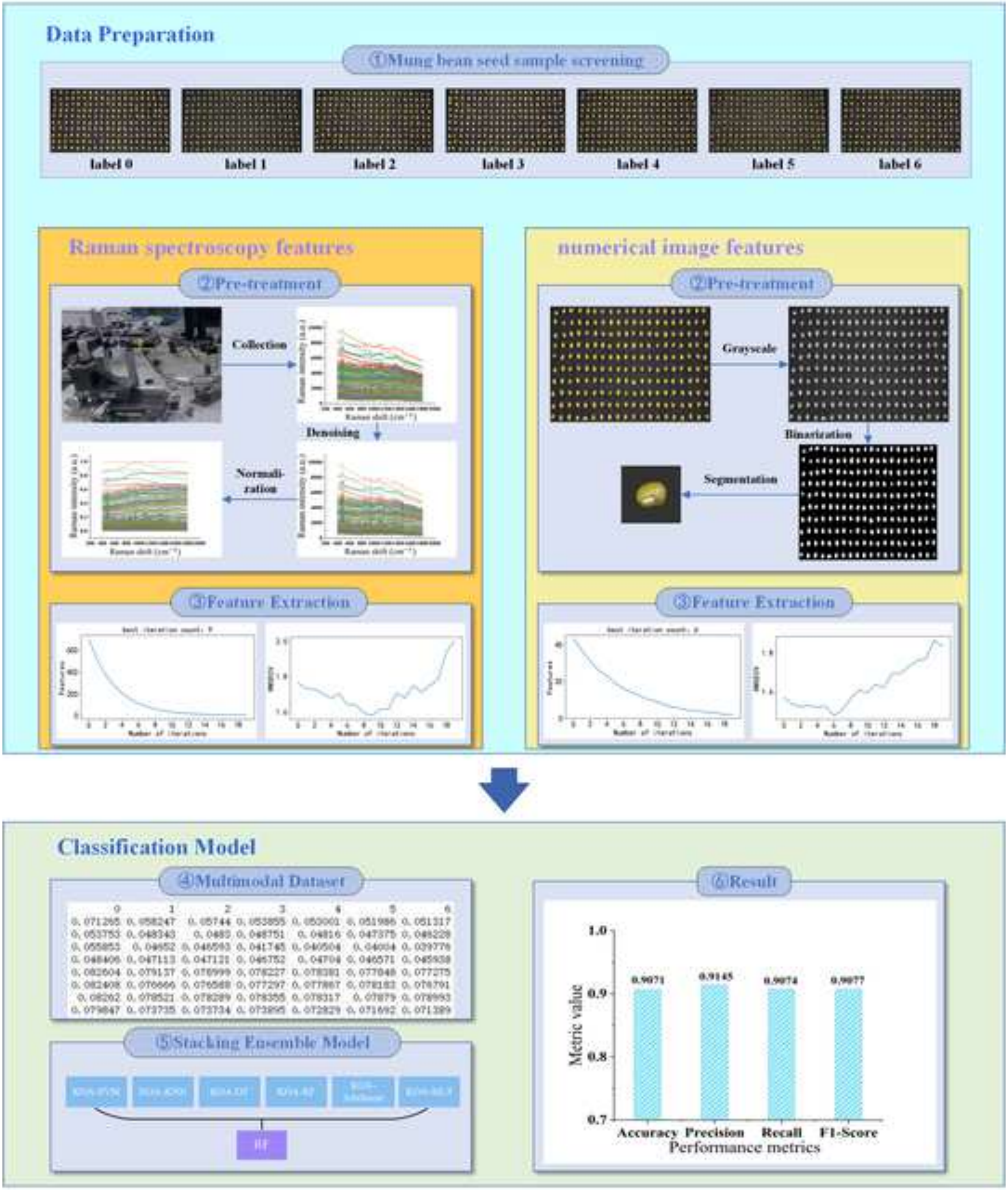

Fig 2

Click here to access/download;Figure;Fig 2.tif

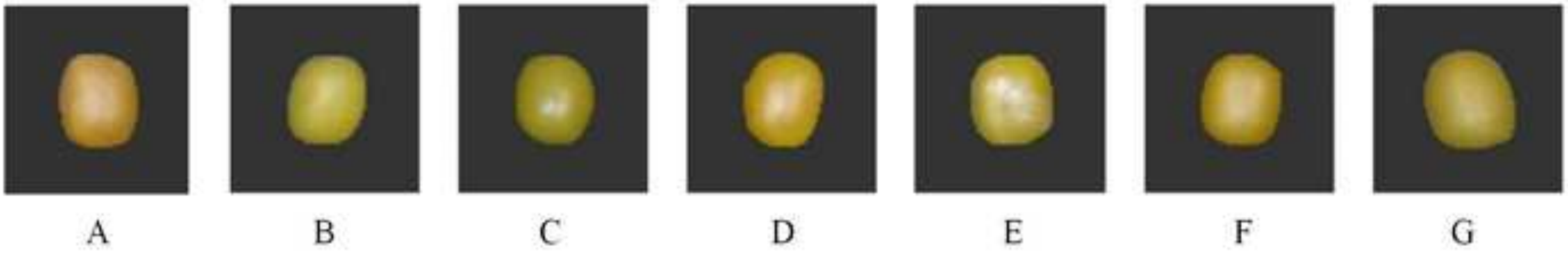

Fig 3

Click here to access/download;Figure;Fig 3.tif

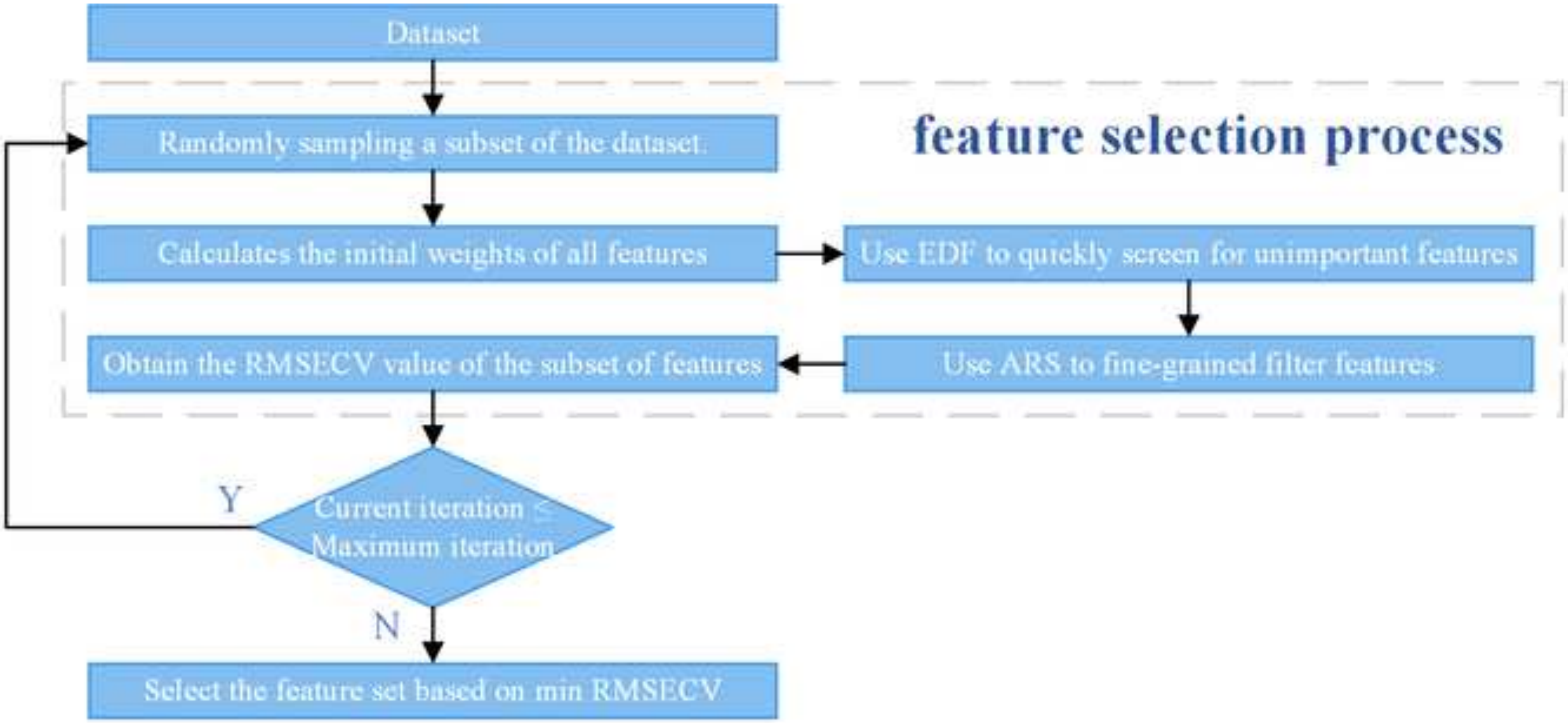

Fig 4

Click here to access/download;Figure;Fig 4.tif

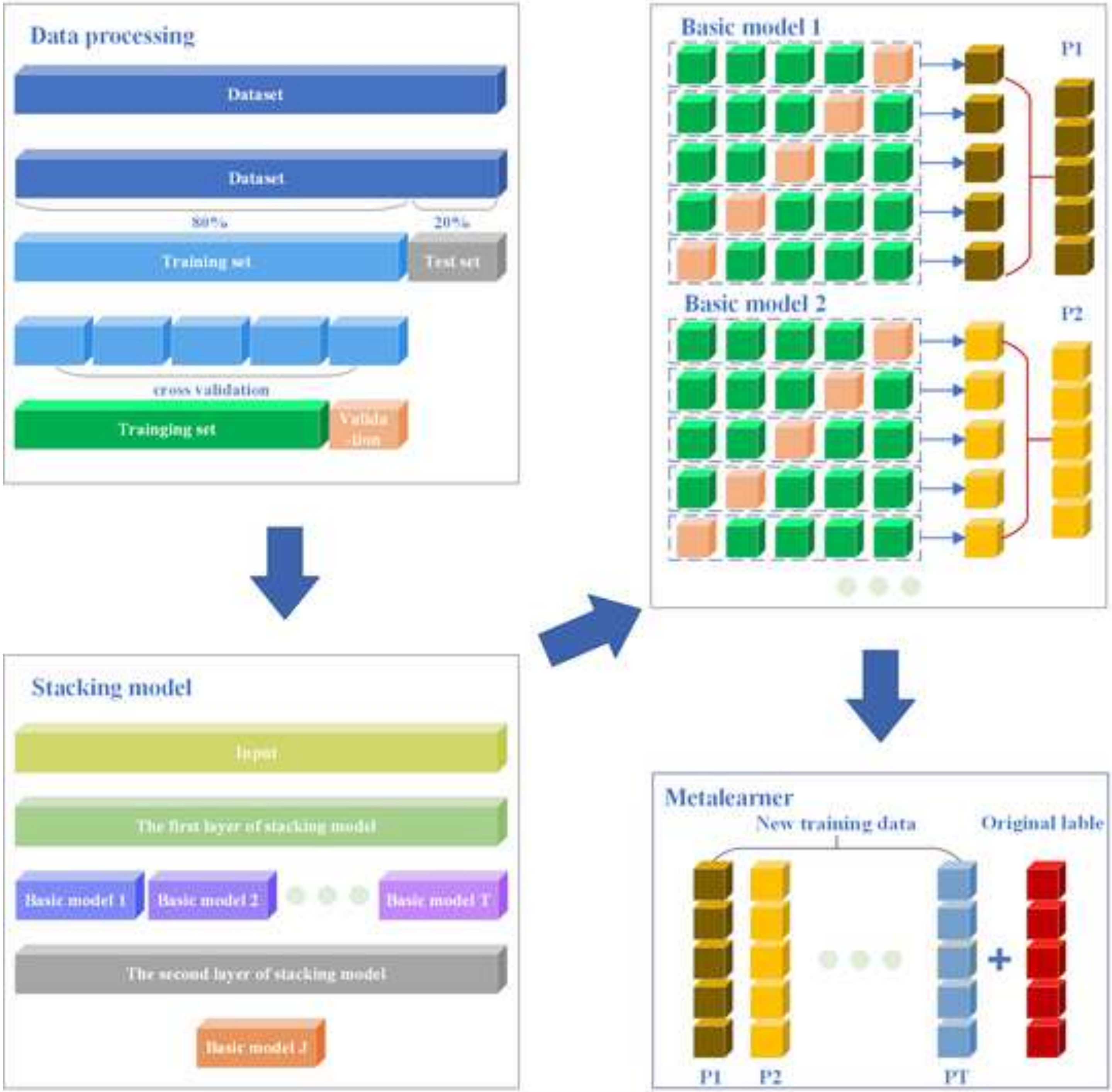

Click here to access/download;Figure;Fig 5.tif

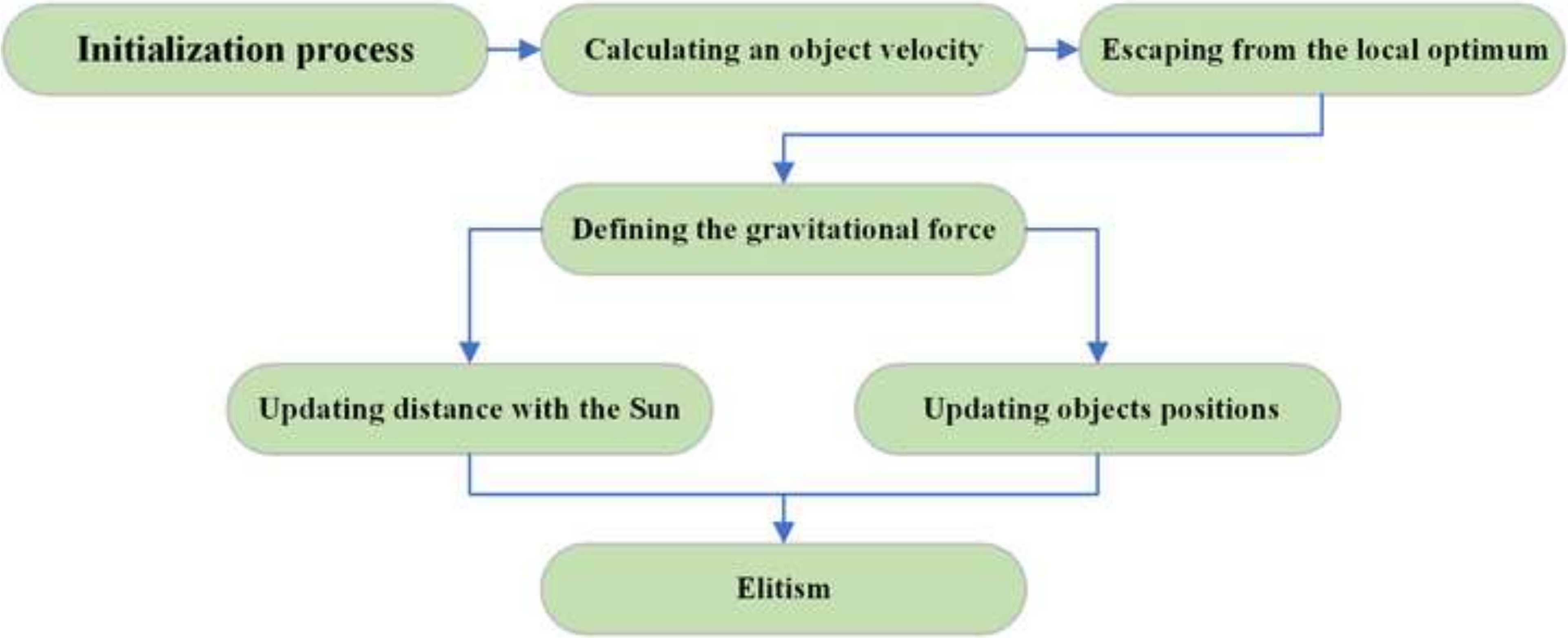

Fig 6.A

Click here to access/download;Figure;Fig 6.A.tif

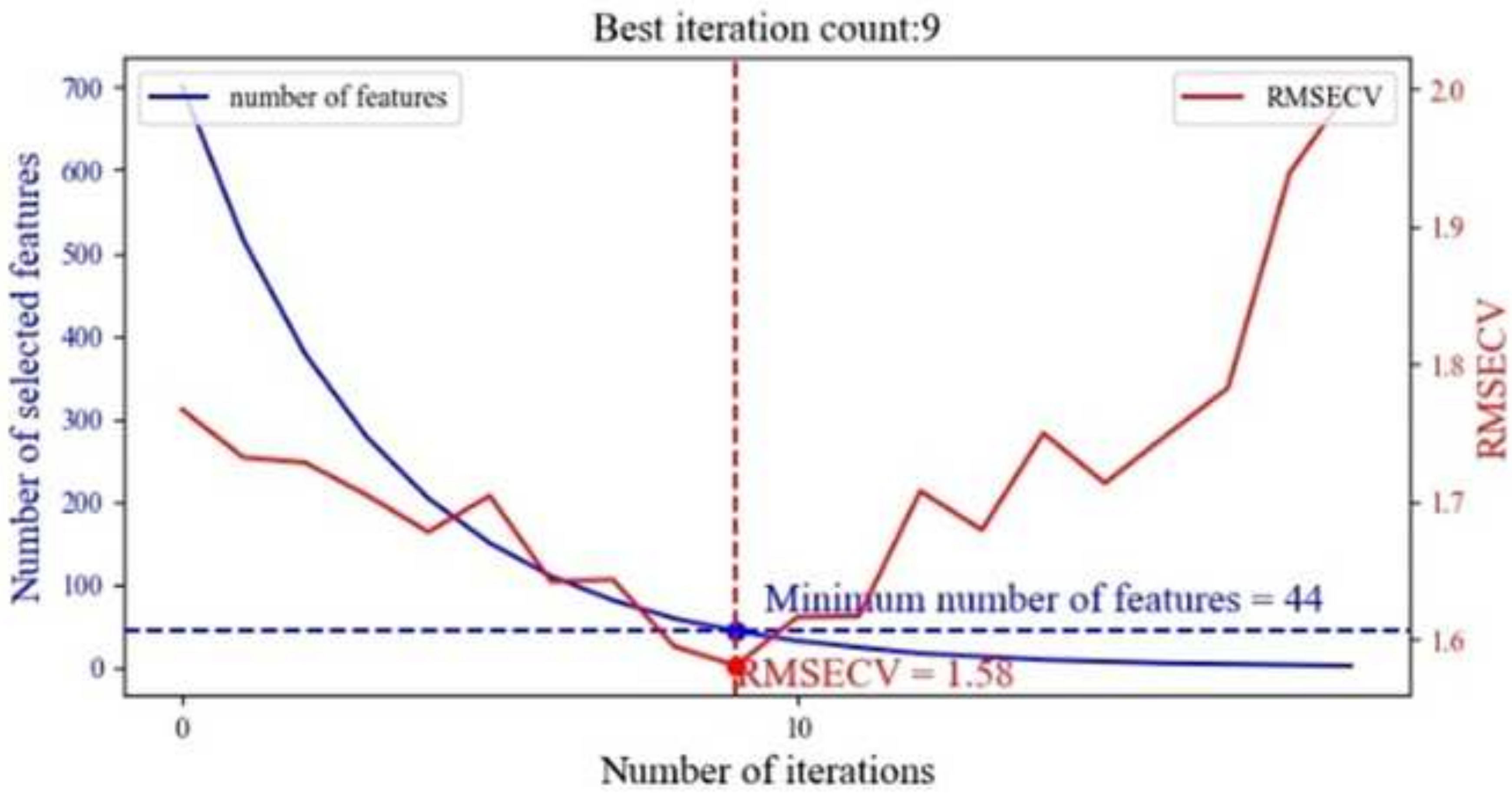

A CARS Raman spectroscopy process

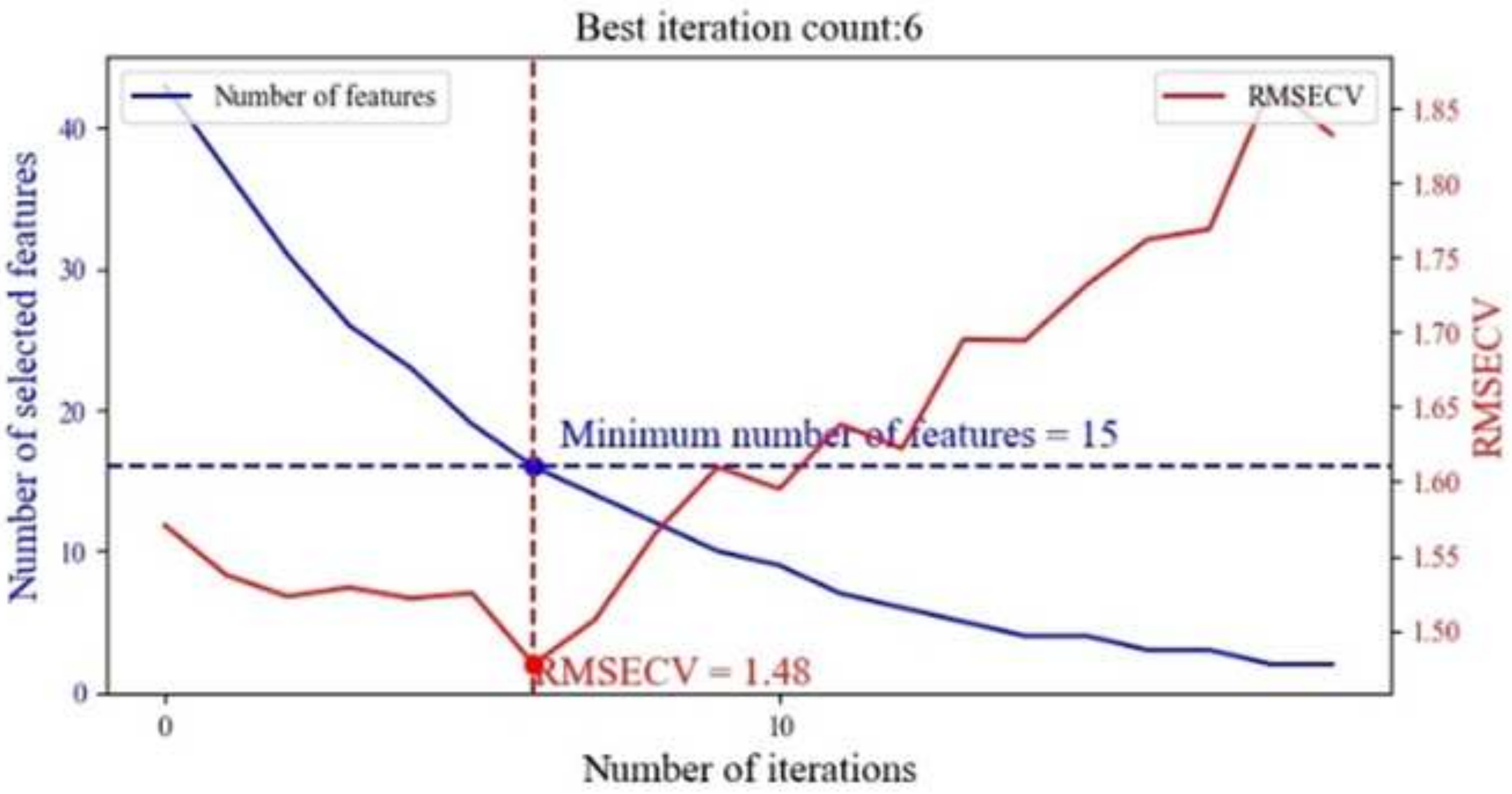

B CARS Image Information Process

Fig 7.A

Click here to access/download;Figure;Fig 7.A.tif

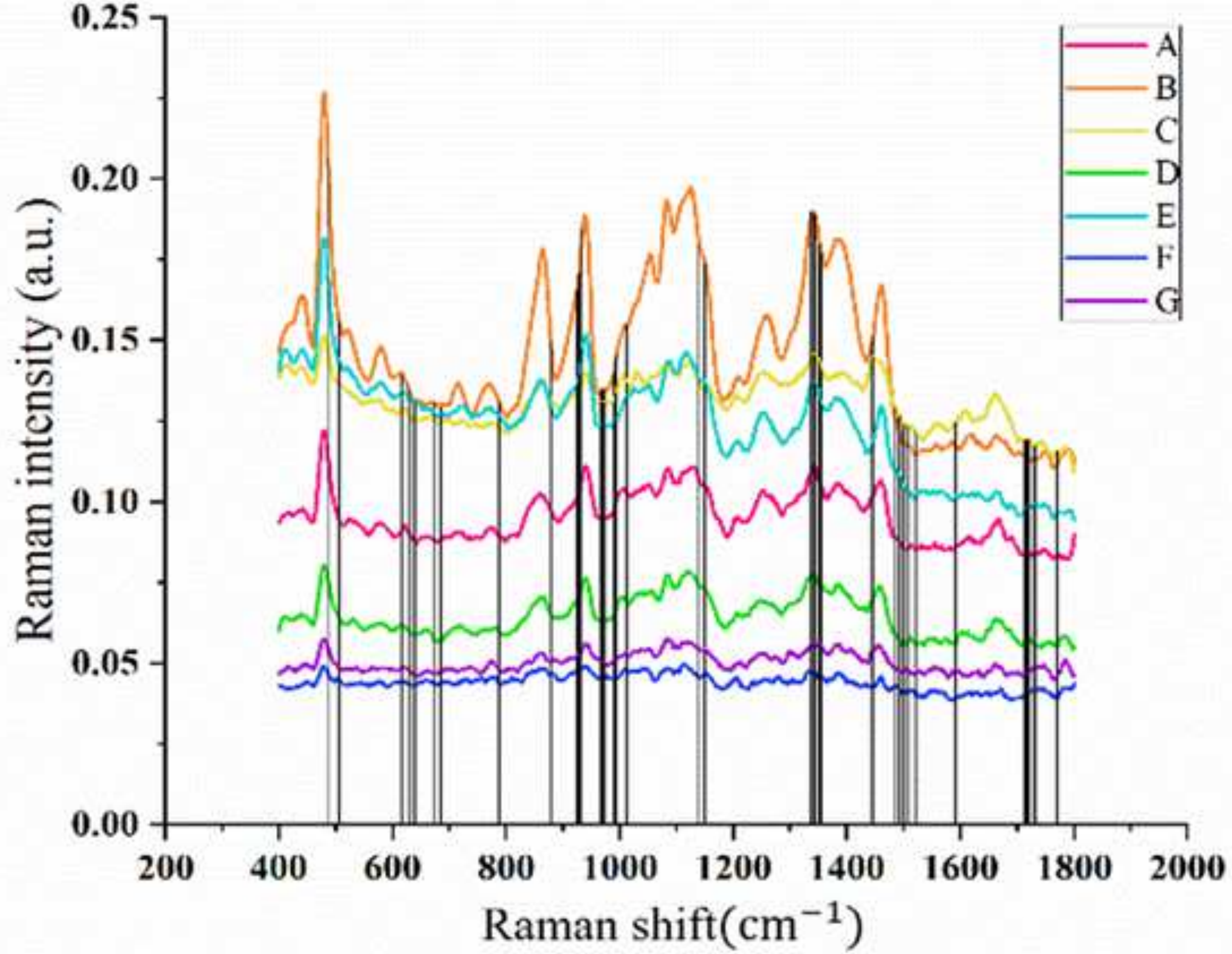

A CARS Raman spectroscopy results

Fig 7.B

Click here to access/download;Figure;Fig 7.B.tif

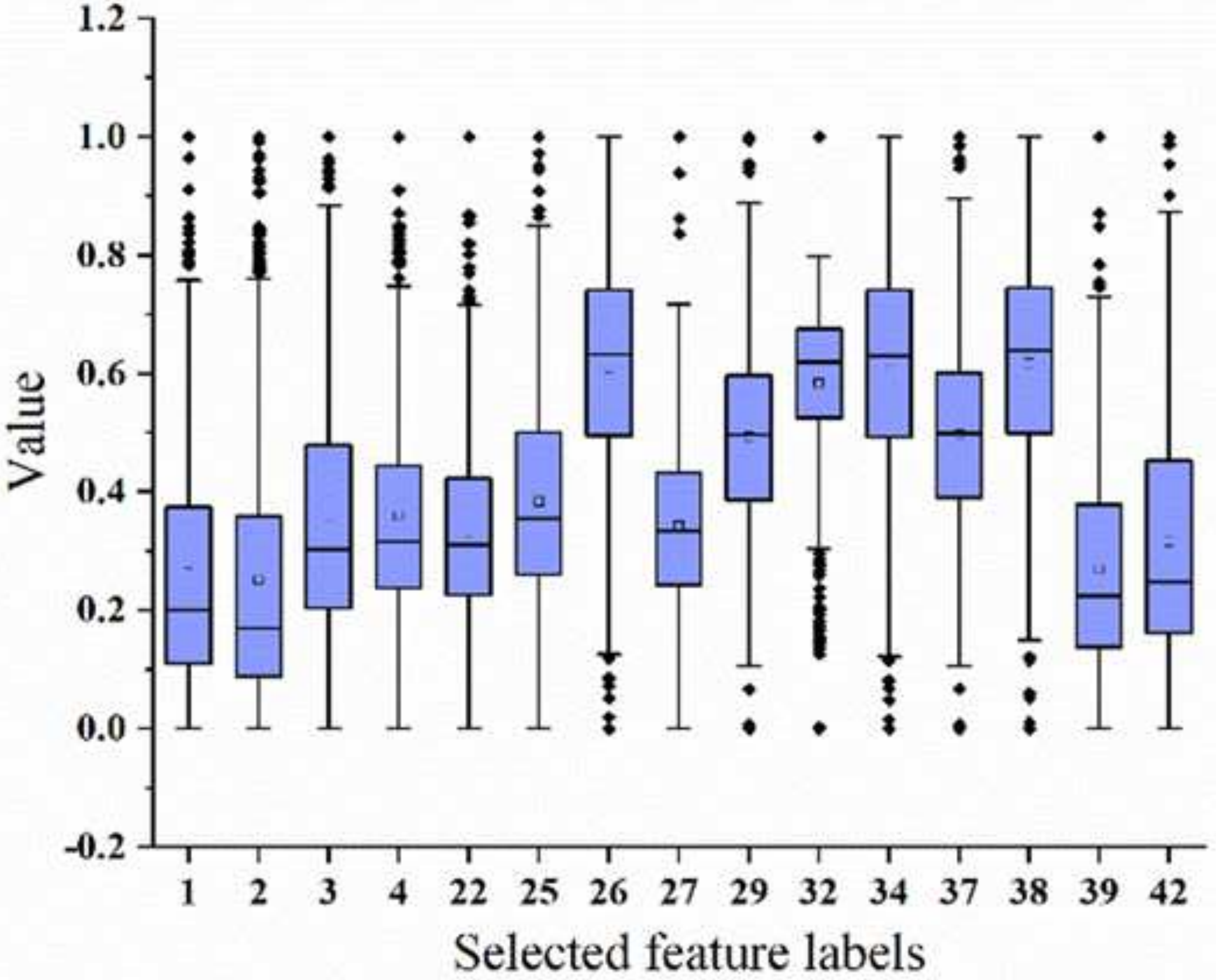

B CARS Image Information results

Fig 8

Click here to access/download;Figure;Fig 8.tif

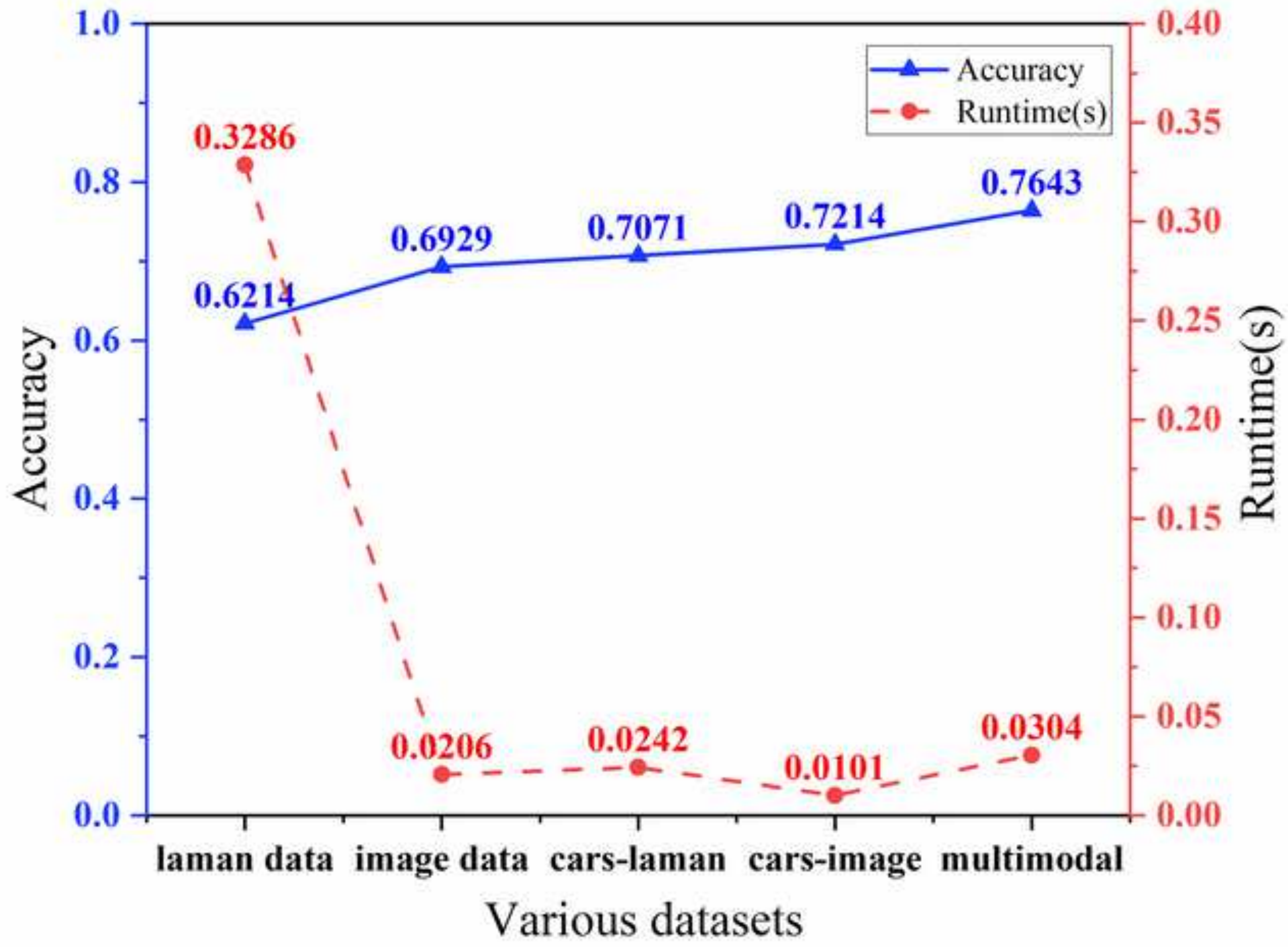

Click here to access/download;Figure;Fig 9.A.tif

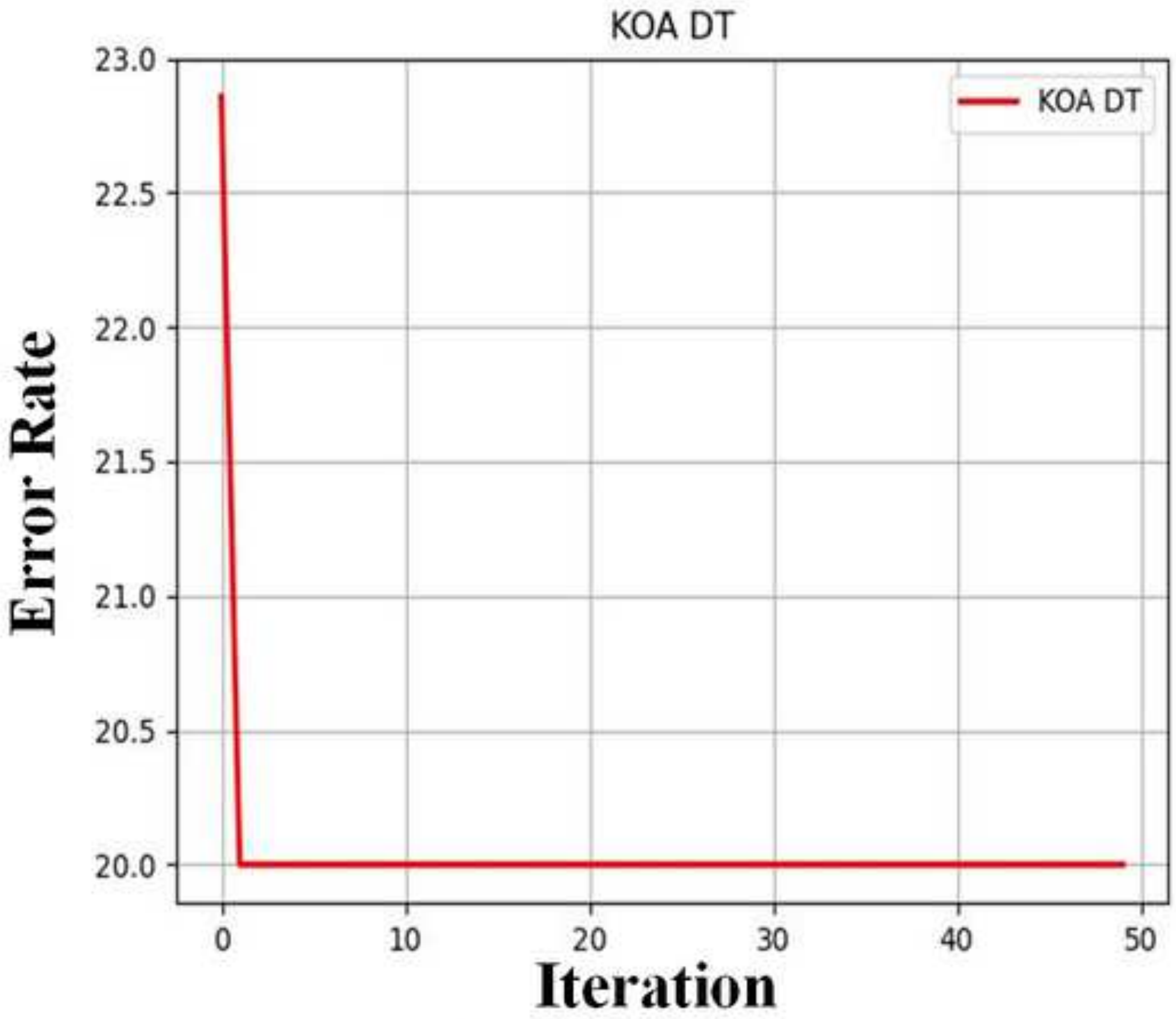

A. KOA-DT

 Click here to access/download;Figure;Fig 9.B.tif

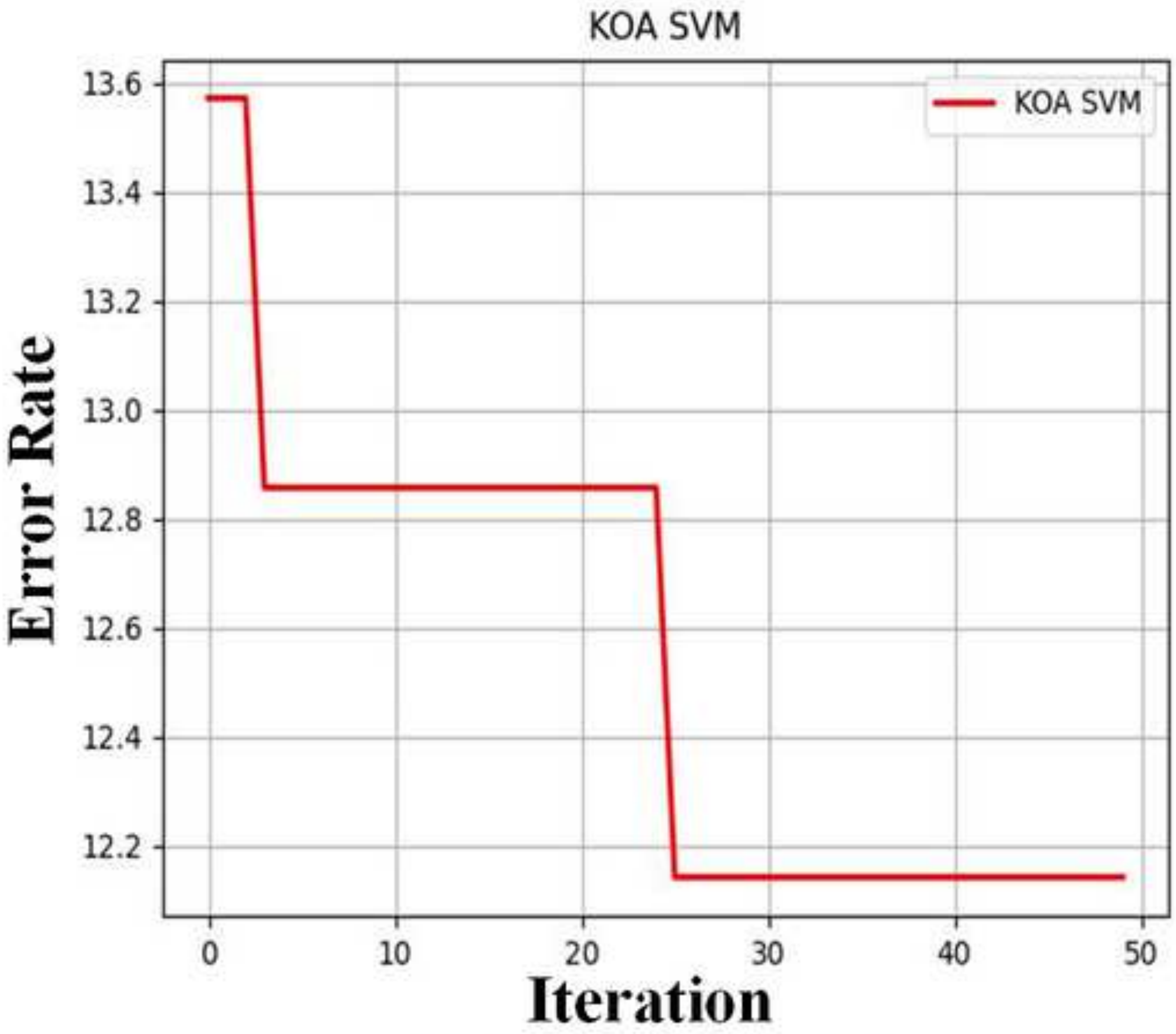

B. KOA-SVM

Fig 9.C

Click here to access/download;Figure;Fig 9.C.tif

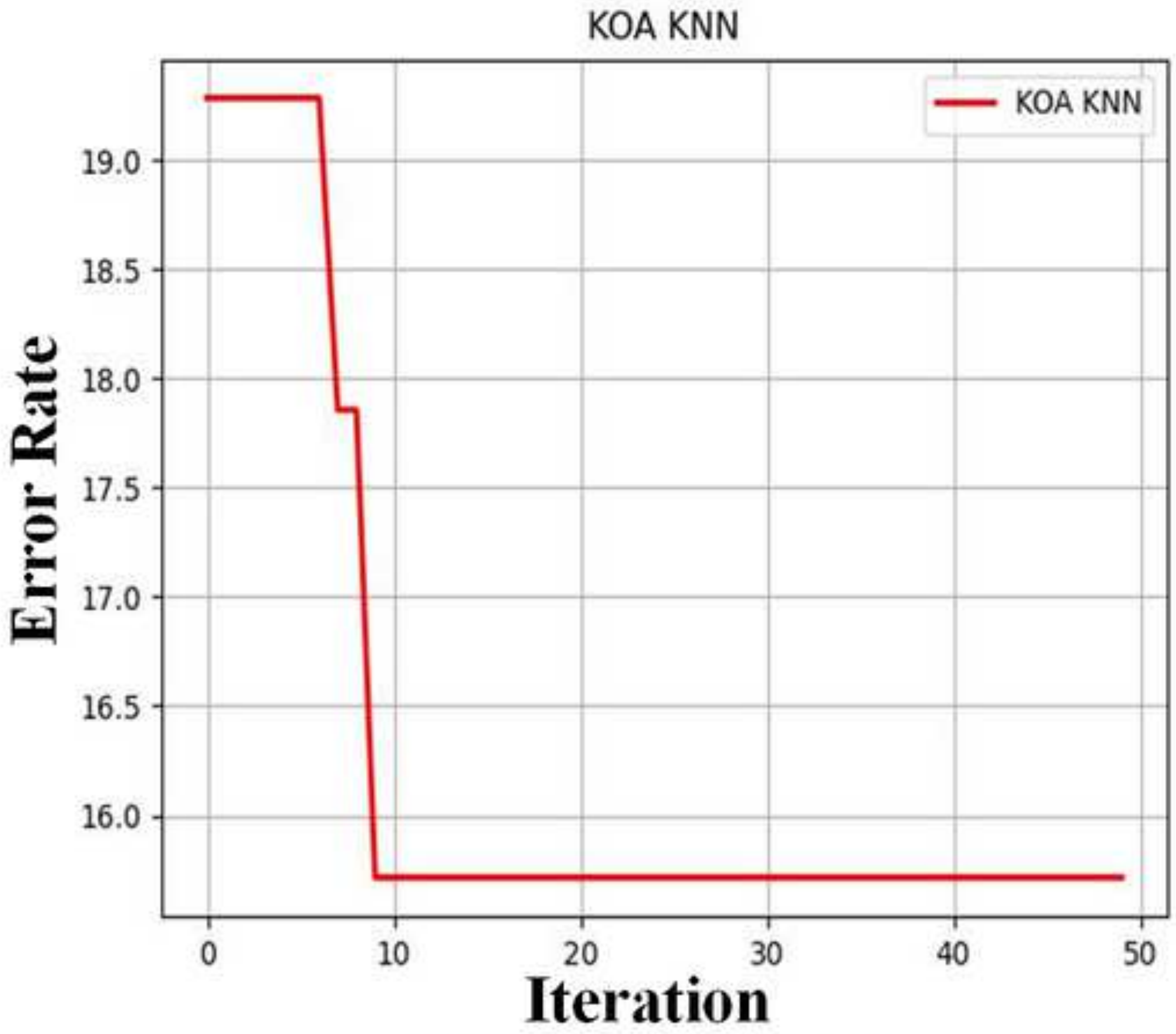

C. KOA-KNN

 Click here to access/download;Figure;Fig 9.D.tif

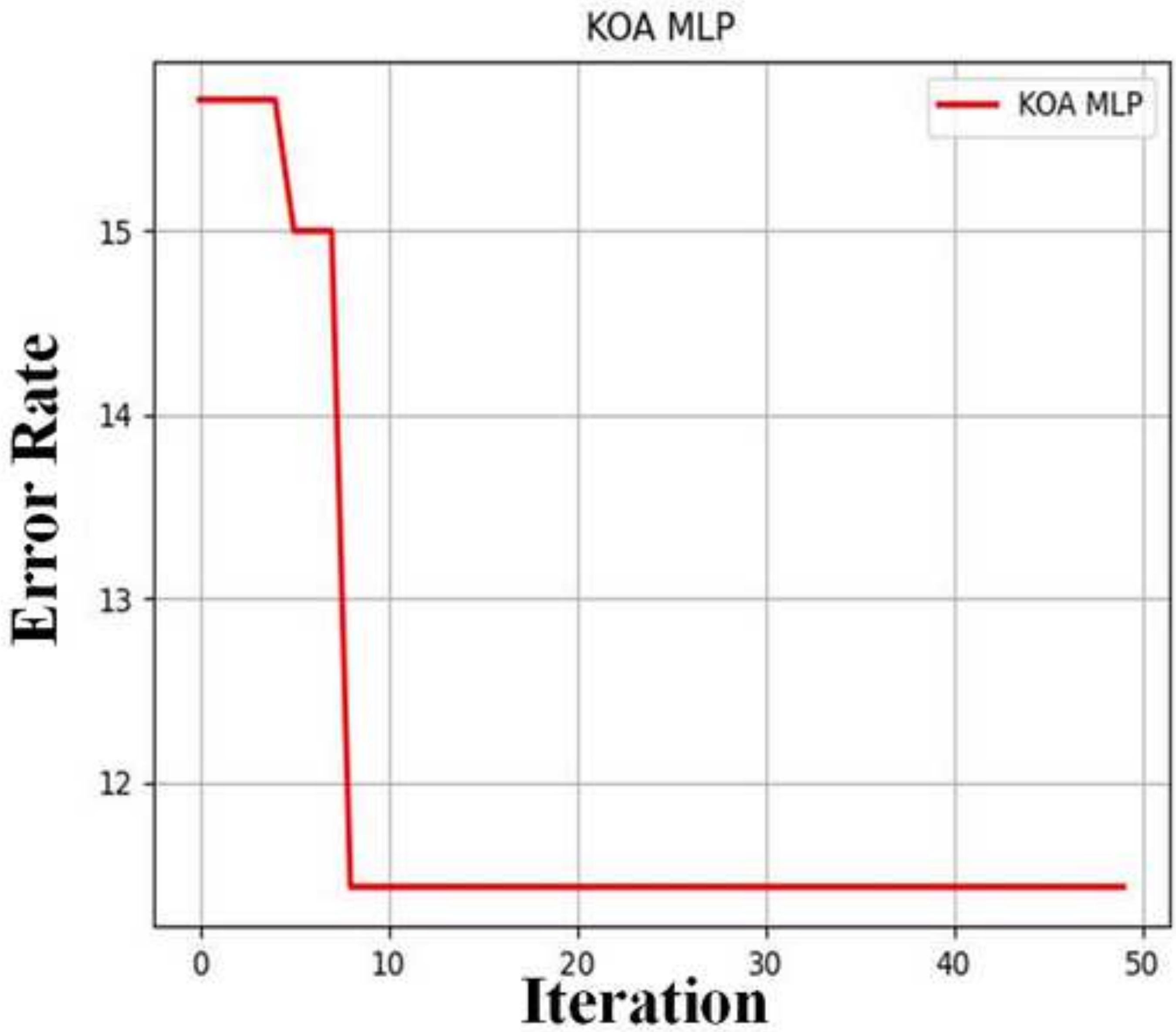

D. KOA-MLP

Click here to access/download;Figure;Fig 9.E.tif

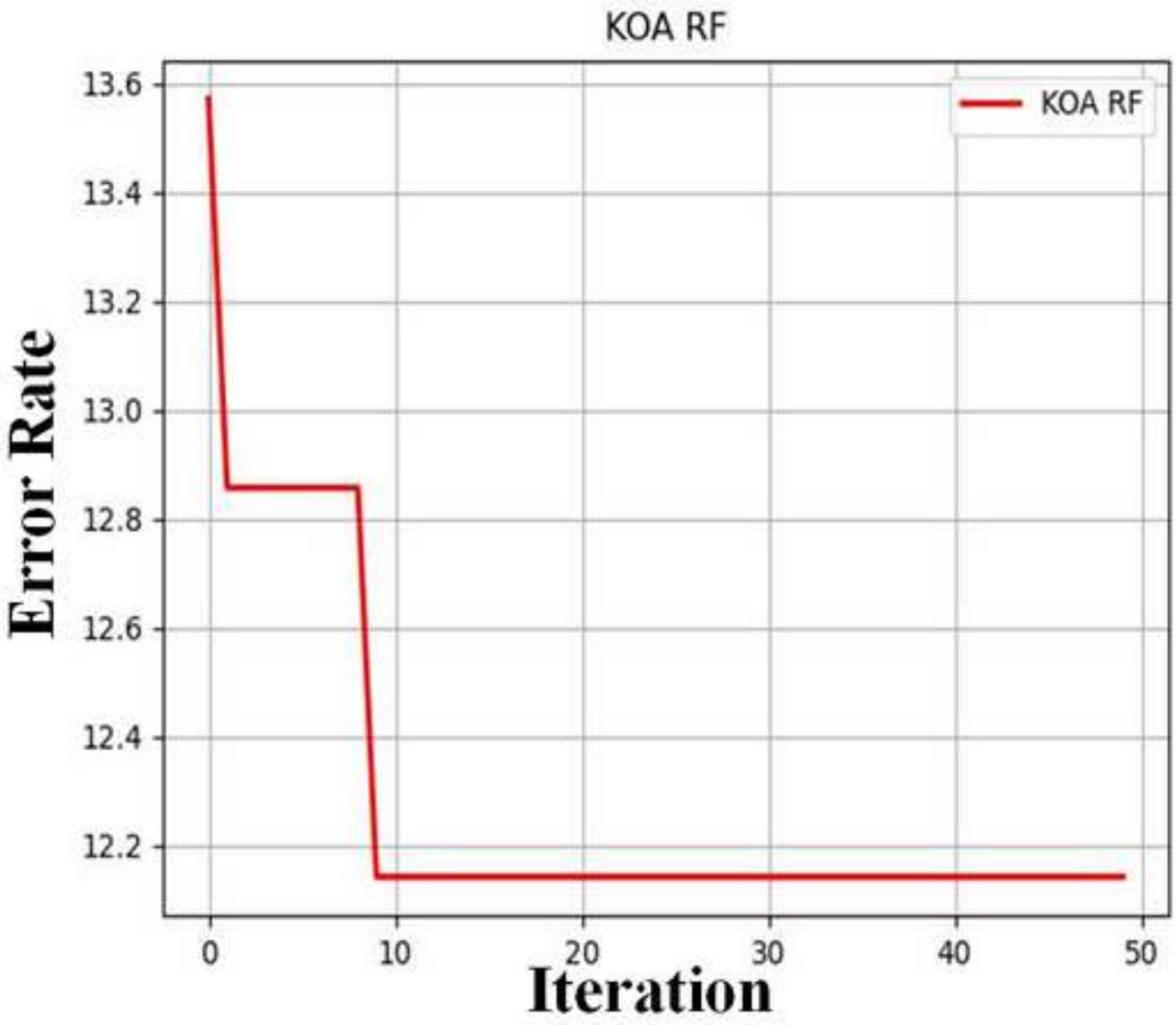

E.KOA-RF

Fig 9.F

Click here to access/download;Figure;Fig 9.F.tif

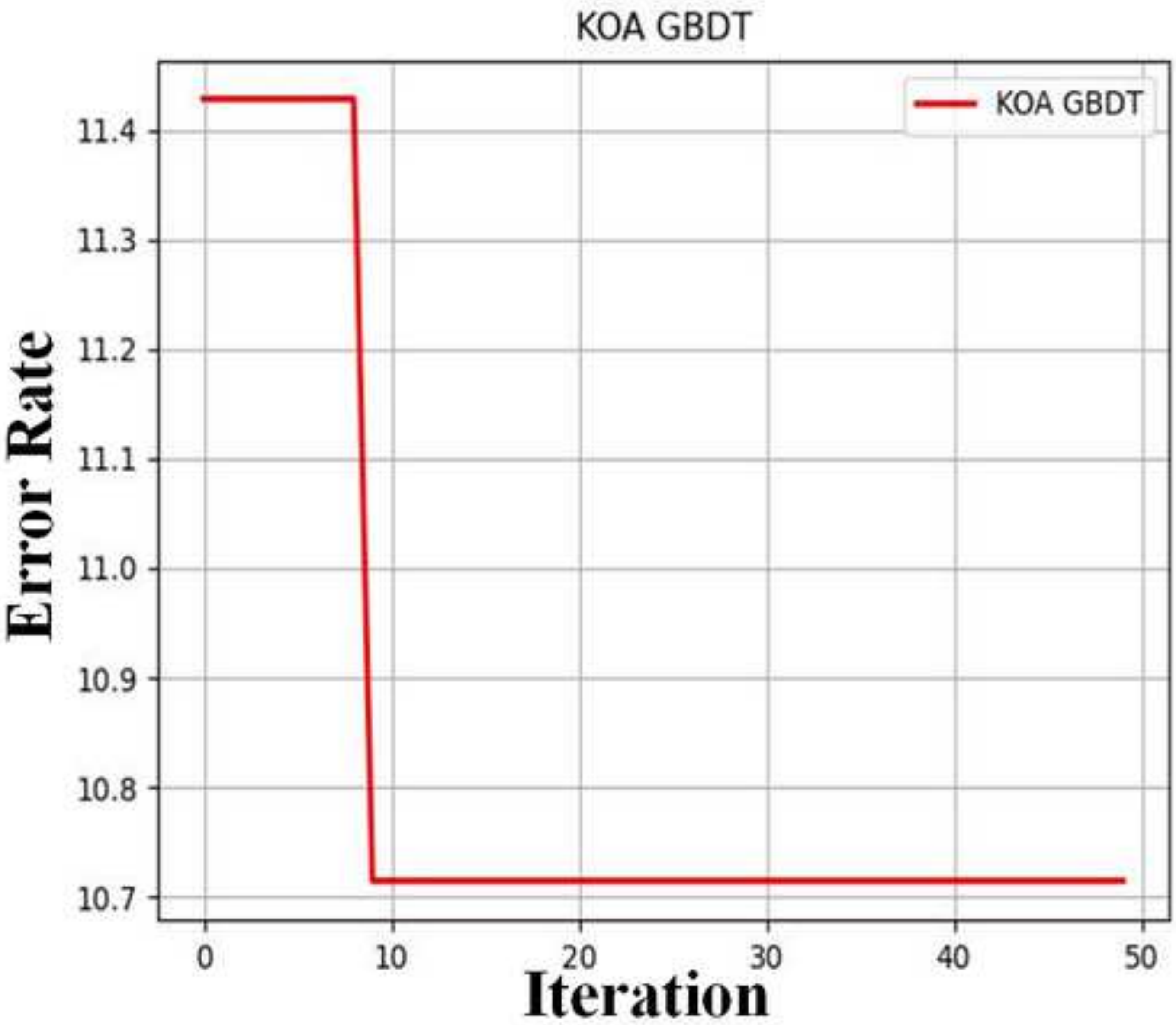

F.KOA-GBDT

Fig 10.A

Click here to access/download;Figure;Fig 10.A.tif

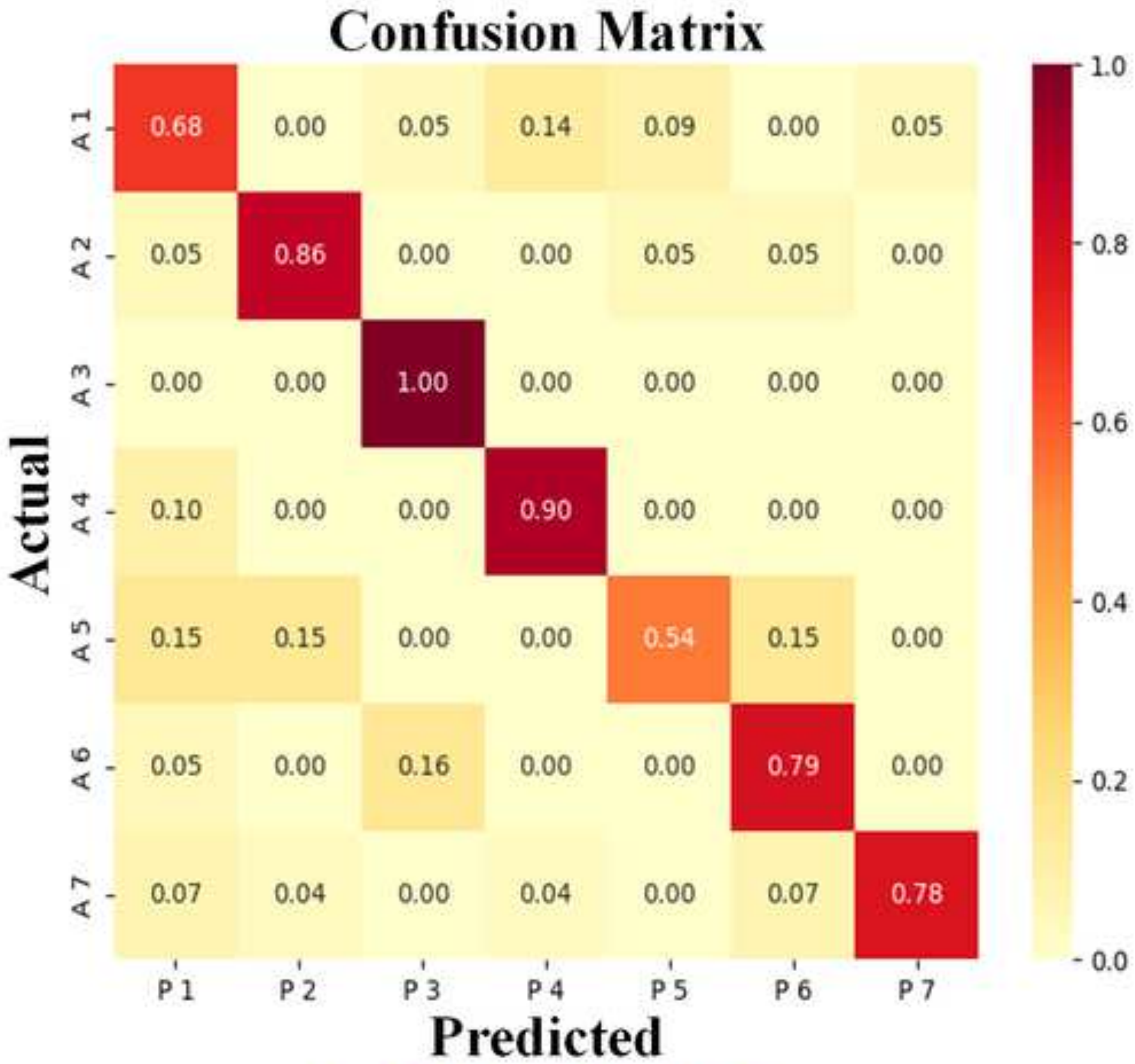

A. DT-STACKING

Fig 10.B

Click here to access/download;Figure;Fig 10.B.tif

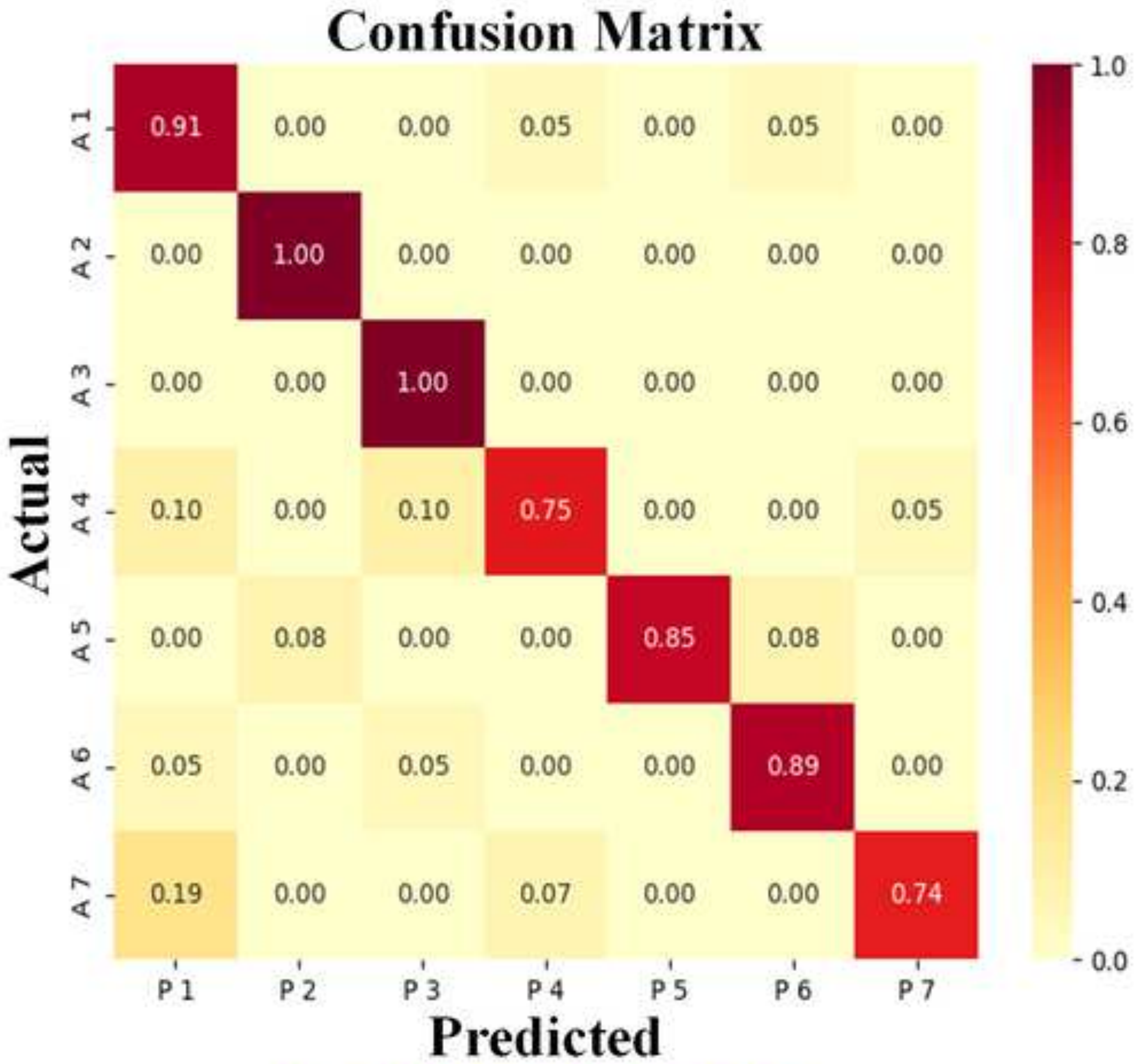

B. SVM-STACKING

Click here to access/download;Figure;Fig 10.C.tif

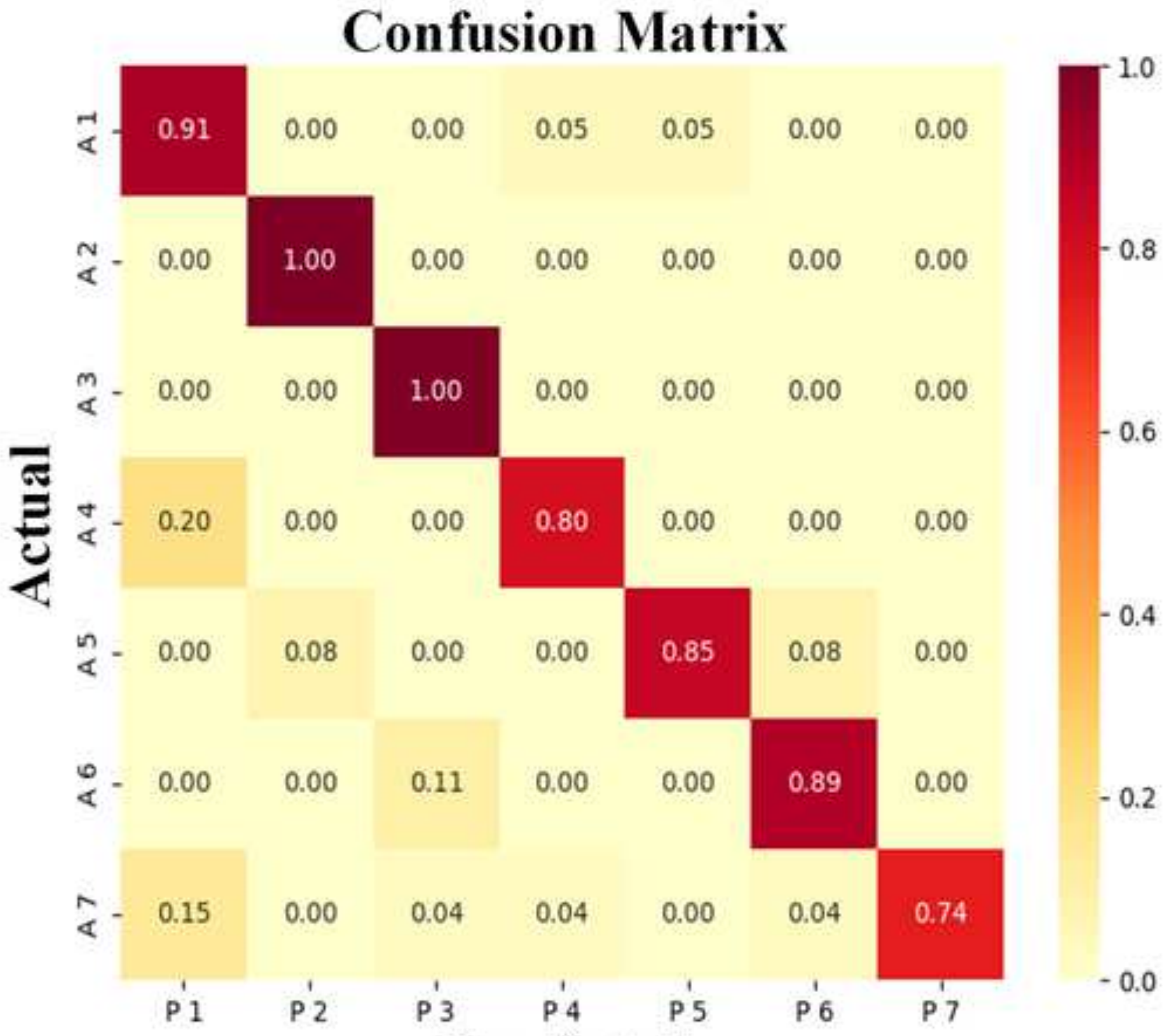

C. KNN-STACKING

Fig 10.D

Click here to access/download;Figure;Fig 10.D.tif

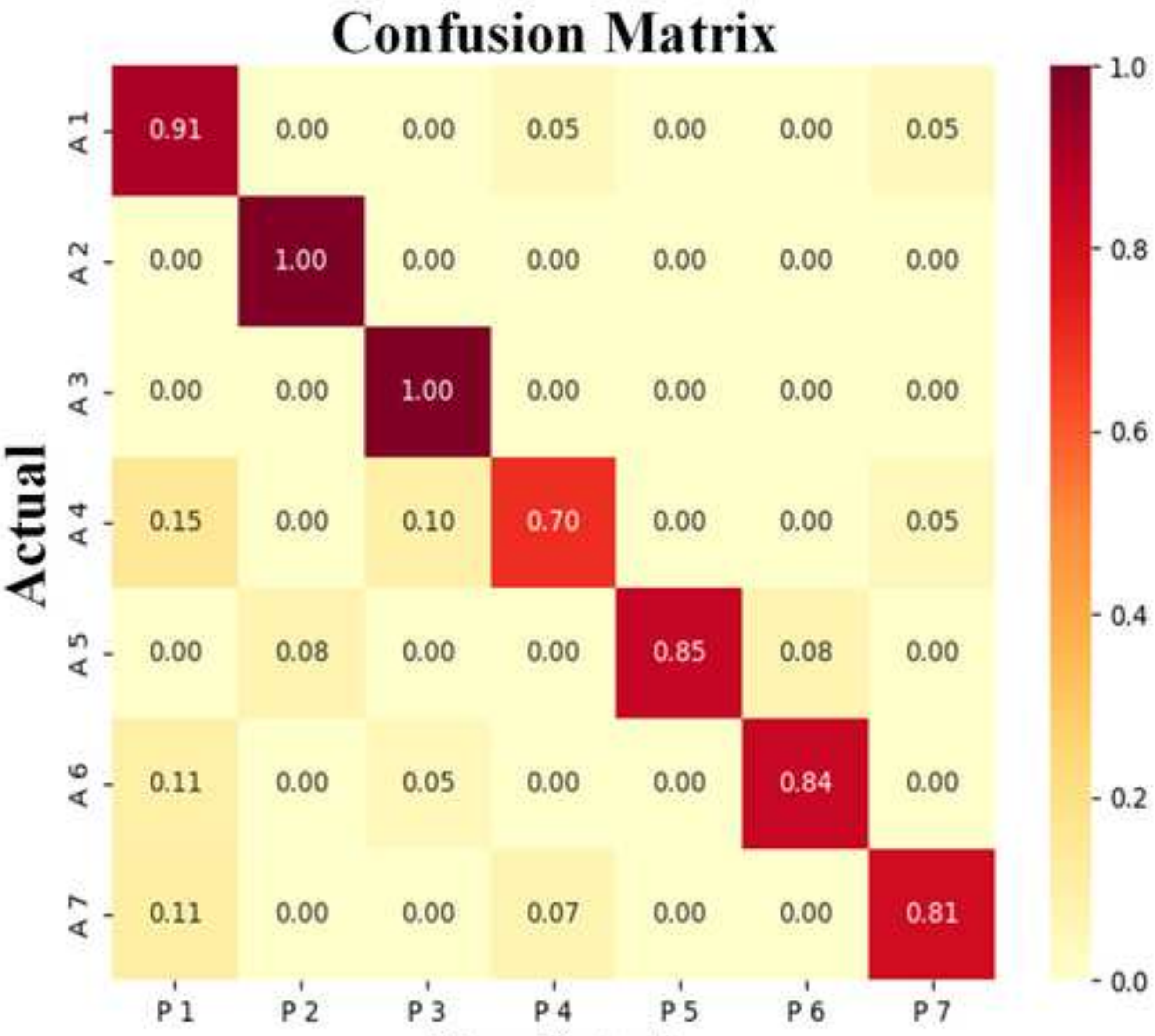

D. MLP-STACKING

Fig 10.E

Click here to access/download;Figure;Fig 10.E.tif

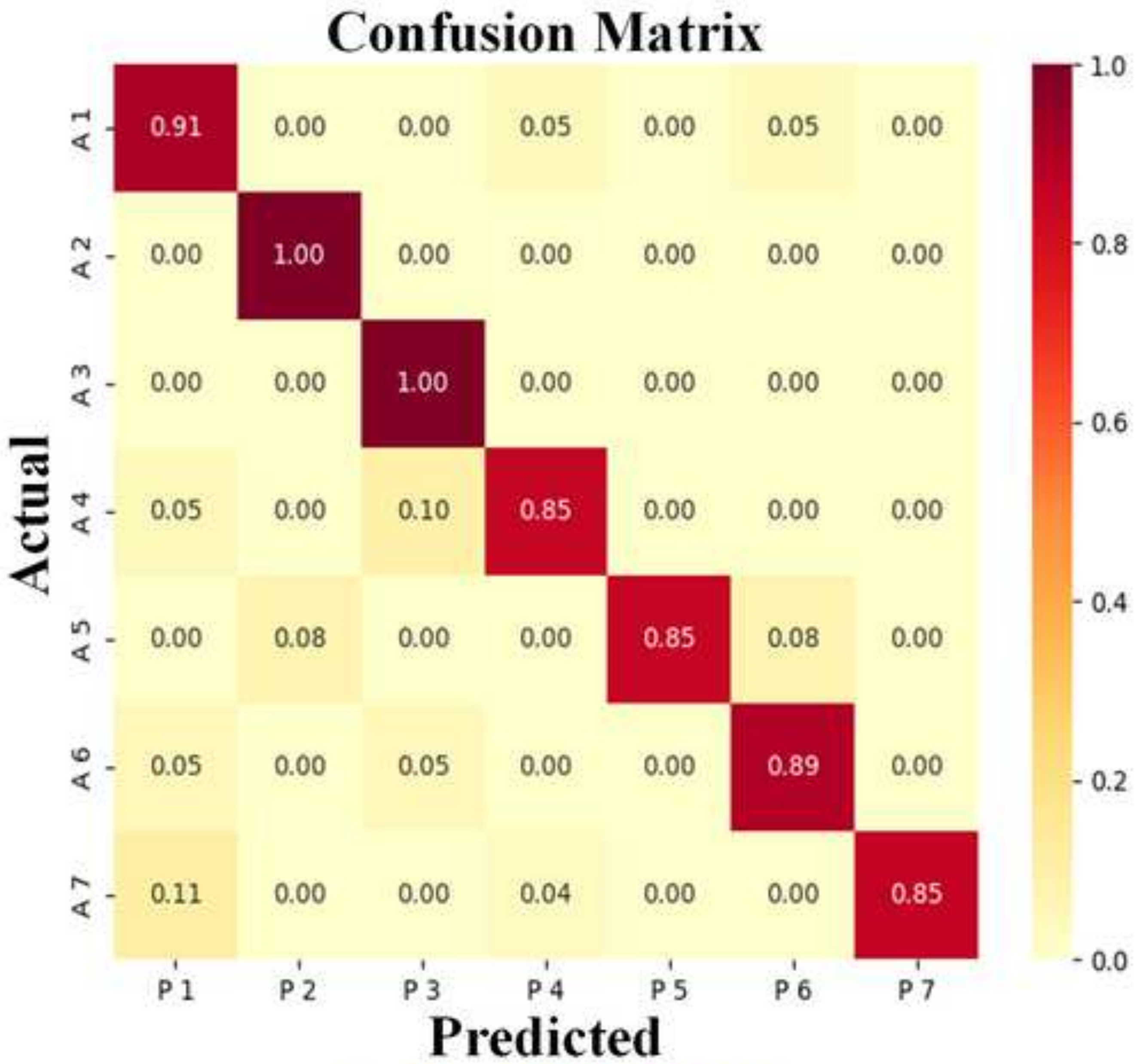

E. RF-STACING

Fig 10.F

Click here to access/download;Figure;Fig 10.F.tif

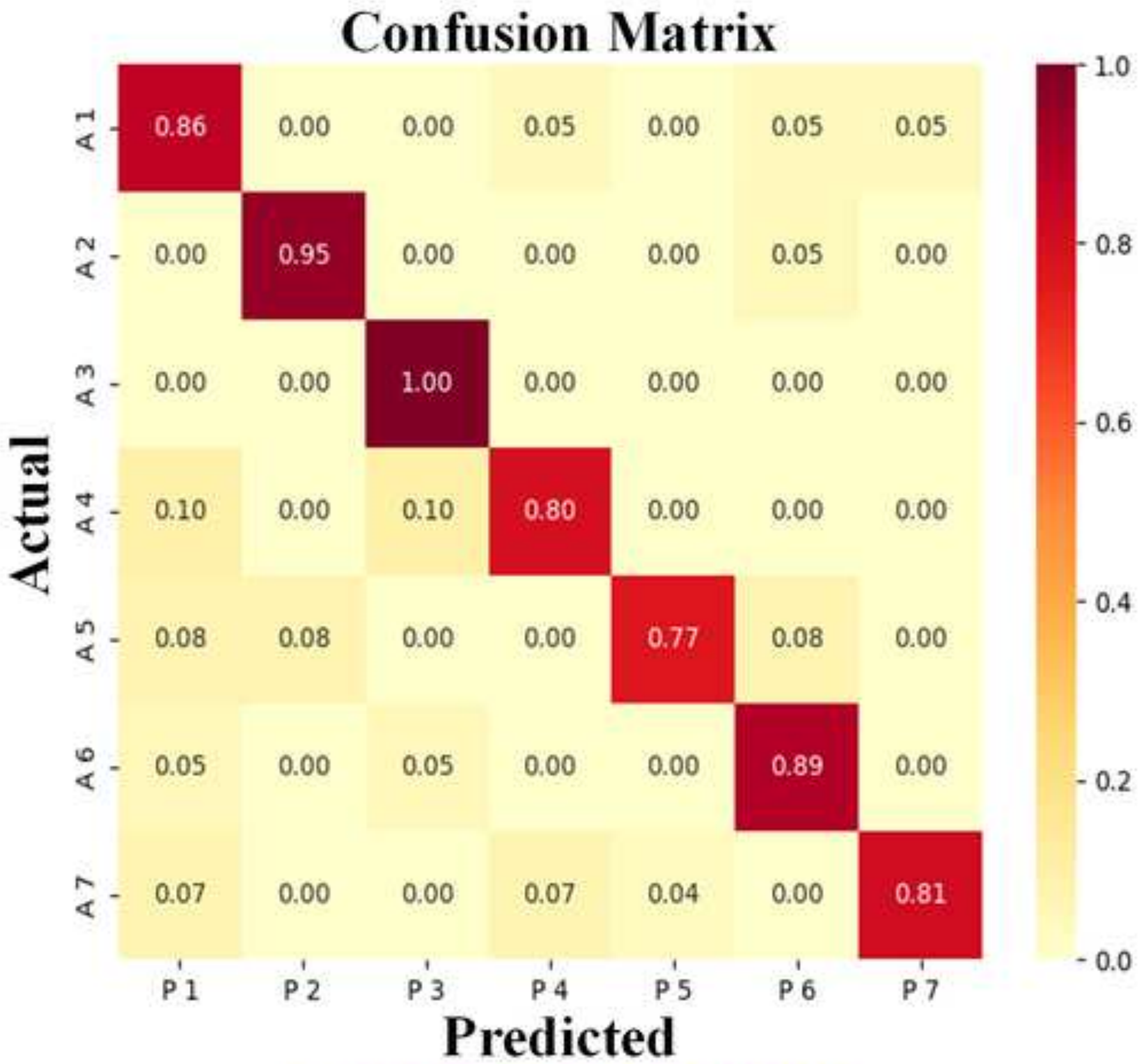

F. GBDT-STACKING

Fig 11.A

Click here to access/download;Figure;Fig 11.A.tif

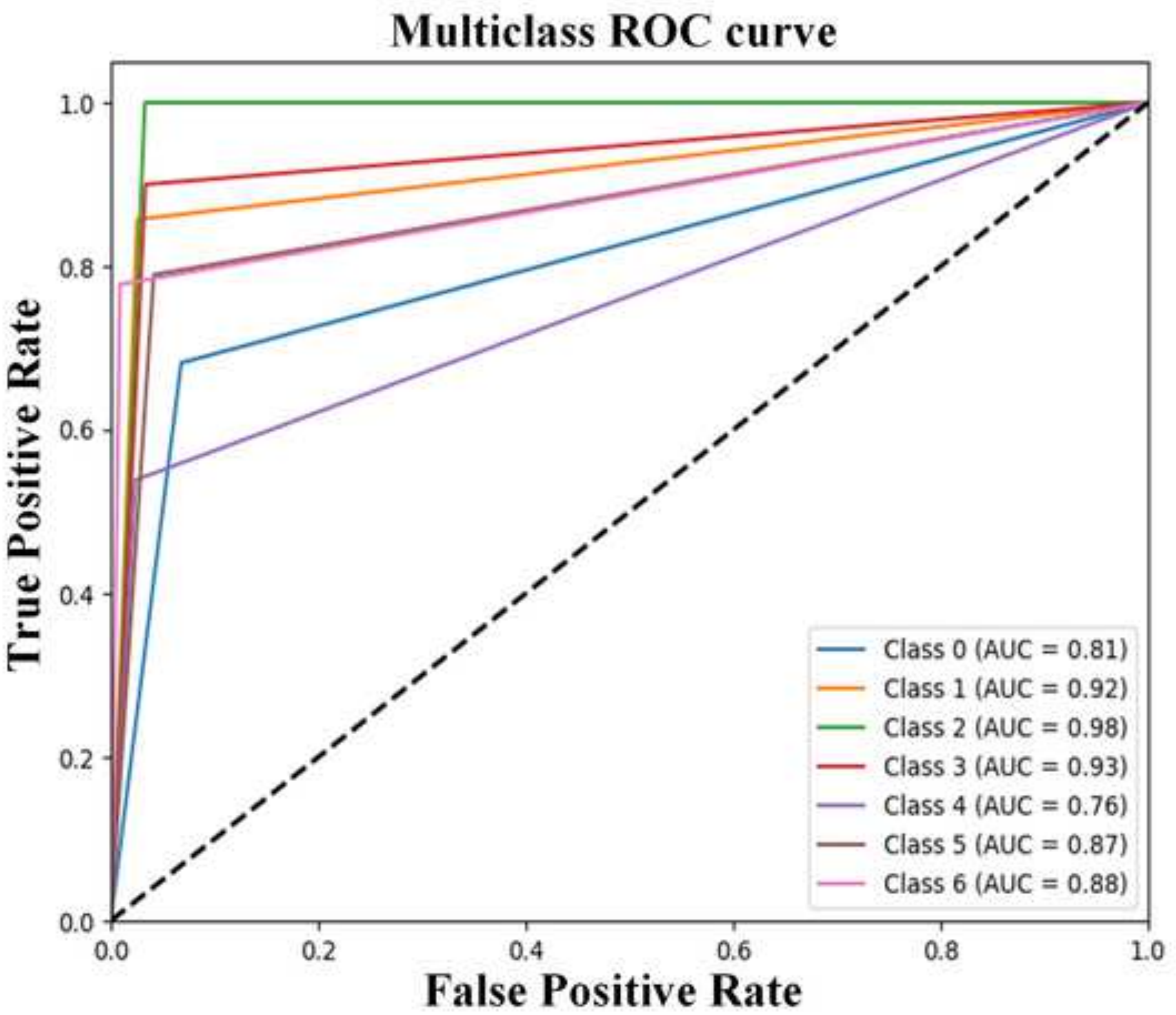

A. DT-STACKING

Fig 11.B

Click here to access/download;Figure;Fig 11.B.tif

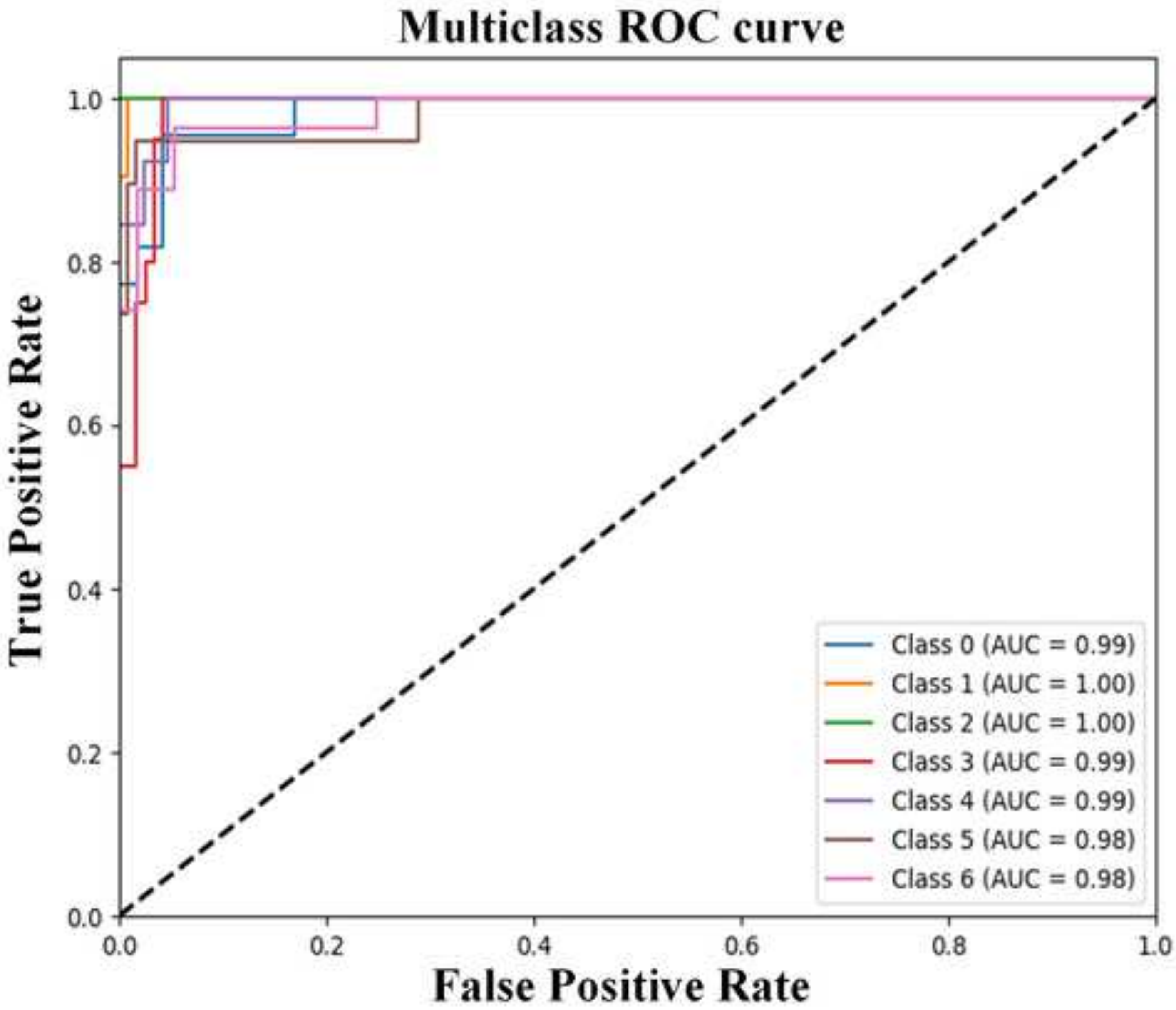

B. SVM-STACKING

Click here to access/download;Figure;Fig 11.C.tif

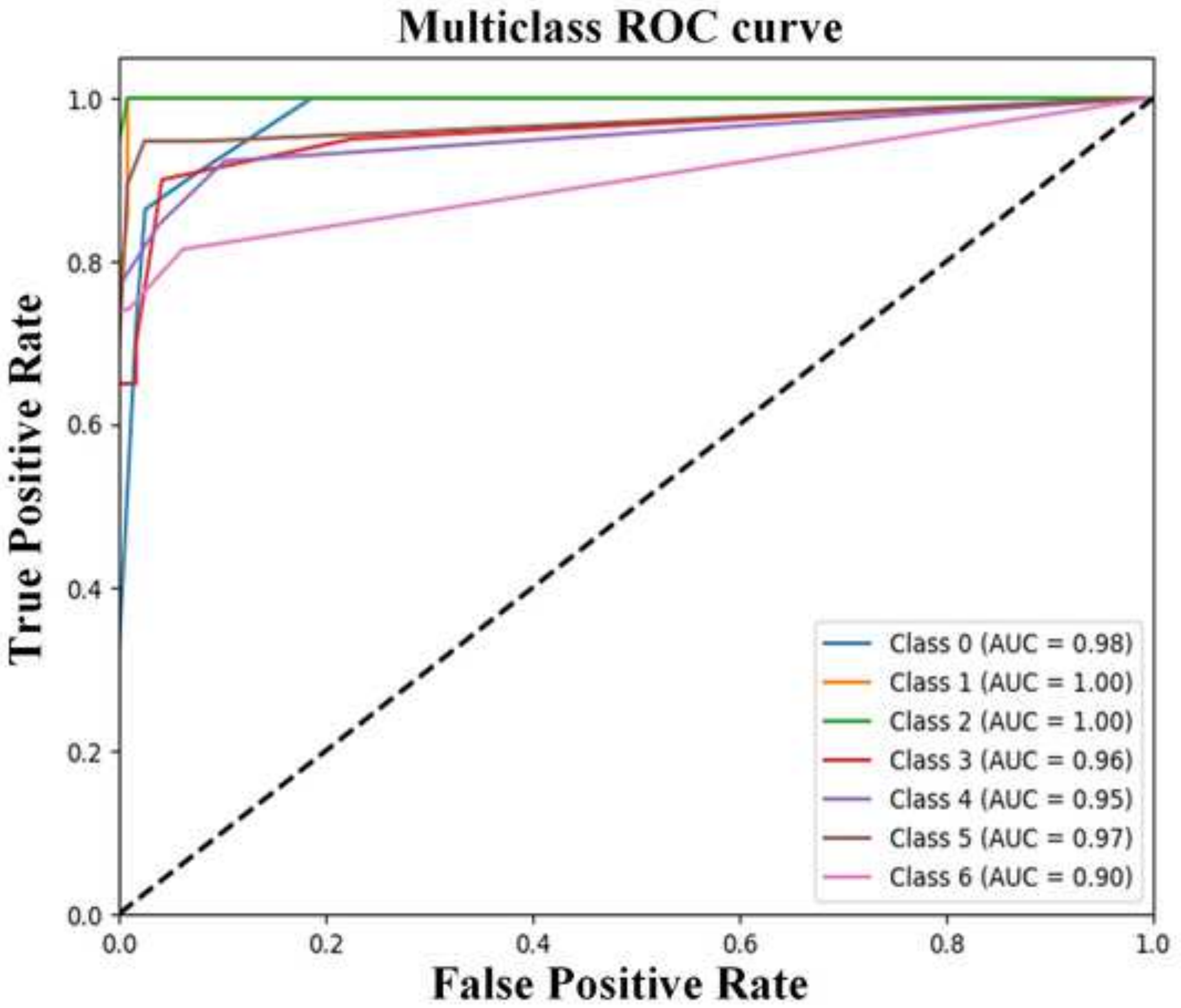

C. KNN-STACKING

Fig 11.D

Click here to access/download;Figure;Fig 11.D.tif

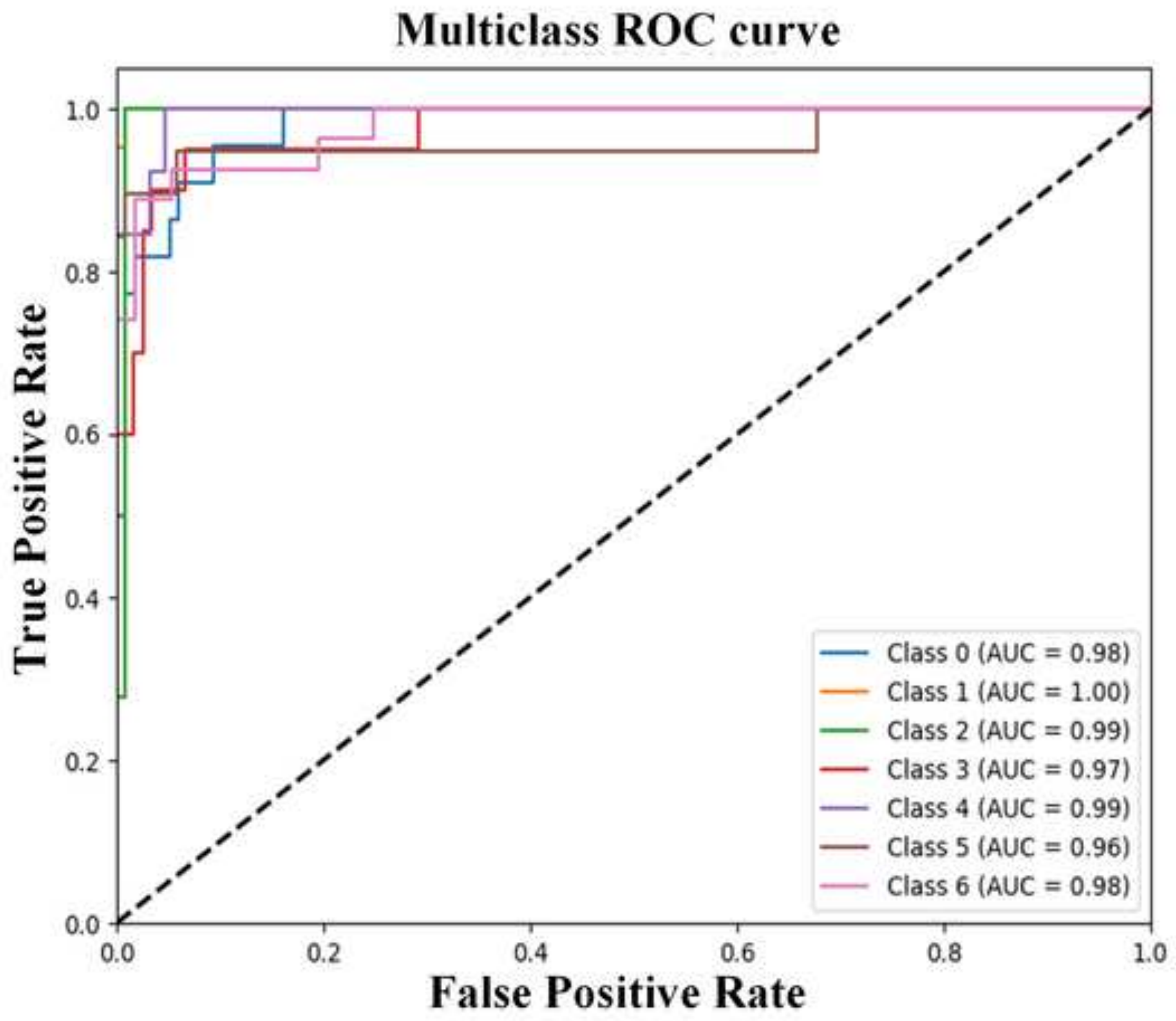

D. MLP-STACKING

Fig 11.E

Click here to access/download;Figure;Fig 11.E.tif

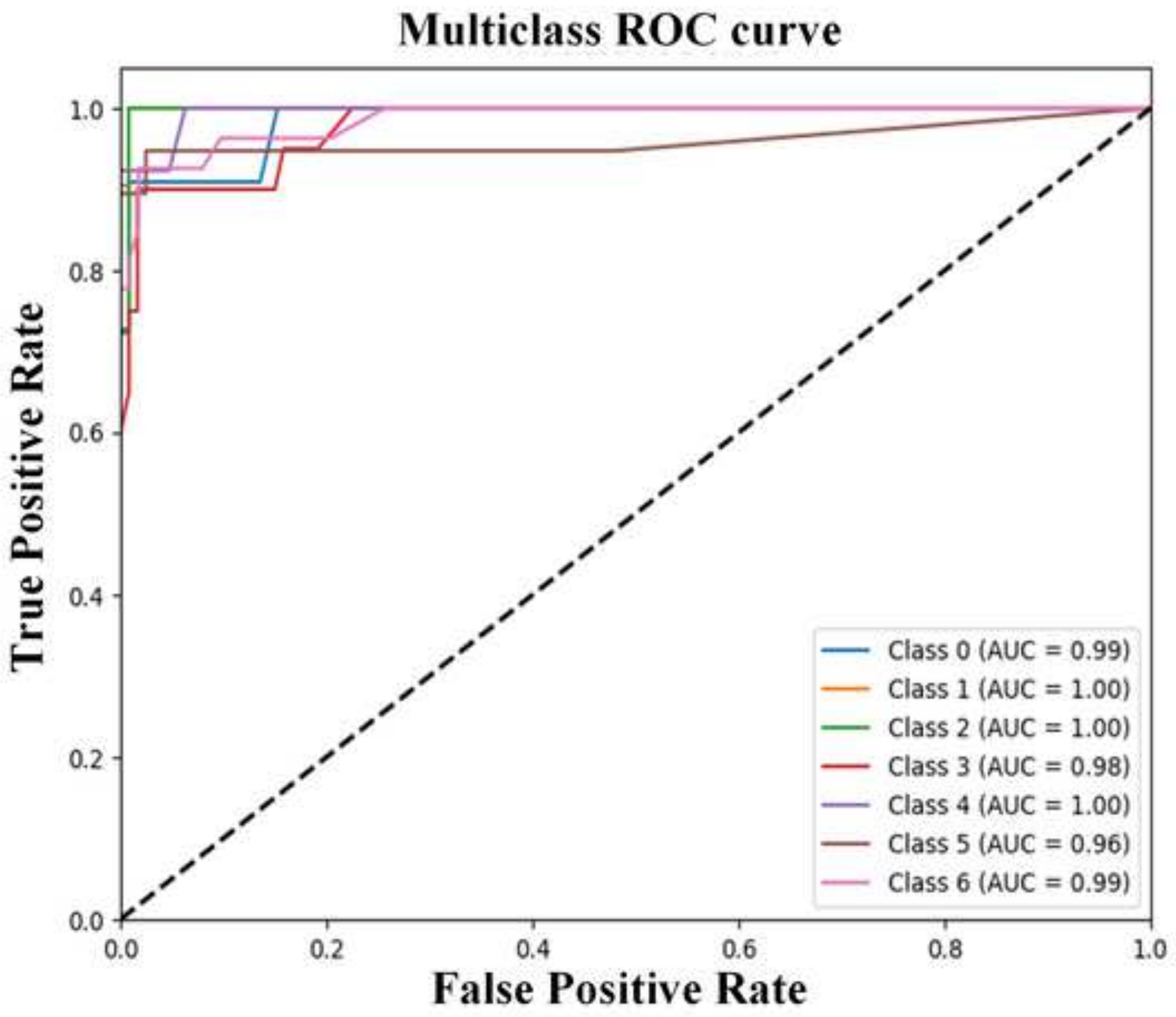

E. RF-STACING

Click here to access/download;Figure;Fig 11.F.tif

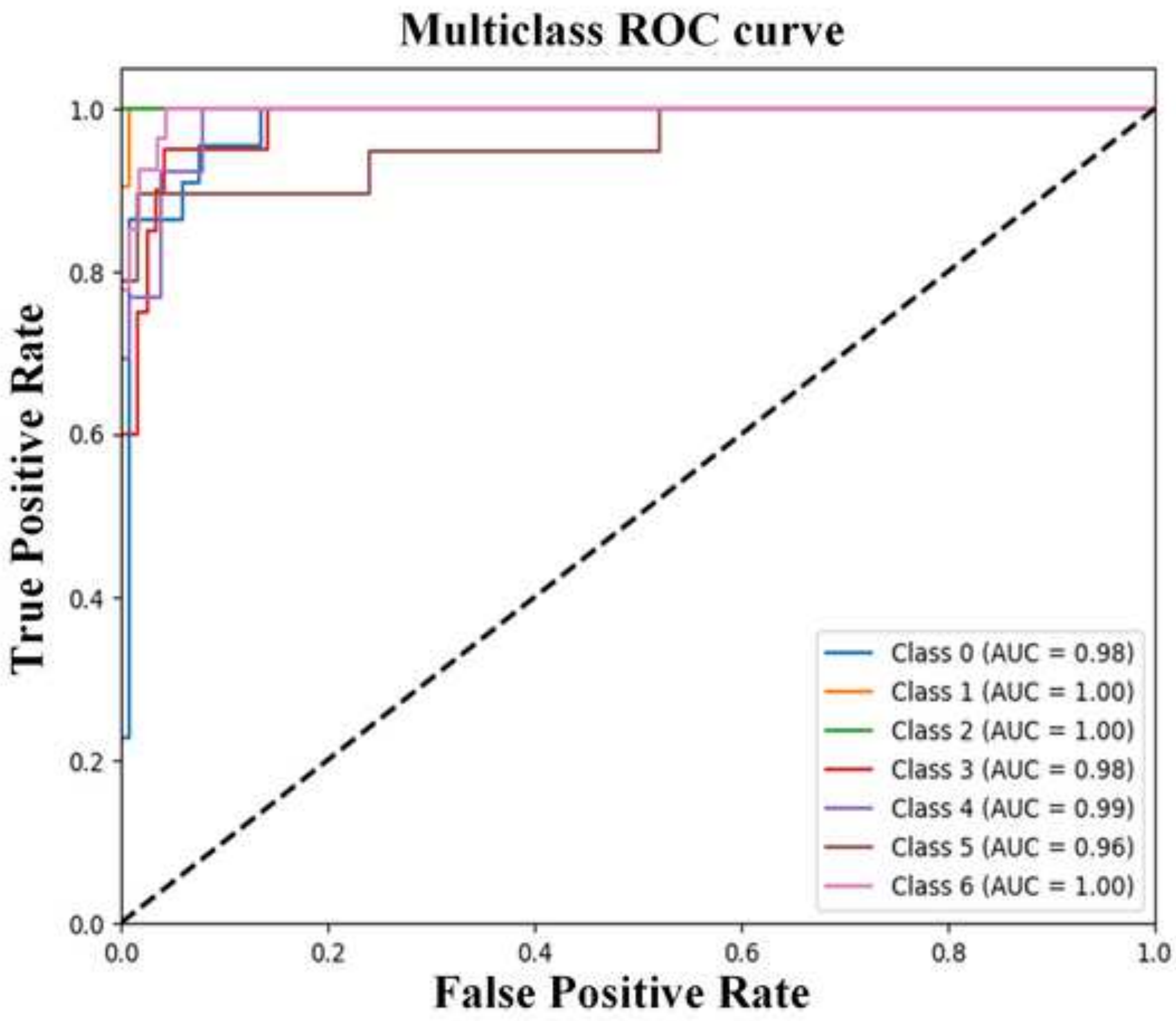

F. GBDT-STACKING

Fig 12

Click here to access/download;Figure;Fig 12.tif

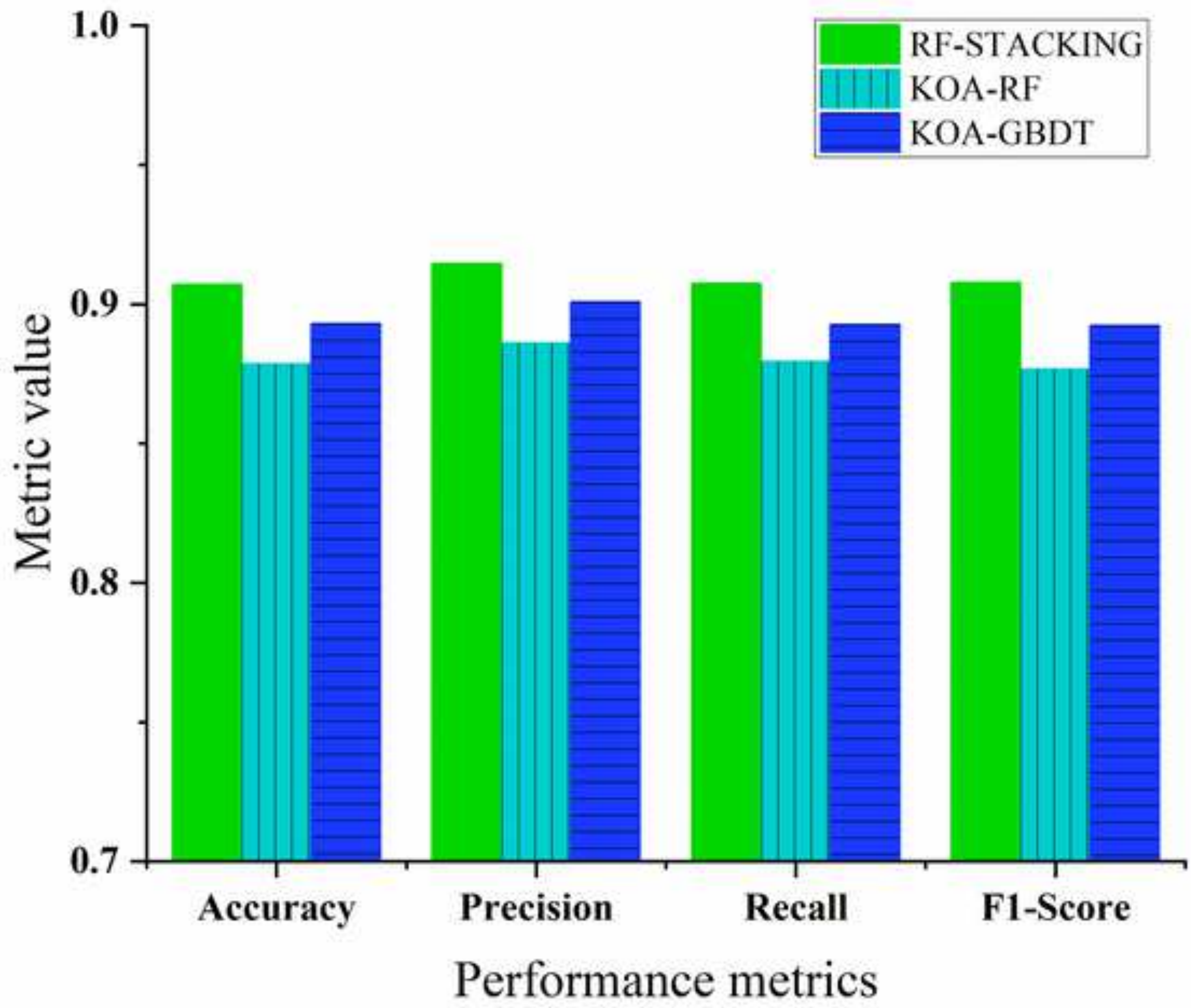

S2 Appendix

Click here to access/download
Supporting Information
S2 Appendix.jpg

S1 Appendix

Click here to access/download
Supporting Information
S1 Appendix.jpg

Mung bean seed classification based on multimodal features and Kepler-optimized stacking ensemble learning model

Shaozhong Song[1,2], Fengwei Leng[1], Ming Fang[1], Xiaofeng An[2], Yaxin Cai[3]

[1] School of Artificial Intelligence, Changchun University of Science and Technology, Changchun, China

[2] School of Data Science and Artificial Intelligence, Jilin Engineering Normal University, Changchun, China

[3] School of Computer Science and Technology, Changchun University of Science and Technology, Changchun, China

*** Correspondence author:**
E-mail: an.xf@jlenu.edu.cn(XA)

Abstract

Accurate classification of mung bean seeds is essential for enhancing both their nutritional value and crop yields. However, current methods are limited, primarily due to the time-consuming and inaccurate classification process resulting from a lack of diverse dataset features. To overcome these challenges, this study develops a multimodal dataset that integrates Raman spectral features and image-based features through early fusion. Furthermore, the classification of mung bean seed varieties is achieved in a rapid, accurate, and non-destructive manner by optimizing a stacking ensemble learning model using the Kepler Optimization Algorithm (KOA). The multimodal dataset comprises 59 features, selected using the Competitive Adaptive Reweighted Sampling (CARS) method. Specifically, 44 key features are extracted from 700 Raman spectral data points, while 15 key features are derived from 43 image-based features. The study also used the Kepler Optimization Algorithm to optimize the parameters of various machine learning models, including Decision Tree (DT), Support Vector Machine (SVM), k-Nearest Neighbor (KNN), Backpropagation Neural Network (BPNN), Random Forest (RF), and Gradient Boosting Decision Tree (GBDT). By constructing a stacking ensemble learning model, the research effectively leverages the strengths of multiple classifiers, thereby enhancing the overall classification performance. Experimental results demonstrate that the proposed method significantly improves mung bean seed classification accuracy, with the Kepler-optimized stacking ensemble model achieving an accuracy of 90.71%. This represents a 3.24% improvement over KOA-RF and a 1.59% improvement over KOA-GBDT. In comparison to baseline models, the proposed method proves to be more efficient.This study underscores the potential of combining multimodal features with a Kepler-optimized stacking ensemble learning model for mung bean seed classification. It highlights the role of advanced artificial intelligence techniques in agricultural production and provides valuable technical support for the precise classification of mung bean seeds.

1. Introduction

Mung beans have been used as an important food in many countries and regions around the world, dating back more than 3500 years (1). Mung beans are consumed in a variety of ways, and they have a long history and widespread use in China, where they have been cultivated for more than 2000 years (2). High-quality seeds are essential for the continuation of good varieties, and they ensure crop yield and quality (3). Seed yield is a key indicator of economic productivity of mungbean, and this indicator is significantly influenced by the variety of mungbean seed (4). In addition, the variety of mungbean seed directly affects its nutrient composition, including key nutrients such as oil, protein, and sugar (5). Therefore, the classification of seed varieties of mungbean as an important food crop is of great value. Various varieties of mung bean seeds exhibit a high degree of morphological similarity. Early researchers primarily relied on the morphological traits of mung bean seeds for classification (6), a method that, while enhancing objectivity, was also labor-intensive and time-consuming. Subsequently, in order to improve the classification efficiency of mung bean seeds, researchers developed DNA fingerprint profiles through chemical methods (7). However, these methods require specialized laboratories and skilled technicians and may potentially damage the seeds. To address the limitations of traditional detection methods, modern techniques such as machine vision and Raman spectroscopy have been adopted for seed classification.

Machine vision primarily emulates the human visual system to process and analyze acquired image data (8). Key components of image information include color, texture, and geometric features (9). The advancement of machine vision technology offers precise, efficient, and non-destructive methods for sensing and data processing in plant phenotyping and precision agriculture (10). For instance, a UNet-based network model has been employed to distinguish between corn and wheat seedlings and weeds in the field (11); a lightweight MssiapNet model was developed to identify different wheat seedling varieties (12); and an improved deep residual convolutional network was applied to identify various corn seed varieties (13). However, machine vision techniques necessitate a

high degree of integrity in the appearance of the target, with even minor defects potentially compromising classification accuracy.

Raman spectroscopy, a form of scattering spectroscopy, relies on inelastic light scattering to obtain chemical information about the internal structure of a substance (14). This technique has proven to be a rapid, non-invasive, and effective tool for quality analysis (15). In recent years, Raman spectroscopy has been applied to identify plant species and their varieties, as well as to assess the nutritional content and commercial value of seeds (16). For example, Raman spectroscopy has been used to classify viable and nonviable maize kernels (17), investigate the microstructure of quinoa fruit seeds and identify the specific location of nutrients within the seeds (18), and combine spectral peak decomposition algorithms with Raman spectroscopy to analyze the oil content in maize seeds (19). These studies highlight the potential of Raman spectroscopy for mung bean seed classification. Although Raman spectroscopy excels in intrinsic quality assessment, it is less effective in appearance evaluation, necessitating further research.

The construction of multimodal datasets that integrate multiple features is gaining prominence in contemporary research. Fusing various types of features can enhance the expressiveness of the data, and by synthesizing information from different features, the limitations of individual feature types can be effectively mitigated. For example, the integration of spectral and spatial features through residual hypergraph convolutional networks has enabled efficient classification of hyperspectral images (20). Data fusion of spectral and image texture features analyzed using SVM has achieved 97.5% accuracy in identifying the year of corn seeds (3). Furthermore, combining morphological features of maize seeds with hyperspectral data has facilitated the classification of maize seed varieties using a random forest model optimized by the Improved Gray Wolf algorithm, achieving an accuracy of 95.9% (21). These findings underscore the effectiveness of constructing multimodal datasets that combine image and spectral features to enhance model classification performance.

Classification using one-dimensional data such as spectral data or image features usually relies on machine learning models (22). Traditional machine learning models include DT (23), SVM (24), KNN (25), and others. Individual machine learning models can be surpassed by integrated learning models that reduce bias and variance and better capture the underlying distribution of the data (26). By fusing multiple simple machine learning models, integrated learning models have been shown to be significantly effective in improving prediction accuracy (27). For example, an integrated learning model combining VGG11, ResNet18, and MobileNet achieved 95.79% accuracy in crop leaf disease identification (28), and RF and GBDT obtained R^2 values of 0.93 and 0.88 in peanut plot yield prediction, respectively (29) based on improved AlexNet, GoogLeNet, ResNet50, and MobileNetV3 as base learners achieved a recognition rate of 99.69% on the rice dataset (30). The performance of machine learning models is affected by model architecture, parameter tuning, and so on. Parameters have a direct effect on the performance of machine learning models, so parameter optimization is a key step in improving model performance. Meta-heuristic optimization algorithms are widely used by researchers to search and optimize the parameters of machine learning models. Meta-heuristic optimization algorithms follow the principle of exploring the search space and utilizing the available information in order to find the optimal solution (31), and the process of finding the optimal solution is based on the assessment of the quality of the solution rather than the specific form of the objective function or the constraints of the problem (32). For example, particle swarm algorithms are used to optimize BP neural networks and RF (33), improved cuckoo search algorithms are used to optimize extreme learning machines (34), and genetic algorithms and genetic dominance algorithms are used to optimize SVM (35). However, metaheuristic algorithms need to make progress in reducing the number of iterations and avoiding local optima to improve the overall performance of the algorithms.

Due to the similarity in appearance, genetic composition, and growth conditions among different varieties of mung bean seeds, accurately and efficiently identifying them presents significant

challenges. To overcome these limitations, this study introduces a novel stacking ensemble learning model for mung bean seed identification. This model leverages a multimodal dataset that combines Raman spectral data and image-based features, while optimizing the parameters of the base learners within a stacking ensemble learning framework using the KOA. The key contributions and innovations of this paper are as follows:

(1). A novel method for constructing datasets is proposed. Raman spectral features and image information features are extracted separately, and subsequently, a multimodal dataset is constructed through early fusion.

(2). A novel approach for model parameter optimization is presented. The key parameters of the base learner in the stacking ensemble learning model are optimized utilizing the KOA.

(3). A novel approach for constructing a stacked ensemble learning model has been employed. Optimized machine learning models serve as base learners, while the selection of the meta-learner model is determined using a combined optimization strategy.

(4). The experimental results confirm the efficiency and practicality of this model for mung bean seed classification, which provides technical support for combating infringement and the production and sale of fake and shoddy seeds, and helps to improve the yield of the mung bean crop and ensure its nutrient content, thus contributing significantly to agricultural production and food safety.

The remainder of the paper is organized as follows: the "Materials and methods" section provides a detailed description of the dataset construction process and the underlying methodology, the "Results and discussion" section presents a partial analysis and interpretation of the experimental results, and the "Conclusion" section offers a summary of the key findings and contributions of the paper.

2. Materials and methods

As illustrated in Fig 1, the classification process of mung bean seeds is organized into six distinct stages: 1. Screening the original mung bean seed samples; 2. Acquiring the Raman spectral features and image information features of the seeds, followed by appropriate preprocessing; 3. Extracting features from both the Raman spectra and image data; 4. Conducting early fusion of the extracted salient features to construct a multimodal dataset.; 5. Inputting the multimodal dataset into a established stacking ensemble learning model; and 6. Outputting the classification results and conducting a data analysis.

Fig 1. The flowchart for mung bean seed classification based on multimodal features and Kepler-optimized stacking ensemble learning model

2.1. Mung bean seed dataset.

The mung bean seed samples used in this experiment were provided by the Research Institute of the Jilin Academy of Agricultural Sciences. These seeds were meticulously selected and are widely distributed in the market. As shown in Fig 2, seven varieties of mung bean seeds were chosen for data collection: Jilv No. 7 (A), Jilv No. 11 (B), Jilv No. 13 (C), Jilv No. 9 (D), Baolv No. 2013 (E), Jilv No. 10 (F), and Jilv No. 5 (G). For each variety, 100 seeds were randomly selected, resulting in a total of 700 data samples.

Fig 2. Selected seven varieties of mung bean seeds

In the acquisition of Raman spectral data for mung bean seeds, this study employed a Hooke D100 near-infrared confocal Raman spectroscopy system, which was equipped with a 785 nm wavelength laser operating at 50 mW power. To capture subtle spectral variations, a precise sampling method was utilized. Raman intensities were recorded at $2cm^{-1}$ intervals across a Raman shift range from $400cm^{-1}$ to $1800\,cm^{-1}$, yielding a total of 700 distinct Raman spectral features.The Raman spectral data collection procedure is described in S1 Appendix.

In this study, mung bean seed images were captured using a Canon EOS 1500D camera, which has an effective resolution of 24.1 megapixels. To extract the necessary image features, the captured images were first converted to grayscale and then processed into binary images to facilitate effective separation of the seeds from the background. Using the binary image as a basis, the original images were accurately segmented, allowing for the extraction of individual mung bean seed images. From these segmented images, a total of 43 key features were derived, including 18 geometric features, 7 texture features, and 18 color features. The image features collection procedure is described in S2 Appendix.

2.2. Data preprocessing

We perform preprocessing operations on the acquired raw Raman spectral data and the image numerical data, respectively.

In this study, we initially apply the Savitzky-Golay (SG) filtering method to reduce noise in the Raman spectra, with the aim of minimizing its influence on the spectral analysis. The choice of window size in the SG filter directly determines the extent of smoothing, while the polynomial order plays a critical role in the filter's ability to adapt to the data's finer details. Given the complexity and richness of the Raman features, a window size of 15 is selected in this work. Moreover, to preserve peak characteristics during the smoothing process, a polynomial order of 3 is employed. Following this, the max-min normalization technique is applied to standardize the spectral data, ensuring that the data are normalized to a consistent scale for comparative analysis.

For the image information features, we apply the max-min normalization method to process the data. This approach is necessary because the collected image features encompass geometric characteristics, textural properties, and color attributes, which can exhibit significantly different value ranges. Max-min normalization ensures comparability across these diverse features.

Following the aforementioned preprocessing steps, we can ensure that all data is standardized and consistent, providing a reliable foundation for subsequent analysis and processing.

2.3. CARS feature extraction

CARS is grounded in Darwin's concept of natural selection, specifically the principle of "survival of the fittest," and enhances feature selection by mimicking the process of biological evolution (37). The principles of CARS feature extraction are illustrated in Fig 3. CARS is distinguished by a two-phase feature extraction process, comprising an initial fast extraction stage followed by a more refined extraction stage.

Fig 3. Principles of CARS feature extraction

The objective of the fast extraction phase is to efficiently eliminate a substantial number of redundant and irrelevant features. During this phase, the exponential degradation function (EDF) is primarily employed to calculate the ratio of retained features and to discard those with minimal weights. For a dataset containing p features with a sampling number N, the proportion of the number of features retained in the ith iteration is given in Eq. (1).

$$r_i = ae^{-ki} \quad (1)$$

Where a and k are constants, in order to ensure that the first sampling includes all features selected, and the Nth sampling includes only two features, the values of a and k must satisfy $r_1 = 1$ and $r_N = 2/p$. The values of a and k can be determined, see Eq. (2):

$$a = \left(\frac{m}{2}\right)^{\frac{1}{N-1}}, \quad k = \frac{ln\left(\frac{m}{2}\right)}{N-1} \quad (2)$$

Following the initial screening of the EDF, a more refined selection of features is conducted using Adaptive Reweighted Sampling (ARS). This technique involves weighted random sampling based on

the current feature weights, where variables with higher weights are more likely to be retained, while those with lower weights are progressively discarded.

2.4. Stacking ensemble learning model

The stacking ensemble learning model enhances predictive performance by utilizing the predictions of multiple base learners as input features for the meta-learner. The principles of stacking ensemble learning model is shown in Fig 4. For a stacking ensemble learning model with T base learners and one meta-learner, the input data is x, then the output Y can be expressed as Eq. (3):

$$Y=f(h_1(x), h_2(x)\dots h_t(x)) \quad (3)$$

where $h_i(\cdot)$ represents the action of the base learner on the input and $f(\cdot)$ represents the action of the meta-learner on the input.

Fig 4. Principles of stacking ensemble learning model

To enhance the generalization performance of the model and mitigate the risk of overfitting, stacking ensemble learning typically employs cross-validation to partition the dataset, which is subsequently used for model training. Assuming a training-to-test set ratio of 4:1, with k-fold cross-validation, the model's training process is outlined as follows:

(1). Data Set Division: The dataset is partitioned into a training set and a test set, with 80% of the data allocated to the training set and 20% to the test set. Subsequently, the training set is randomly divided into k subsets of equal size.

(2). Training and prediction of base learner: Select one subset to serve as the validation set for the base learner, while the remaining $(k-1)$ subsets are used as the training set. For each base learner,

train it using the designated training set. Subsequently, apply the trained base learner to the corresponding validation set to generate prediction results.

(3). Cross-validation: In the second step, the procedure is repeated k times, with each iteration designating a different subset as the validation set. This method allows for the collection of prediction results, denoted as $P_1, P_2 \ldots P_k$, from all base learners across the k iterations of cross-validation. The prediction result P_i for an individual base learner is derived from the aggregation of predictions obtained during the k cross-validation iterations.

(4). Constructing the secondary training set: Constructing the secondary training set involves aggregating the prediction results, denoted as $P_1, P_2 \ldots P_k$, from the base learners along with the corresponding labels from the original training set to create the secondary training set.

(5). Training the meta-learner: The meta-learner is trained using a secondary training set. Its primary objective is to determine the most effective way to integrate the predictions from the first layer of the base learner, thereby enhancing the overall performance of the model.

Once the stacking ensemble learning model has been trained, its overall efficacy and generalization capability can be thoroughly assessed by calculating various performance metrics on an independent test set.

2.5. Kepler Optimization Algorithm

The KOA is a novel metaheuristic algorithm inspired by Kepler's three laws of planetary motion (38). The principles of Kepler Optimization Algorithm is shown in Fig 5. The algorithm employs two strategies: randomly updating planetary positions and adjusting the distances of planets from the Sun. These strategies are designed to balance the algorithm's global and local search capabilities.

Fig 5. Principles of Kepler Optimization Algorithm

In the planetary position update strategy, the KOA algorithm emulates the motion of a planet as it gradually approaches the Sun in one segment of its elliptical orbit and recedes from the Sun in another segment. The position of the planet at iteration $t+1$ is denoted by $\vec{X}_i(t+1)$, which is mathematically represented by Eq. (4).

$$\vec{X}_i(t+1) = \vec{X}_i(t) + \mathcal{F} \times \vec{V}_i(t) + \left(F_{g_i}(t) + |r|\right) \times \vec{U} \times \left(\vec{X}_S(t) - \vec{X}_i(t)\right) \tag{4}$$

$\vec{V}_i(t)$ is the velocity that moves the planet from position $\vec{X}_i(t)$ to $\vec{X}_i(t+1)$, and $F_{g_i}(t)$ is the gravitational force exerted by the Sun on the planet at position $\vec{X}_i(t)$.

In the strategy of updating the distance between the planet and the Sun, the KOA algorithm simulates the typical distance behavior that naturally changes over time between the Sun and the planet. At this point, the position $\vec{X}_i(t+1)$ can be obtained by Eq. (5).

$$\begin{aligned}\vec{X}_i(t+1) = \vec{X}_i(t) \times \vec{U}_1 + \left(1 - \vec{U}_1\right) \times \left(\frac{\vec{X}_i(t) + \vec{X}_S + \vec{X}_a(t)}{3}\right) \\ + \left(1 - \vec{U}_1\right) \times h \times \left(\frac{\vec{X}_i(t) + \vec{X}_S + \vec{X}_a(t)}{3} - \vec{X}_b(t)\right)\end{aligned} \tag{5}$$

In this context, the regulating parameter h plays a pivotal role. When its value is large, the exploration operation is employed to increase the distance between the planet's orbit and the Sun. Conversely, when the value is small, the Sun is positioned closer to the planets, and the exploitation operation is utilized to refine the region near the optimal solution.

3. Results and discussion

The stacking ensemble learning model adopted in this paper consists of two layers: base learners and a meta-learner. It fuses the prediction results of multiple base learners of different types and then conducts final integration through the meta-learner. By leveraging the complementarity among models, it captures the characteristics and patterns of data from multiple perspectives, effectively reducing errors and significantly improving the accuracy and generalization ability of prediction and classification. In view of this, selecting high - performing base learner models and meta-learner models is of great importance. In this paper, the KOA is first applied to optimize the parameters of six classification models,including SVM, DT, KNN, RF, MLP, and AdaBoost. The optimized models are then used as the base learners of the stacking ensemble learning model. Subsequently, the meta-learner model is selected through a combinatorial optimization approach, and finally, the stacking ensemble learning model used in this paper is constructed.

The hardware specifications employed in this experiment are as follows: the processor is an Intel Core i5-12400F with a clock speed of 2.50 GHz, the system is equipped with 16 GB of RAM, and the graphics card utilized is an NVIDIA RTX 4060 Ti.

3.1. Feature extraction and result analysis

In this study, CARS was utilized to extract features from both the Raman spectral dataset and the image feature dataset, as depicted in Fig 6. In the context of CARS-based feature extraction, the features are conceptualized as “organisms,” and the predictive performance of the model serves as an indicator of their “fitness.” The model’s prediction performance is treated as a measure of “adaptation.” During each iteration, a subset of the extracted features is utilized in the Partial Least Squares Regression model, followed by cross-validation. The performance of each feature subset is evaluated by calculating its Root Mean Square Error of Cross-Validation (RMSECV), with the subset yielding the lowest RMSECV being selected as the optimal feature subset.

Fig 6. CARS feature extraction iterative process

Fig 6.A illustrates the feature extraction process of the CARS method applied to the Raman spectral feature dataset. The process concludes after reaching the predefined maximum iteration count of 20. As depicted in Fig 6.A, the RMSECV reaches its minimum value of 1.58 at the 9th iteration. At this point, the number of selected features is 44, representing only 6.28% of the original feature set. The key features obtained during the extraction process of Raman spectral data are presented in Fig 7.A. Including 486cm^{-1}, 504cm^{-1}, 506cm^{-1}, 616cm^{-1}, 626cm^{-1}, 630cm^{-1}, 640cm^{-1}, 672cm^{-1}, 684cm^{-1}, 788cm^{-1}, 878cm^{-1}, 924cm^{-1}, 928cm^{-1}, 934cm^{-1}, 968cm^{-1}, 970cm^{-1}, 976cm^{-1}, 990cm^{-1}, 994cm^{-1}, 1012cm^{-1}, 1140cm^{-1}, 1150cm^{-1}, 1338cm^{-1}, 1342cm^{-1}, 1344cm^{-1}, 1352cm^{-1}, 1356cm^{-1}, 1444cm^{-1}, 1446cm^{-1}, 1484cm^{-1}, 1492cm^{-1}, 1500cm^{-1}, 1506cm^{-1}, 1518cm^{-1}, 1524cm^{-1}, 1590cm^{-1}, 1592cm^{-1}, 1714cm^{-1}, 1716cm^{-1}, 1722cm^{-1}, 1728cm^{-1}, 1732cm^{-1}, 1770cm^{-1}, 1772cm^{-1}.

Fig 7. Key features obtained during the CARS extraction process

Fig 6.B illustrates the feature extraction process of the CARS method applied to the image information feature dataset. Similarly, The RMSECV curve reaches its lowest point, with an RMSECV value of 1.48, at 6th iterations, where 15 features are selected. This represents 34.88% of the total number of initial features. The key features for image information extraction, as shown in Fig 7.B. Including 1. perimeter, 2. area, 3. long axis length, 4. short axis length, 22. grey scale variance product function, 25. vollath function, 26. r-mean in RGB space, 27. G-mean in RGB space, 29. R-variance in RGB space, 32. H-mean in HSV space, 34. V-mean in HSV space, 37. V-variance in HSV space, 38. L-mean in LAB space, 39. A-mean in LAB space, 42. A-variance in LAB space.

3.2. Performance evaluation of multimodal datasets

Through early fusion, we developed a multimodal feature dataset comprising a total of 59 features by combining 44 features derived from the Raman spectral dataset with 15 features extracted from the

image information dataset. This fusion process is designed to integrate information from diverse data sources to enhance model performance and improve prediction accuracy. To investigate the impact of the constructed multimodal feature dataset on classification tasks, this study employs the classical DT model in machine learning to assess the classification performance across five datasets: the Raman spectral feature dataset, the image information feature dataset, the Raman spectral subset selected using CARS, the image information subset selected using CARS, and the multimodal feature dataset.

In this study, the dataset was divided into training and test sets at a ratio of 4:1, and performance evaluation results were obtained for five distinct datasets in the task of mung bean seed classification, as illustrated in Figure 8. When comparing the classification performance of the decision tree model applied to the Raman spectral feature dataset with that of the subset of Raman spectral features extracted using CARS, we observed a 13.79% improvement in classification accuracy, while the runtime was substantially reduced to only 6.28% of the original. Similarly, when evaluating the classification performance of the decision tree model on the image information feature dataset and its subset extracted via CARS, the classification accuracy increased by 4.11%, while the runtime decreased to 41.74% of its original value. Furthermore, when the decision tree model was applied to the multimodal dataset, the highest classification accuracy of 76.43% was achieved, with minimal increase in runtime. These results suggest that the multimodal feature dataset significantly enhances classification accuracy while also improving decision-making efficiency.

Fig 8. Comparison of Classification Performance Across Various Datasets

3.3. KOA optimizes the parameters of the base learner models

To ensure that the stacking ensemble learning model effectively captures the intrinsic features of the data from multiple perspectives, thereby enhancing predictive performance, this study carefully selected a set of base learners, drawing on the choices made by previous researchers (39,40). The base learners chosen for the first layer of the ensemble model include: SVM, DT, KNN, RF, MLP and

AdaBoost. To further improve the performance of these base learners, the study applied the KOA to optimize the model's parameters. After balancing search efficiency with accuracy, the population size was set to 10, and the maximum number of iterations was limited to 50. Additionally, the model's error rate was used as the fitness function. The parameter optimization ranges and the corresponding optimal values are presented in Table 1.

Table 1. KOA optimize base learner parameters

Basic model	Parameter Optimization Range	Optimal Parameters
DT	max_depth{1,100}, max_features{0.1,1}	max_depth = 13, max_features = 0.45
SVM	c{1,1000}, gamma{0.1,1}	C = 828.92 , gamma = 0.56
KNN	n_neighbors{1,100}, p{1,5}	n_neighbors = 3, p = 2
MLP	hidden_layer_sizes(k1,k2): k1{10,100}, k2{10,100}	hidden_layer_sizes(k1,k2): k1 = 96, k2 = 74
RF	n_estimator{1,100}, max_depth{1,100}, max_features{0.1,1}	n_estimator = 92, max_depth = 78, max_features = 0.17
GBDT	n_estimator{1,100}, max_depth{1,100}, max_features{0.1,1}	n_estimator = 84, max_depth = 20， max_features = 0.46

The KOA is capable of updating the best solution in each iteration, with the elite strategy ensuring that a superior candidate solution replaces the current best solution. This iterative process continues until the termination criteria, such as reaching the maximum number of iterations, are met. At this point, the algorithm concludes, and the current best solution is considered the optimal solution, marking the completion of the optimization process. The KOA demonstrates high efficiency in parameter optimization, as illustrated in Fig 9. In the parameter optimization task, the DT model attains a fitness function value of 0.2 in the first iteration. In contrast, the SVM model requires considerably more iterations to reach a fitness function value of 0.1214 in the 25th iteration. The KNN model achieves a fitness function value of 0.1571 by the 9th iteration, MLP model reaches a value of 0.1143 by the 8th

iteration, RF model attains 0.1214 by the 9th iteration, and GBDT model reaches a value of 0.1071 by the 9th iteration. All of these models converge to the optimal parameter combinations relatively quickly, within 10 iterations. The SVM model, however, requires a higher number of iterations to identify the optimal parameter set, which may be attributed to the sensitivity of the SVM model's parameters to variations in model performance, necessitating more iterations to achieve optimal results.

Fig 9. The process of KOA optimizing base learning models

The performance metrics of the six models—KOA-DT, KOA-SVM, KOA-KNN, KOA-MLP, KOA-RF, and KOA-GBDT—optimized using the KOA algorithm are presented in Table 2. Among these, the KOA-GBDT model demonstrates the best performance, achieving an accuracy of 0.8929, which represents a 2.63% improvement over the average accuracy of 0.8700 across all six models. Additionally, its precision is 0.9008, which is approximately 3.60% higher than the average precision of 0.8695; its recall is 0.8927, showing a 2.75% increase over the average recall of 0.8688; and its F1 score is 0.8923, which is 2.35% higher than the average F1 score of 0.8718. These results underscore the effectiveness of the KOA algorithm in optimizing the parameters of machine learning models. By optimize these parameters, the model is able to more accurately capture data patterns during the learning process, thereby enhancing its predictive performance.

Table 2. Performance of models following KOA optimization

Optimized model	Accuracy(%)	Precision(%)	Recall(%)	F1-Score(%)
KOA-DT	0.8	0.7997	0.8176	0.8024
KOA-SVM	0.8786	0.8878	0.8818	0.8800
KOA-KNN	0.8429	0.8478	0.8510	0.8408
KOA-MLP	0.8857	0.8956	0.8894	0.8886
KOA-RF	0.8786	0.8860	0.8794	0.8766
KOA-GBDT	0.8929	0.9008	0.8927	0.8923

3.4. Stacking ensemble learning model construction and analysis

In constructing the stacking ensemble learning model, after optimizing the first layer of base learners, we employ combinatorial optimization to select the appropriate meta-learner. Experiments are conducted using DT, SVM, KNN, MLP, RF and GBDT as meta-learners. The resulting models are referred to as DT-stacking, SVM-stacking, KNN-stacking, MLP-stacking, RF-stacking, and GBDT-stacking, respectively. This approach aims to evaluate which meta-learner combination maximizes the classification performance of the model.

Table 3 demonstrates the classification performance of the six final stacking ensemble learning models constructed by this method. Among these models, the RF-stacking model has the best classification performance with 90.71% accuracy, 91.45% precision, 90.74% recall and 90.77% F1-Score. Compared to the KNN-stacking model, which has the second highest classification performance, RF-stacking is 3.24% higher in accuracy, 2.81% higher in precision, 2.60% higher in recall, and 3.02% higher in F1-Score.

Table 3. Performance of six stacking ensemble learning models

Stacking model	Accuracy(%)	Precision(%)	Recall	F1-Score
DT-stacking	0.8	0.7929	0.7921	0.7880
SVM-stacking	0.8714	0.8866	0.8772	0.8763
KNN-stacking	0.8786	0.8895	0.8844	0.8811
MLP-stacking	0.8714	0.8868	0.8732	0.8750
**RF-stacking**	**0.9071**	**0.9145**	**0.9074**	**0.9077**
GBDT-stacking	0.8714	0.8753	0.8707	0.8699

Fig 10 illustrates the confusion matrices of six stacking ensemble learning models, highlighting their performance in classifying mung bean seeds. Among these models, DT-stacking exhibits poor performance in classifying Baolv No. 2013 and Jilv No. 7. SVM-stacking struggles to differentiate between Jilv No. 9 and Jilv No. 5, while KNN-stacking shows weak identification accuracy for Jilv

No. 5. MLP-stacking faces challenges in classifying Jilv No. 9, and GBDT-stacking also fails to effectively classify Baolv No. 2013. The classification of Jilv No. 9, Baolv No. 2013, and Jilv No. 5 proves particularly difficult across the models, with RF-stacking demonstrating the best overall classification performance.

Fig 10. Confusion matrix for six stacking ensemble learning models

Fig 11 illustrates the Receiver Operating Characteristic (ROC) curves for six stacking ensemble learning models, which are employed to evaluate the performance of the classifiers. The ROC curves are quantified by the Area Under the Curve (AUC), with higher AUC values indicating superior performance. In the context of multi-class classification, ROC curves are generated for each individual class to assess the model's ability to differentiate between them. The figure reveals that the DT-stacking model struggles to distinguish between Baolv No. 2013 and Jilv No. 7, while the Random Forest RF-stacking model demonstrates robust performance across all categories, achieving an average AUC value of 0.9886. Based on these results, we have selected RF model as the meta-learner in the stacking ensemble to optimize classification performance.

Fig 11. ROC curves for six stacking ensemble learning models

As shown in Fig 12, the RF-stacking model demonstrates a substantial performance advantage over the two classical integrated learning model, KOA-RF and KOA-GBDT, across all four key performance metrics: accuracy, precision, recall, and F1-Score. Specifically, improvements of 3.24%, 3.22%, 3.18%, and 3.55% for accuracy, precision, recall, and F1-Score, respectively, are observed for the RF-stacking model compared to KOA-RF, and improvements of 1.59%, 1.52%, 1.65%, and 1.73% compared to KOA-GBDT. These results underscore the superior integrated classification performance of the RF-stacking model, making it the most effective model among those evaluated.

Fig 12. Comparison of classification performance of different ensemble learning models

4. Conclusion

This study introduces, for the first time, the stacking ensemble learning model based on multimodal features and the KOA, demonstrating strong performance in the classification of mung bean seeds. The experimental procedure and results are as follows: Initially, 44 key features are extracted from 700 Raman spectral features using the CARS feature extraction method, and 15 key features are extracted from 43 numerical image features. These 59 combined features form a multimodal dataset, which integrates information from multiple data sources while eliminating redundant features, thereby enhancing classification efficiency and accuracy. Subsequently, the KOA is employed to effectively search for optimal parameters for the base learner model, further improving classification performance. Finally, the optimized base learner model is used to construct a stacking ensemble learning model, and combinatorial optimization is applied to select the meta-learner model. This approach not only enhances accuracy but also mitigates the risk of overfitting and increases model flexibility.

The experimental results demonstrate that the RF-stacking ensemble learning model, constructed using KOA-DT, KOA-SVM, KOA-KNN, KOA-MLP, KOA-RF, and KOA-GBDT as base learners, with RF as the meta-learner, outperforms other baseline models in terms of accuracy, precision, recall, F1-score, and average AUC values, which are 0.9071, 0.9145, 0.9074, 0.9077, and 0.9886,

The experimental results confirmed the effectiveness and practicality of the proposed method, offering a novel technical approach for the rapid, accurate, and non-destructive classification of mung bean seeds. These findings are not only significant for mung bean seed classification but also serve as a reference for the rapid classification and quality assessment of other agricultural products through transfer learning. Moving forward, further investigation will focus on more efficient feature selection and model optimization techniques to enhance the performance of the classification model and broaden its applications in agriculture and other sectors.

Reference

1. Ganesan K, Xu B. A critical review on phytochemical profile and health promoting effects of mung bean (Vigna radiata). Food Science and Human Wellness. (2018) 7(1):11–33. doi: 10.1016/j.fshw.2017.11.002
2. Du M, Xie J, Gong B, Xu X, Tang W, Li X, et al. Extraction, physicochemical characteristics and functional properties of Mung bean protein. Food Hydrocolloids. (2018) 76:131 ‑ 40. doi: 10.1016/j.foodhyd.2017.01.003
3. Wu N, Liu F, Meng F, Li M, Zhang C, He Y. Rapid and Accurate Varieties Classification of Different Crop Seeds Under Sample-Limited Condition Based on Hyperspectral Imaging and Deep Transfer Learning. Frontiers in Bioengineering and Biotechnology (2021) 9:696292. doi: 10.3389/fbioe.2021.696292
4. Pathak N, Singh MN, Mishra MK, Saroj SK. Nature of gene action for yield and its components in mungbean (Vigna radiata). The Indian Journal of Agricultural Sciences. (2015) 85(4):504–8. doi: 10.56093/ijas.v85i4.47915
5. Bhardwaj HL, Hamama AA. Cultivar, Planting Date, and Row Spacing Effects on Mungbean Seed Composition. Journal of Agricultural Science. (2016) 8(10):26. doi: 10.5539/jas.v8n10p26
6. Benlioglu B, Ozkan U. Multivariate Analysis and its Application for Screening Mungbean [Vigna radiata (L.) Wilczek] Landraces. Legume Research. (2022). doi: 10.18805/LRF-661
7. Zhao Q, Chen Z, Khalifa MAS, Yin F, Qu X, Zhang J, et al. DNA Fingerprinting Construction and Genetic Diversity Analysis for Azuki Bean (Vigna angularis) and Mung Bean (Vigna radiata L.) Legume Research. (2022). doi: 10.18805/LRF-652
8. Xiao Z, Wang J, Han L, Guo S, Cui Q. Application of Machine Vision System in Food Detection. Frontiers in Nutrition. (2022) 9:888245. doi: 10.3389/fnut.2022.888245
9. Chen J, Wang Y, Luo L, Yu JG, Ma J. Image retrieval based on image-to-class similarity. Pattern Recognition Letters. (2016) 83:379–87. doi: 10.1016/j.patrec.2016.01.017
10. Liu H, Xu Z. Editorial: Machine vision and machine learning for plant phenotyping and precision agriculture. Frontiers in Plant Science. (2023) 14:1331918. doi: 10.3389/fpls.2023.1331918
11. Guo X, Ge Y, Liu F, Yang J. Identification of maize and wheat seedlings and weeds based on deep learning. Frontiers in Earth Science. (2023) 11:1146558. doi: 10.3389/feart.2023.1146558
12. Feng Y, Liu C, Han J, Lu Q, Xing X. Identification of wheat seedling varieties based on MssiapNet. Frontiers in Plant Science. (2024) 14:1335194. doi: 10.3389/fpls.2023.1335194
13. Li J, Xu F, Song S, Qi J. A maize seed variety identification method based on improving deep residual convolutional network. Frontiers in Plant Science. (2024) 15:1382715. doi: 10.3389/fpls.2024.1382715
14. Ember KJI, Hoeve MA, McAughtrie SL, Bergholt MS, Dwyer BJ, Stevens MM, et al. Raman spectroscopy and regenerative medicine: a review. npj Regenerative Medicine. (2017) 2(1):12. doi: 10.1038/s41536-017-0014-3
15. Wu L, Tang X, Wu T, Zeng W, Zhu X, Hu B, et al. A review on current progress of Raman-based techniques in food safety: From normal Raman spectroscopy to SESORS. Food Research International. (2023) 169:112944. doi: 10.1016/j.foodres.2023.112944
16. Farber C, Kurouski D. Raman Spectroscopy and Machine Learning for Agricultural Applications: Chemometric Assessment of Spectroscopic Signatures of Plants as the Essential Step Toward Digital Farming. Frontiers in Plant Science. (2022) 13:887511. doi: 10.3389/fpls.2022.887511
17. Ambrose A, Lohumi S, Lee WH, Cho BK. Comparative nondestructive measurement of corn seed viability using Fourier transform near-infrared (FT-NIR) and Raman spectroscopy. Sensors and Actuators B: Chemical. (2016) 224:500–6. doi: 10.1016/j.snb.2015.10.082
18. Czekus B, Pećinar I, Petrović I, Paunović N, Savić S, Jovanović Z, et al. Raman and Fourier transform infrared spectroscopy application to the Puno and Titicaca cvs. of quinoa seed microstructure and perisperm characterization. Journal of Cereal Science. (2019) 87:25–30. doi: 10.1016/j.jcs.2019.02.011
19. Jin Y, Tian H, Gao Z, Yang G, Dong D. Oil content analysis of corn seeds using a hand-held Raman spectrometer and spectral peak decomposition algorithm. Frontiers in Plant Science. (2023) 14:1174747. doi: 10.3389/fpls.2023.1174747
20. Zhu T, Liu Q, Zhang L. Hyperspectral Image Classification Using Multi-feature Fusion Residual Hypergraph Convolution Network. In: 2023 8th International Conference on Signal and Image Processing (ICSIP). Wuxi: IEEE; (2023) p. 343–9. doi: 10.1109/ICSIP57908.2023.10271030
21. Bi C, Zhang S, Chen H, Bi X, Liu J, Xie H, et al. Non-Destructive Classification of Maize Seeds Based on RGB and Hyperspectral Data with Improved Grey Wolf Optimization Algorithms. Agronomy. (2024) 14(4):645. doi: 10.3390/agronomy14040645
22. Lussier F, Thibault V, Charron B, Wallace GQ, Masson JF. Deep learning and artificial intelligence methods for Raman and surface-enhanced Raman scattering. TrAC Trends in Analytical Chemistry. (2020) 124:115796. doi: 10.1016/j.trac.2019.115796
23. Quinlan JR. Induction of decision trees. Machine Learning. (1986) 1(1):81–106. doi: 10.1007/BF00116251
24. Cortes C, Vapnik V. Support-vector networks. Machine Learning. (1995) 20(3):273–97. doi: 10.1007/BF00994018

25. Cover T, Hart P. Nearest neighbor pattern classification. IEEE Transactions on Information Theory. (1967) 13(1):21–7. doi: 10.1109/TIT.1967.1053964
26. Shahhosseini M, Hu G, Archontoulis SV. Forecasting Corn Yield With Machine Learning Ensembles. Frontiers in Plant Science. (2020) 11:1120. doi: 10.3389/fpls.2020.01120
27. Webb GI, Zheng Z. Multistrategy ensemble learning: reducing error by combining ensemble learning techniques. IEEE Transactions on Knowledge and Data Engineering. (2004) 16(8):980–91. doi: 10.1109/TKDE.2004.29
28. He Y, Zhang G, Gao Q. A novel ensemble learning method for crop leaf disease recognition. Frontiers in Plant Science. (2024) 14:1280671. doi: 10.3389/fpls.2023.1280671
29. Pugh NA, Young A, Ojha M, Emendack Y, Sanchez J, Xin Z, et al. Yield prediction in a peanut breeding program using remote sensing data and machine learning algorithms. Frontiers in Plant Science. (2024) 15:1339864. doi: 10.3389/fpls.2024.1339864
30. Yang L, Yu X, Zhang S, Zhang H, Xu S, Long H, et al. Stacking-based and improved convolutional neural network: a new approach in rice leaf disease identification. Frontiers in Plant Science. (2023) 14:1165940. doi: 10.3389/fpls.2023.1165940
31. R N, Sheela K G. Metaheuristic Algorithm Based Maximum Power Point Tracking Technique Combined With One Cycle Control For Solar Photovoltaic Water Pumping Systems. Frontiers in Energy Research. (2022) 10:902443. doi: 10.3389/fenrg.2022.902443
32. Xia D, Quan W, Wu T. Optimizing functional near-infrared spectroscopy (fNIRS) channels for schizophrenic identification during a verbal fluency task using metaheuristic algorithms. Frontiers in Psychiatry. (2022) 13:939411. doi: 10.3389/fpsyt.2022.939411
33. Qiao J, Wang C, Su G, Liang X, Dong S, Jiang Y, et al. Application research on the prediction of tar yield of deep coal seam mining areas based on PSO-BPNN machine learning algorithm. Frontiers in Earth Science. (2023) 11:1227154. doi: 10.3389/feart.2023.1227154
34. Zhu Z, Guan S. Prediction of the Height of Fractured Water-Conducting Zone Based on the Improved Cuckoo Search Algorithm–Extreme Learning Machine Model. Frontiers in Earth Science. (2022) 10:860507. doi: 10.3389/feart.2022.860507
35. Xia D, Quan W, Wu T. Optimizing functional near-infrared spectroscopy (fNIRS) channels for schizophrenic identification during a verbal fluency task using metaheuristic algorithms. Frontiers in Psychiatry. (2022) 13:939411. doi: 10.3389/fpsyt.2022.939411
36. Savitzky Abraham, Golay MJE. Smoothing and Differentiation of Data by Simplified Least Squares Procedures. Analytical Chemistry. (1964) 36(8):1627–39. doi: 10.1021/ac60214a047
37. Li H, Liang Y, Xu Q, Cao D. Key wavelengths screening using competitive adaptive reweighted sampling method for multivariate calibration. Analytica Chimica Acta. (2009) 648(1): 77–84. doi: 10.1016/j.aca.2009.06.046
38. Abdel-Basset M, Mohamed R, Azeem SAA, Jameel M, Abouhawwash M. Kepler optimization algorithm: A new metaheuristic algorithm inspired by Kepler's laws of planetary motion. Knowledge-Based Systems. (2023) 268:110454. doi: 10.1016/j.knosys.2023.110454
39. Xu C, Ding J, Qiao Y, Zhang L. Tomato disease and pest diagnosis method based on the Stacking of prescription data. Computers and Electronics in Agriculture. (2022) 197:106997. doi: 10.1016/j.compag.2022.106997
40. Jiang H, Zhang S, Yang Z, Zhao L, Zhou Y, Zhou D. Quality classification of stored wheat based on evidence reasoning rule and stacking ensemble learning. Computers and Electronics in Agriculture. (2023) 214:108339. doi: 10.1016/j.compag.2023.108339

Supporting information

S1 Appendix. Raman spectral data collection procedure

S2 Appendix. Image features collection procedure

Response to Reviewers:

Thank you very much for taking the time to carefully review our manuscript and suggest valuable amendments. In response to the review opinions raised by reviewing experts, we first answered each question, and then revised the corresponding content in the paper according to the expert's comments (the revised part has been marked in red in the paper)

Point 1: When submitting your revision, we need you to address these additional requirements.Please ensure that your manuscript meets PLOS ONE's style requirements, including those for file naming.

Response 1: Thanks for your valuable comments.I formatted the article according to the journal's guidelines.

For example:Added line numbers to the title

1 **Mung bean seed classification based on multimodal features**
2 **and Kepler-optimized stacking ensemble learning model**

Point 2: Please note that PLOS ONE has specific guidelines on code sharing for submissions in which author-generated code underpins the findings in the manuscript. In these cases, we expect all author-generated code to be made available without restrictions upon publication of the work.

Response 2: Thank you for your valuable comments. In response, I have uploaded the complete code implementation with comprehensive annotations to GitHub. Both the source code and corresponding datasets are included in the same repository to ensure full reproducibility for the research community.

GitHub:https://github.com/781118954/multi-data

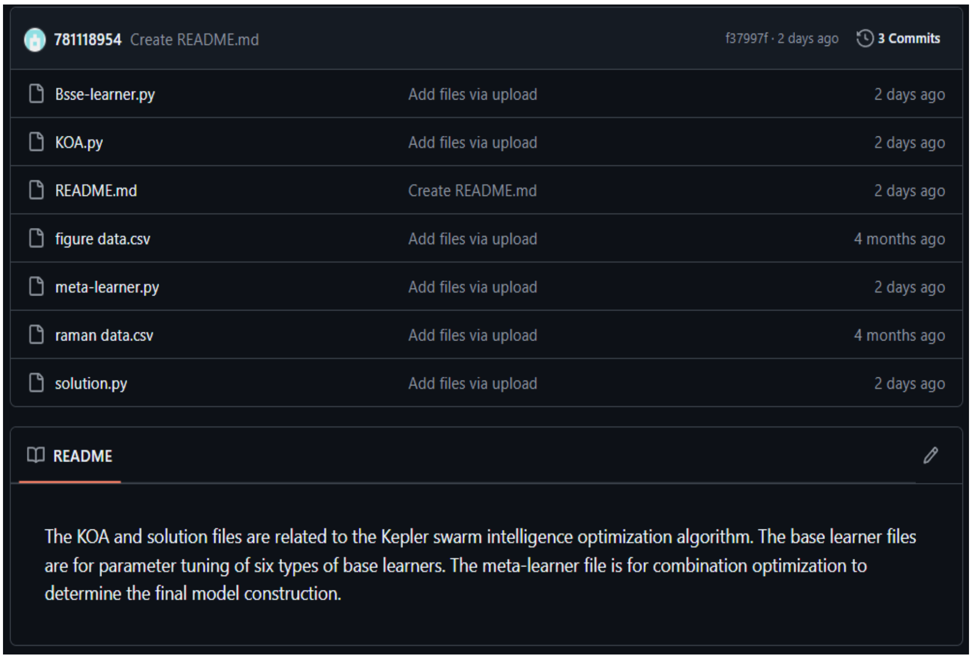

Point 3: Please include captions for your Supporting Information files at the end of your manuscript, and update any in-text citations to match accordingly.

Response 3: Thanks for your valuable comments.I have added the captions for the Supporting Information at the end of the manuscript and made the necessary in-text citations accordingly.

1.Captions for Supporting information:

Supporting information

S1 Appendix. Raman spectral data collection procedure

S2 Appendix. Image features collection procedure

2.Corresponding in-text citations

to 1800 cm^{-1}, yielding a total of 700 distinct Raman spectral features.The Raman spectral data collection procedure is described in S1 Appendix.

texture features, and 18 color features. The image features collection procedure is described in S2 Appendix.

Point 4(Additional Editor Comments): The images are completely blurred, making it difficult to interpret the visual data accurately. Additionally, the modeling of the proposed method requires further clarification. It should be explained in a way that enhances understanding and benefits readers from various backgrounds. Please address these issues carefully to ensure that the manuscript meets the quality standards of PLOS ONE.

Response 4: Thank you for your valuable feedback. Regarding the image resolution issue, I have updated the figure files using the Preflight Analysis and Conversion Engine (PACE) digital diagnostic tool provided by the journal, and they have been successfully re-uploaded. Additionally, the original composite image has been split into individual images and uploaded separately.

To address the need for further clarification of the modeling process, a detailed explanation has been incorporated between the theoretical framework and experimental sections to ensure comprehensibility for readers across diverse disciplinary backgrounds.

After verification:

3. Results and discussion

The stacking ensemble learning model adopted in this paper consists of two layers: base learners and a meta-learner. It fuses the prediction results of multiple base learners of different types and then conducts final integration through the meta-learner. By leveraging the complementarity among models, it captures the characteristics and patterns of data from multiple perspectives, effectively reducing errors and significantly improving the accuracy and generalization ability of prediction and classification. In view of this, selecting high - performing base learner models and

meta-learner models is of great importance. In this paper, the KOA is first applied to optimize the parameters of six classification models,including SVM, DT, KNN, RF, MLP, and AdaBoost. The optimized models are then used as the base learners of the stacking ensemble learning model. Subsequently, the meta-learner model is selected through a combinatorial optimization approach, and finally, the stacking ensemble learning model used in this paper is constructed.

The hardware specifications employed in this experiment are as follows: the processor is an Intel Core i5-12400F with a clock speed of 2.50 GHz, the system is equipped with 16 GB of RAM, and the graphics card utilized is an NVIDIA RTX 4060 Ti.

Data Review URL

Click here to download Data Review URL
http://github.com/781118954/multi-data
