# Supplementary material for: Mung bean seed classification based on multimodal features and Kepler-optimized stacking ensemble learning model
Source: PLoS One. 2026 Jan 5;21(1):e0338928. doi: 10.1371/journal.pone.0338928 (PMC12768288; doi:10.1371/journal.pone.0338928)
Supplement: Attachment — Submitted filename: Response to Reviewers.docx [file pone.0338928.s005.docx]

**Response to Reviewers:**

Thank you very much for taking the time to carefully review our manuscript and suggest valuable amendments. In response to the review opinions raised by reviewing experts, we first answered each question, and then revised the corresponding content in the paper according to the expert's comments (the revised part has been marked in red in the paper)

**Point 1:**When submitting your revision, we need you to address these additional requirements.Please ensure that your manuscript meets PLOS ONE's style requirements, including those for file naming.

**Response 1:**Thanks for your valuable comments.I formatted the article according to the journal's guidelines.

For example:Added line numbers to the title

**Point** 2**:**Please note that PLOS ONE has specific guidelines on code sharing for submissions in which author-generated code underpins the findings in the manuscript. In these cases, we expect all author-generated code to be made available without restrictions upon publication of the work.

**Response 2:**Thank you for your valuable comments. In response, I have uploaded the complete code implementation with comprehensive annotations to GitHub. Both the source code and corresponding datasets are included in the same repository to ensure full reproducibility for the research community.

GitHub:https://github.com/781118954/multi-data

**Point 3:**Please include captions for your Supporting Information files at the end of your manuscript, and update any in-text citations to match accordingly.

**Response 3:**Thanks for your valuable comments.I have added the captions for the Supporting Information at the end of the manuscript and made the necessary in-text citations accordingly.

1.Captions for Supporting information:

2.Corresponding in-text citations

**Point 4(Additional Editor Comments):**The images are completely blurred, making it difficult to interpret the visual data accurately. Additionally, the modeling of the proposed method requires further clarification. It should be explained in a way that enhances understanding and benefits readers from various backgrounds. Please address these issues carefully to ensure that the manuscript meets the quality standards of PLOS ONE.

**Response 4:** Thank you for your valuable feedback. Regarding the image resolution issue, I have updated the figure files using the Preflight Analysis and Conversion Engine (PACE) digital diagnostic tool provided by the journal, and they have been successfully re-uploaded. Additionally, the original composite image has been split into individual images and uploaded separately.

To address the need for further clarification of the modeling process, a detailed explanation has been incorporated between the theoretical framework and experimental sections to ensure comprehensibility for readers across diverse disciplinary backgrounds.

**After verification：**

**3.	Results and discussion**

The stacking ensemble learning model adopted in this paper consists of two layers: base learners and a meta-learner. It fuses the prediction results of multiple base learners of different types and then conducts final integration through the meta-learner. By leveraging the complementarity among models, it captures the characteristics and patterns of data from multiple perspectives, effectively reducing errors and significantly improving the accuracy and generalization ability of prediction and classification. In view of this, selecting high - performing base learner models and meta-learner models is of great importance. In this paper, the KOA is first applied to optimize the parameters of six classification models,including SVM, DT, KNN, RF, MLP, and AdaBoost. The optimized models are then used as the base learners of the stacking ensemble learning model. Subsequently, the meta-learner model is selected through a combinatorial optimization approach, and finally, the stacking ensemble learning model used in this paper is constructed.

The hardware specifications employed in this experiment are as follows: the processor is an Intel Core i5-12400F with a clock speed of 2.50 GHz, the system is equipped with 16 GB of RAM, and the graphics card utilized is an NVIDIA RTX 4060 Ti.
