# Supplementary material for: Mung bean seed classification based on multimodal features and Kepler-optimized stacking ensemble learning model
Source: PLoS One. 2026 Jan 5;21(1):e0338928. doi: 10.1371/journal.pone.0338928 (PMC12768288; doi:10.1371/journal.pone.0338928)
Supplement: Attachment — Submitted filename: Response_to_Reviewers_auresp_2.docx [file pone.0338928.s007.docx]

**Response to Reviewers:**

Thank you very much for taking the time to carefully review our manuscript and suggest valuable amendments. In response to the review opinions raised by reviewing experts, we first answered each question, and then revised the corresponding content in the paper according to the expert's comments (the revised part has been marked in red in the paper)

**Point 1:Explain about Raman Spectroscopy and its paragraph from 78 to 88 line numbe should be here.**

**/The introducton can be more chronological.**

**Response 1:**Thanks for your valuable comments.I have revised the sequence of the introduction section.:

“Raman spectroscopy addresses the limitations of traditional methods by leveraging its rapid, non-destructive analytical capabilities [8]. This technique operates on the principle of inelastic light scattering and has been widely applied to plant variety identification and nutrient analysis [9,10], including assessing maize kernel viability [11], analyzing quinoa seed nutrients [12], and quantifying maize oil content [13]. While effective for evaluating inherent qualities, Raman spectroscopy exhibits limited utility in analyzing morphological characteristics. Computer vision employs image processing and analysis techniques to deliver precise and efficient solutions for plant phenotyping [14,15]. This technology predominantly relies on distinguishing features such as color, texture, and shape [16]. Notably, it has demonstrated successful applications in crop-weed identification [17], wheat variety identification [18], and maize seed identification [19]. However, its effectiveness hinges critically on the visual integrity of the target samples.”

**Point** 2**:write sentence structure like that " The study adresses the knowledge gap". The objective should not be mentioned as novel appproach or like that . /Write the objective in better way.**

**The objectives needs to be written better.**

**/Write this objectie in more simpler way**

**Response 2:**Thank you for your valuable comments. In response, I have rewritten the research objectives using the writing structure you suggested.

“(1). This study addresses the limitations of explainable analysis in the field of mung bean seed classification.The Raman spectral features and image numerical features are utilized for the inherent qualities and phenotypic analysis of mung bean seeds, respectively.

(2). This study addresses the limitations of existing mung bean seed datasets in thoroughly and precisely extracting features.The Raman spectroscopy features and numerical image features are extracted separately, then a multimodal dataset is constructed using a front-end fusion approach.

(3). This study addresses the limitations of existing model parameter optimization algorithms. The key parameters of the base learner in the stacking ensemble learning model are optimized utilizing the Kepler optimization algorithm (KOA) .

(4). This study addresses the limitations in the ensemble stacking model construction process of existing methods. Machine learning models are optimized to function as base learners, while the meta-learner model is selected through a combined optimization strategy.”

**Point 3:first write environment in which all these algorithm are processed with version of it used. Incluse the library used with its link to published pdf for help.**

**/The author needs to provide links to packages and functions used. The author can give link to the standard pdf used for the library used in the manuscript.**

Response 3:Thanks for your valuable comments.I have provided the specifications of the hardware, software versions, and key libraries employed in this experiment, along with their relevant links.

“2.1Computational Environment

The hardware specifications employed in this experiment are as follows: the processor is an Intel Core i5-12400F with a clock speed of 2.50 GHz, the system is equipped with 16 GB of RAM, and the graphics card utilized is an NVIDIA RTX 4060 Ti.

The software consisted of the Windows 11 operating system, Python 3.11.5, and the following primary libraries: scipy 1.11.1, scikit-learn 1.3.0, and mlxtend 0.23.1. Links to the packages and functions used in this study are provided at: https://github.com/781118954/multi-data.”

**Point 4:mention all the package and environmnet used for executing these package with the function to call for the algorithm**

**/write specific function with the packages used in each step of method**

**/The execution of the algorithm and its environment is missing.**

**/mention library with function used to execure for every major algorithm and accuracy analysis**

**Response 4:** Thank you for your valuable feedback. I have specified the required libraries and functions for each individual algorithm.

“The raw Raman spectra features were first smoothed using a Savitzky-Golay (SG) filter, implemented via the savgol_filter function from the scipy.signal module, to reduce noise for subsequent analysis [37].”

“This step is primarily implemented using the train_test_split function from the sklearn.model_selection library.”

“This step is primarily implemented by initializing the classifiers parameter with base-learners within the StackingCVClassifier function from the mlxtend.classifier library.”

“This step is primarily implemented by initializing the cv parameter within the StackingCVClassifier function from the mlxtend.classifier library.”

“This step is primarily implemented by initializing the meta_classifier parameter with meta-learners within the StackingCVClassifier function from the mlxtend.classifier library.”

**Point 5:no need to write "WE" ."The spectra was preprocessed"**

**Response 5:** Thank you for your valuable feedback. I have revised the text using the sentence structures you suggested.

“Preprocessing operations were performed on the acquired raw Raman spectral features and image numerical features, respectively.”

**Point 6:check all the equation,**

**Response 6:**Thank you for your valuable feedback.I have double-checked all the formulas, ensuring they are properly formatted and use the Times New Roman font.

$$\text{r}_{\text{i}}\text{=}\text{a}\text{e}^{\text{−}\text{ki}}$$

(1)

**Point 7:There is only elaboration of results in whole section , the result significance with reference to previous research are discussed with the results.**

**/The discussions and conclusions needs to be improved**

**Response 7:** Thank you for your valuable feedback. I have revised the text using the sentence structures you suggested.

(1).A qualitative analysis was performed on the extracted Raman spectral features.

(2) The multimodal dataset was compared with previous single Raman spectral datasets, and the reasons for the differences were analyzed.

(3) The phenomenon of high iteration counts during KOA-optimized SVM parameter search was analyzed.

(4) The performance of the stacked ensemble model constructed in this study was compared with other ensemble learning models, and the underlying reasons were discussed.

“The Raman spectral features selected by the CARS algorithm in this work provide a statistically robust and chemically interpretable basis for classifying mung bean seeds. Peaks at 1444$\mathrm{cm}^{-1}$ and 1446$\mathrm{cm}^{-1}$ are unequivocally assigned to C-H bending vibrations, serving as a fingerprint for protein structure [40]. This critical assignment directly connects spectral data to the seed's biochemical makeup—specifically, protein content. Therefore, the high classification accuracy achieved in this study stems not from mere data fitting, but from the successful translation of molecular vibrational information into a discriminative basis for the machine learning model, thereby grounding the classification results in sound physicochemical principles.”

“Experimental results indicate that the multimodal dataset constructed in this study significantly outperforms single Raman spectral dataset in characterizing mung bean seeds [41]. This is attributed to the method's integration of Raman spectroscopy revealing internal chemical composition with image information reflecting external physical morphology, followed by effective redundancy removal through feature extraction, ultimately achieving a comprehensive, accurate, and concise characterization of seed features.”

“The optimization of SVM parameters necessitates extensive iterations due to the strong coupling between regularization parameter C and gamma. These parameters collectively determine the algorithm's bias-variance trade-off: gamma redefines the hypothesis space, which in turn shifts the optimal complexity level achievable by C [44]. This interdependence makes C's optimum contingent upon gamma's current value, transforming the optimization into a non-convex cooperative problem characterized by a response surface with abundant local optima and a globally optimal solution confined to a narrow gorge-like region, demanding highly synchronized parameter tuning.”

“Experimental results demonstrate that the proposed stacking ensemble learning model significantly outperforms individual RF and GBDT methods in mung bean seed classification tasks [45,46]. The performance enhancement is achieved through the integration of complementary characteristics from base learners. Specifically, RF exhibits powerful capability in capturing feature interactions, enabling comprehensive exploration of nonlinear relationships within the data, while GBDT demonstrates exceptional performance in modeling complex decision boundaries through efficient gradient optimization strategies. This synergistic interaction substantially enhances the model's generalization capability. Furthermore, at the architectural level, the stacking ensemble achieves superior bias-variance balance through its hierarchical framework, and employs meta-learners to intelligently integrate outputs from base models, thereby establishing a more equilibrium state between bias and variance.”

**Point 8:all the statistical and accuracy analysis statistical formula should be mentione in material section as a seprate heading**

**Response 8:**Thank you for your valuable feedback.I have provided a detailed description of all statistical and accuracy analysis methods involved in this study.

“2.7Evaluation metrics

The performance for a target class A is evaluated based on four counts: true positives (TP) are samples from class A that are correctly identified; false positives (FP) are samples from other classes erroneously assigned to class A; true negatives (TN) are samples from other classes correctly identified as not belonging to class A; and false negatives (FN) are samples from class A that are incorrectly rejected.Based on the aforementioned fundamental statistics, the following core evaluation metrics can be defined:

(1). Accuracy measures the proportion of correctly classified instances relative to the entire dataset. Its formula is provided in Eq. (6)

$$\text{accuracy}\text{=}\frac{\text{TP}\text{+}\text{TN}}{\text{TP}\text{+}\text{TN}\text{+}\text{FP}\text{+}\text{FN}}$$

(6)

(2). Precision quantifies the proportion of true positives among all instances labeled as positive, reflecting the reliability of positive predictions. It is defined by Eq. (7).

$$\text{precision}\text{=}\frac{\text{TP}}{\text{TP}\text{+}\text{FP}}$$

(7)

(3). Recall, also known as sensitivity, represents the ratio of true positives to all actual positive instances. This metric evaluates the model's capacity to identify all relevant cases and is formulated in Eq. (8).

$$\text{recall}\text{=}\frac{\text{TP}}{\text{TP}\text{+}\text{FN}}$$

(8)

(4). F1-Score (F1) serves as the harmonic mean of precision and recall, offering a balanced evaluation metric particularly useful for datasets with class imbalance. Its calculation follows Eq. (9).

$$\text{F}\text{1}\text{-}\text{S}\text{core}\text{=}\frac{\text{2}\text{×}\text{precision}\text{×}\text{recall}}{\text{precision}\text{+}\text{recall}}$$

(9)

”

**Point 9:write short comings and challeneges faced which can be improved in fututre at the end of conclusion in last paragraph**

**Response 9:**Thank you for your valuable feedback.I have outlined the limitations of this study and prospects for future research.

“Future research will primarily focus on the following three directions. First, at the model architecture level, the introduction of lightweight deep learning models aims to overcome the limitations of traditional feature engineering and achieve efficient end-to-end feature learning. Second, at the data modality level, efforts will be dedicated to constructing a universal framework capable of flexibly integrating multi-source information, such as near-infrared spectroscopy and fluorescence spectroscopy, thereby transcending the current constraint of solely combining Raman spectral features and image numerical features. Finally, at the application scope level, the study plans to extend the model specifically designed for mung bean classification in this research into a universal intelligent detection solution applicable to various crops through transfer learning techniques.”
